# A PBMC-based proof-of-principle framework for fit-for-purpose validation of flow cytometry assays in cell therapy development

**DOI:** 10.3389/fmedt.2026.1905186

**Published:** 2026-10-14

**Authors:** Huixiu Han, Anita Schmitt, Angela Hückelhoven-Krauss, Brigitte Neuber, Theresa Tretter, Mandy Hinkelbein, Ann-Kathrin Bauer, Carsten Müller-Tidow, Michael Schmitt, Lei Wang

**Affiliations:** 1 Department of Hematology, Oncology and Rheumatology, Heidelberg University Hospital, Heidelberg, Germany; 2 Department of Hematology, Beijing Tiantan Hospital, Capital Medical University, Beijing, China

**Keywords:** ATMPs, cellular therapy, flow cytometry, method validation, potency assay

## Abstract

**Introduction:**

Cellular immunotherapy offers personalized therapeutic benefits but faces challenges such as manufacturing consistency and regulatory compliance. Validated analytical methods based on product's mechanism of action are essential to guarantee product quality and patient safety. Despite regulatory emphasis on analytical validation, a structured framework applicable to broader cellular therapies remains undefined.

**Methods:**

This study evaluated a PBMC-based proof-of-principle framework for fit-for-purpose validation of flow cytometry-based analytical method to cellular therapy development. Key assay parameters were optimized, including antibody concentration, photomultiplier tube (PMT) voltage, compensation, and steric hindrance verification. Assay validation assessed specificity, sensitivity, linearity, precision, robustness, and carryover across different conditions.

**Results:**

The validated method demonstrated high specificity (false-positive rates <1%), strong assay sensitivity (LLOQ within acceptable limits), robust linearity (R^2^ > 0.9), and reproducibility across intra-assay, inter-instrument, and inter-operator evaluations. Practical considerations, including sample stability, antibody cocktail preparation, and instrument carryover, were also addressed to enhance workflow efficiency.

**Discussion:**

The proposed framework may support product-specific analytical method development; however, application to a defined cell therapy producet requires product-, matrix-, and analytical decision-specific qualification or validation.

## Introduction

Cellular immunotherapy, a subset of advanced therapy medicinal products (ATMPs), has emerged as a transformative treatment approach, offering significant therapeutic benefits across various diseases (1). Unlike traditional pharmaceuticals, these therapies rely on living cells, often personalized to individual patients, making manufacturing, consistency, and standardization particularly challenging (2). The inherent heterogeneity of patient- and donor-derived materials further complicates batch-to-batch reproducibility, necessitating robust analytical methods to ensure product quality and patient safety.

While regulatory guidelines emphasize comprehensive ATMP characterization, a stepwise framework for analytical validation remains undefined (3). Given the complexity of cell-based products, regulatory authorities advocate for a case-by-case approach, requiring developers to tailor assay validation strategies and frequently seek scientific advice from regulatory bodies (3, 4).

Most existing analytical method validation studies focus on chimeric antigen receptor T (CAR-T) cell therapies (5), leaving a gap in standardized validation approaches for broader cellular immunotherapies. This study therefore aimed to establish a PBMC-based proof-of-principle framework for fit-for-purpose validation of flow cytometryassays, using major leukocyte populations and apoptotic cell fractions as representative analytical measurements. The framework is intended as a methodological template for product specific method development, while the selection of cellular markers, critical quality attributes, acceptance criteria, and analytical performance requirements should be tailored to the intended product and stage of manufacturing.

## Results

### Titration of antibodies

To determine the optimal antibody volumes for 7AAD, Apotracker and CD66b, titration experiments were conducted by evaluating both the staining index (SI) and the percentage of positive cells (% pos) (Supplementary Figure S1). The optimal volume was as the concentration that provided the highest SI while maintaining a stable % pos.

For 7AAD, antibody volumes ranging from 3 μL to 5 μL yielded the highest SI and % pos (Supplementary Figure S1, upper panel). Among these, 4 μL was selected for further experiments due to its optimal balance between signal intensity and cell population detection.

Similarly, for Apotracker, the % pos. remained within an optimal range between 3 and 5 μL, with 5 μL showing the highest SI (Supplementary Figure S1, lower panel). Thus, 5 μL was chosen as the optimal volume.

### Determination of the optimal PMT voltage

To establish the optimal photomultiplier tube (PMT) voltage range, we analyzed coefficient of variation (CV), PMT linearity, signal-to-background ratio (SB), and SI across different detector voltages using eight-peak rainbow beads and unstained CompBeads.

In the APC channel, at lower voltages, dim peaks were difficult to distinguish, while at higher voltages, increased signal spread caused the brightest peaks to exceed the dynamic range (Supplementary Figure S2A, upper panel). The CV of M1 beads decreased with increasing voltage, reaching a plateau between 500 and 700 V. Additionally, PMT linearity was confirmed in the 450–700 V range, as indicated by the zero-slope region in the linearity curve (Supplementary Figure S2B, upper panel).

The unstained CompBeads were analyzed to quantify background noise at different voltages. As voltage increased, background signal levels increased, leading to higher MFI and variability in unstained controls (Supplementary Figure S2A, middle panel). The SB ratio, comparing the MFI of M1 beads to unstained CompBeads, was highest at lower voltages due to minimal background but declined as voltage increased. A plateau in SB was observed between 500 and 700 V, similar to the CV trend (Supplementary Figure S2B, middle panel).

To further refine voltage selection, SI was calculated using lymphocytes stained with APC at different voltages. At lower voltages, positive and negative populations were difficult to separate, while at higher voltages, increased spread was observed in the negative population (Supplementary Figure S2A, lower panel). SI increased with voltage until reaching a plateau at 550 V, beyond which additional voltage increments did not significantly improve signal resolution (Supplementary Figure S2B, lower panel).

Based on these observations, the optimal PMT voltage range was defined by the intersection of lowest CV, zero-slope linearity, and highest SB and SI values. In the APC channel, the optimal range was determined to be 550–700 V.

For other fluorochrome channels, the following voltage ranges were identified as optimal: FITC: 550–700 V, PE: 600–700 V, PE-Cy7: 600–700 V, APC-Cy7: 550–650 V, PerCP: 700 V, and APC-R7: 600 V (Supplementary Figure S2C). Representative flow cytometry dot plots illustrating voltage optimization across channels are presented in Supplementary Figure S3.

### Steric hindrance evaluation in multicolor staining panels

To evaluate steric hindrance in the staining panel, fluorescence intensities from fully stained samples were compared to single-antibody-stained controls. Flow cytometry histograms and dot plots demonstrated that fluorescence intensity remained consistent across most antibodies, indicating minimal steric hindrance (Figure 1A and Supplementary Figure S4).

**Figure 1:**
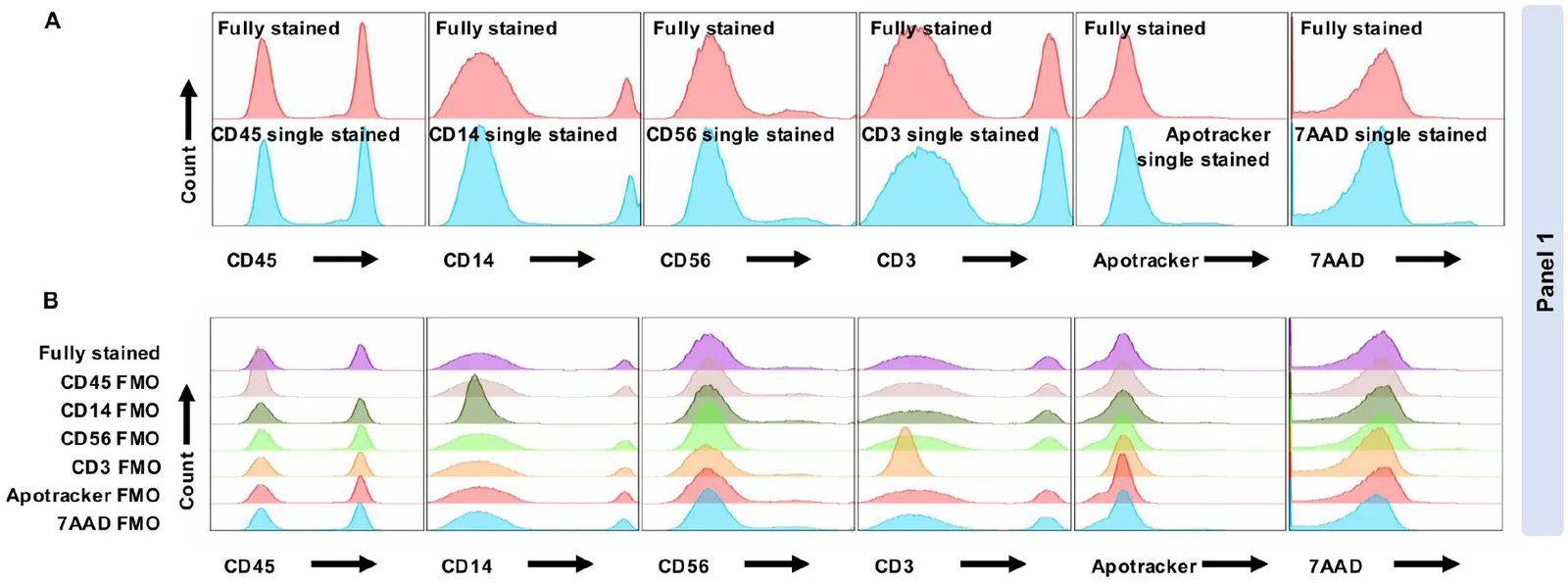
FMO analysis. **(A)** Flow cytometry histograms comparing fully stained versus single- antibody-stained samples. **(B)** Flow cytometry histograms comparing fully stained versus FMO controls across all antibody channels.

However, assessing Apotracker and 7AAD staining was challenging due to the low abundance of positive cells. A quantitative comparison of MFI and percentage of positive cells between single-stained and fully stained samples is summarized in Supplementary Table S1, S2. Most antibodies exhibited an acceptable CV% (<10%), except for 7AAD, which had a higher CV (54.8% in MFI and 22.4% in percentage), likely due to the low frequency of 7AAD + cells (Supplementary Table S1-S2).

### Compensation validation using fluorescence minus one (FMO) controls

Fluorescence Minus One (FMO) controls were used to evaluate compensation accuracy in all fluorescence channels (Figure 1B and Supplementary Figure S5). FMO controls showed minimal spillover, as their signal intensities closely matched those of the fully stained sample in their respective channels while remaining negative in the empty channels. These results confirmed proper compensation settings across the panel.

Steric hindrance and compensation assessments were also performed in an additional panel, panel 2, to further validate the robustness of the staining strategy. Similar trends were observed, confirming the absence of significant steric hindrance and appropriate compensation settings (data not shown).

### Assay specificity

To evaluate assay specificity, two independent runs were conducted using PBMCs from three healthy donors, fully stained with antibody cocktail. FMO controls were used as negative controls, where each sample was stained without a specific antibody to evaluate potential false positive events. The false positive rate was calculated by dividing the number of antibody-positive cells in FMO samples by those in fully stained samples.

As shown in Figures 2A,B, the false positive rates for CD14, CD3, CD45, and CD56 were below 1% meeting the specificity criterion. These results indicate minimal non-specific antibody binding, confirming high specificity for these markers.

**Figure 2:**
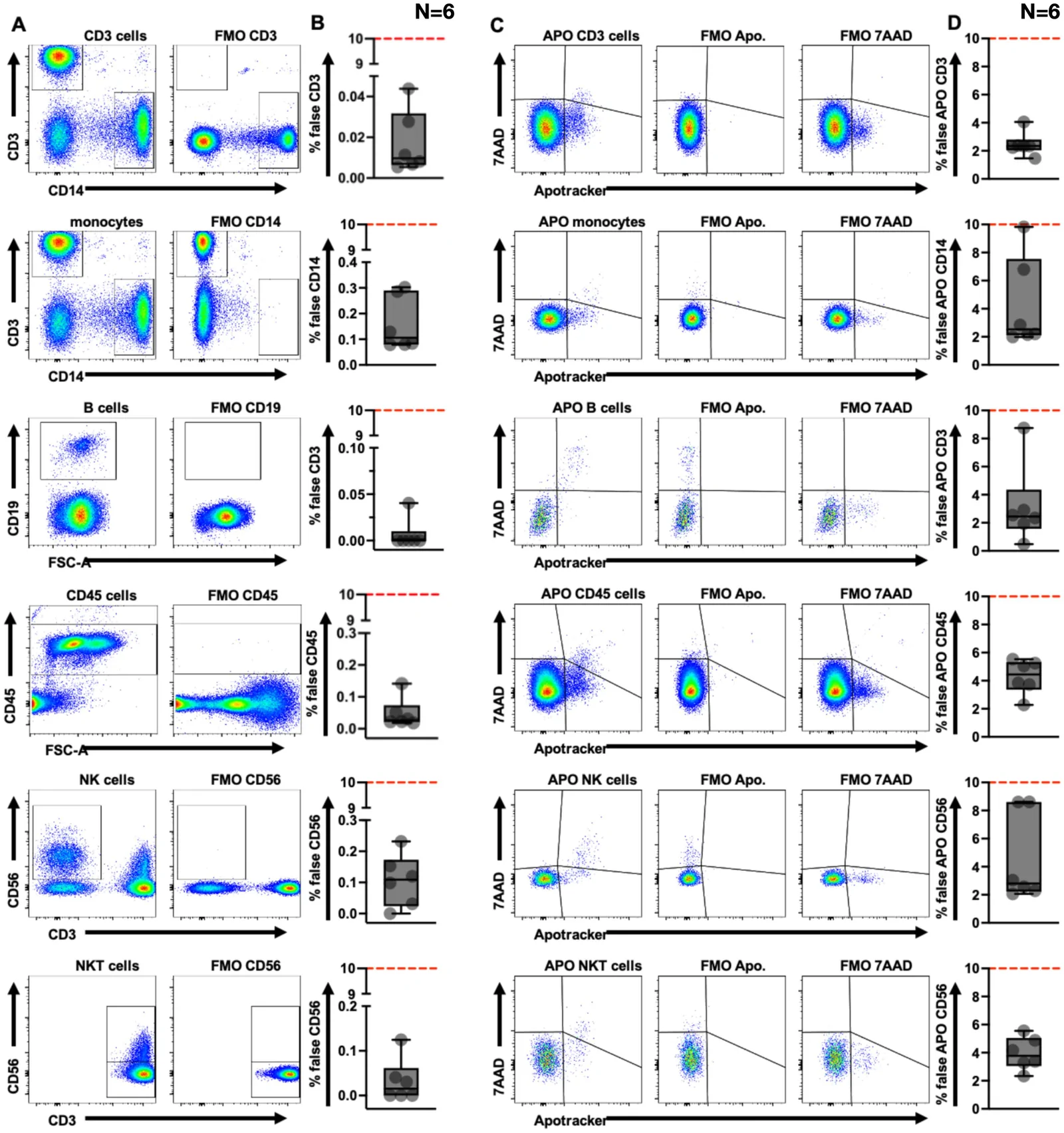
Assay specificity. **(A)** Representative dot plots of fully stained samples and FMO controls lacking the specific antibody of interest. **(B)** False positive rates were statistically calculated for each marker. The red dashed line indicates the specificity criterion (false positive rate < 10%). **(C)** Representative dot plots of apoptotic cells in fully stained samples and samples lacking Apotracker or 7AAD. **(D)** False apoptotic cell rates were calculated statistically, with the red dashed line indicating the 10% specificity threshold. (*n* = 6, data represent mean ± SD).

Additionally, the specificity of the Apotracker and 7AAD reagents for detecting apoptotic cells was evaluated by measuring false apoptotic events across different cell populations. Apoptotic cells were defined as Apotracker+7AAD + (late apoptotic) and Apotracker+7AAD- (early apoptotic) (6). To quantify false apoptotic labeling, the number of apoptotic cells detected in the absence of Apotracker was divided by the corresponding count in fully stained samples.

As illustrated in Figures 2C,D, all false apoptotic rates remained below 10%, meeting the specificity threshold. These findings confirm that Apotracker and 7AAD staining specifically detects apoptotic cells, with minimal false labeling, further supporting the overall assay specificity.

### Assay sensitivity

To evaluate the sensitivity of the assay, a dilution series was performed using stained cells spiked into unstained samples, followed by the determination of LOB, LOD, and LLOQ.

As shown in Figure 3, CD3+ T cell measurements remain precise, maintaining a CV below 30% at the LLOQ, even at a stained cell fraction as low as 0.1%, corresponding to 32.3 detected events. Additionally, the LLOQ values for other cell populations were determined as follows: CD14 + monocytes: 1% stained cell fraction (132.3 events), CD19+ B cells: 1% stained cell fraction (17 events), CD56+ NK cells: 0.1% stained cell fraction (4.3 events), and CD56 + CD3+ NK-T cells: 10% stained cell fraction (168.7 events).

**Figure 3:**
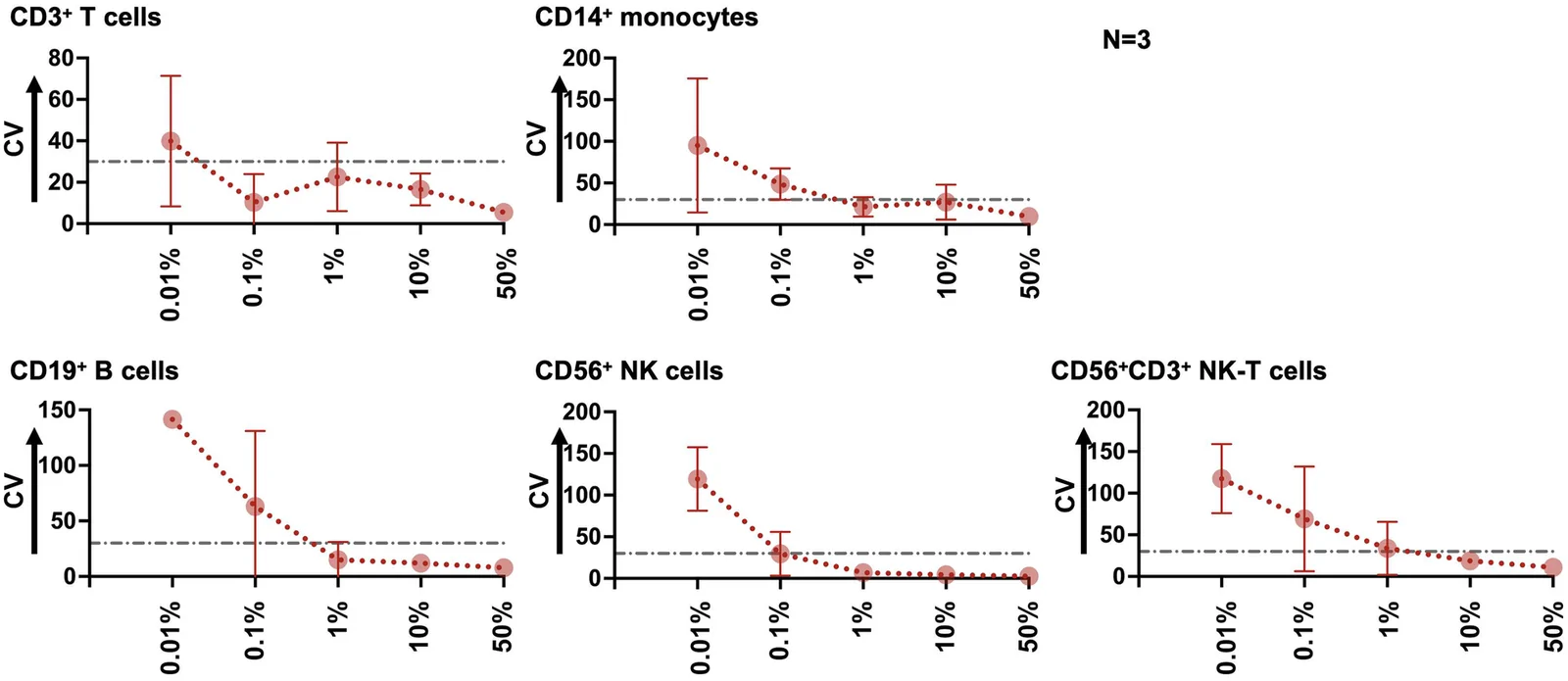
Assay sensitivity. Stained cells were serially diluted into unstained cells. The *X*-axis represents the percentage of stained cells in the dilution series, while the *Y*-axis shows the CV between observed and expected cell events. The accepted precision threshold (30% CV) is marked by a grey dashed line. Samples from three individual donors were tested. (*n* = 3, data represent mean ± SD).

The limit of blank (LOB) and the limit of detection (LOD) were calculated using blank and near-blank samples, with LOB and LOD values for T cells determined as 0 and 3.4 events, respectively. The corresponding values for other cell populations are presented in Table 1.

**Table 1:** The corresponding cell events of LOB, LOD, and LLOQ.

Parameter	T cells	Monocytes	B cells	NK cells	NK-T cells
LOB	0	1.6	0	0	0
LOD	3.4	3.5	0	1.9	0.9
LLOQ	32.3	132.3	17	4.3	168.7

### Linearity assessment

To evaluate linearity and detect potential prozone or hook effects, a dilution series was performed by diluting samples with buffer, and the linearity of dilution was assessed. The measured cell events, normalized using counting beads, were plotted against the predicted cell events on logarithmic scales (Figure 4).

**Figure 4:**
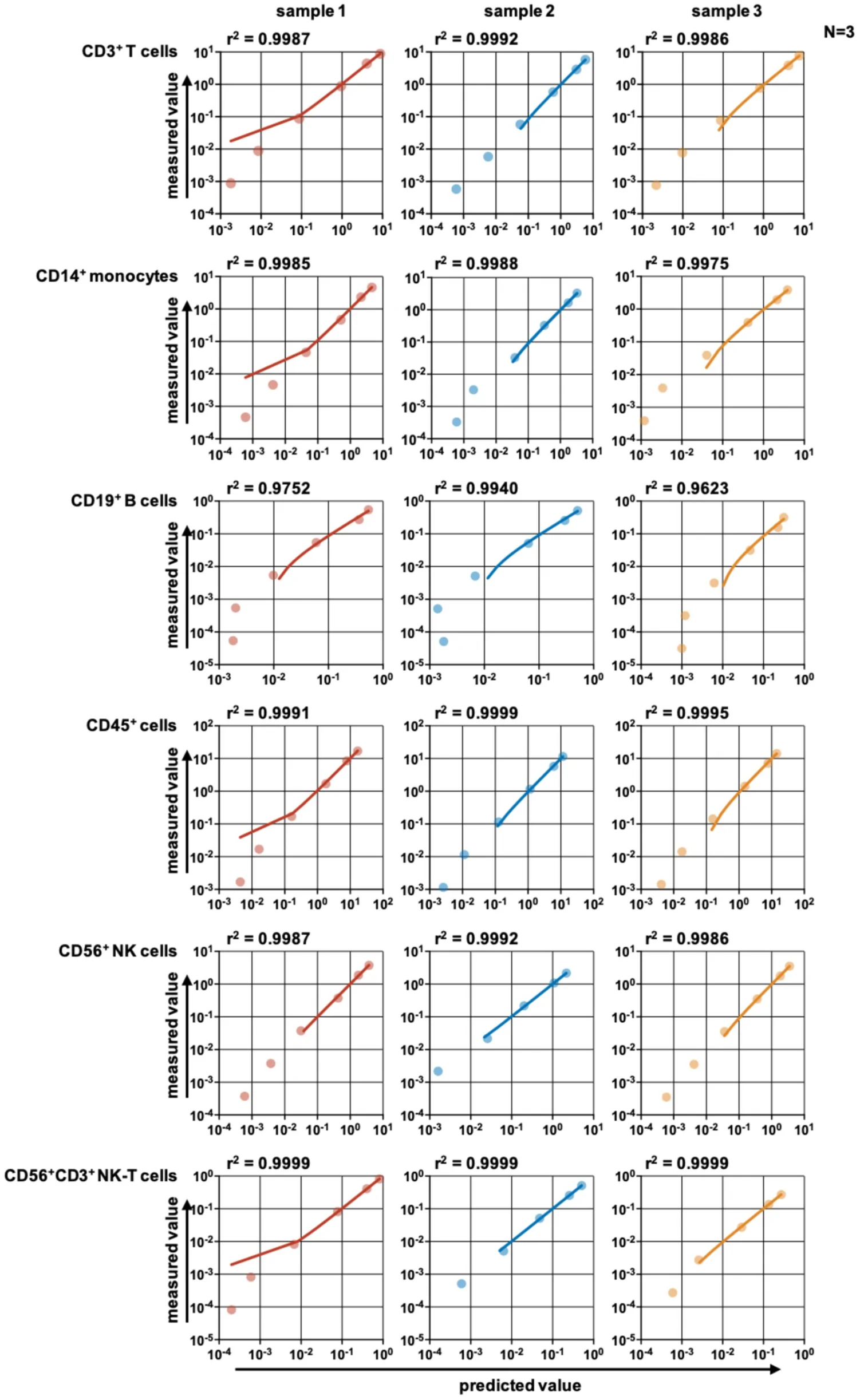
Linearity assessment. The measured values were plotted against expected values using logarithmic scales, and linear regressions were performed for each cell population. Data were obtained from three independent samples. (*n* = 3).

Linear regression analysis demonstrated a strong correlation (R^2^ > 0.95) across all cell populations, confirming assay linearity. Specifically, T cells, monocytes, NK cells, and NKT cells exhibited linearity at dilution ratios above 1:100. B cells maintained linearity when the dilution ratio exceeded 1:10.

Additionally, apoptotic cell populations showed strong linearity. Apoptotic T cells, monocytes, B cells, and NK cells remained linear at dilution ratios above 1:10. Apoptotic NKT cells exhibited linearity at dilution ratios above 1:100 (Supplementary Figure S6). Representative dot plots illustrating dilution effects across cell populations and apoptotic cells are shown in Supplementary Figure S7-S8.

### Intra-assay precision and repeatability

To evaluate the repeatability of the assay, intra-assay precision was assessed by analyzing each sample three times within a single analytical run. The CV was calculated for these replicates. The mean CV for each reportable result across three samples was also determined.

As shown in Figure 5A, the assay demonstrated high repeatability, with CV values below 10% for the main cell populations (T cells, monocytes, B cells, NK cells, and NKT cells). For apoptotic cell populations, which are considered rare events, the CV remained below 30%, meeting acceptable precision criteria.

**Figure 5:**
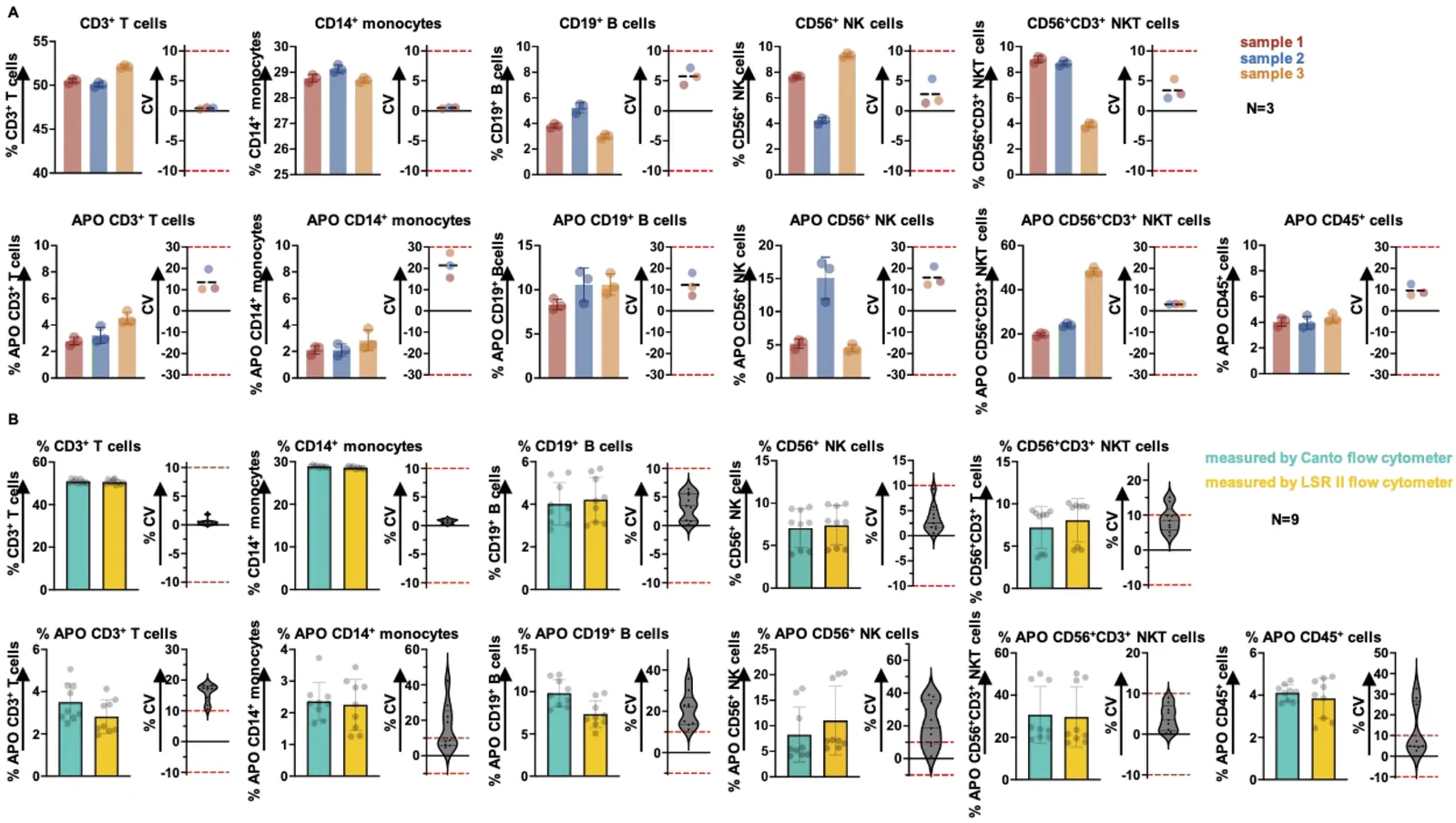
Intra-assay and inter-instrument variability. **(A)** Intra-assay precision analysis: the left panel presents the mean of replicates, where each point represents a single sample replicate and error bars indicate ± SD. The right panel shows CV values for each replicate within a sample. The dashed line represents the mean CV across three samples, and the red line indicates the acceptance criteria. (*n*-3) **(B)** Inter-instrument variability: the left panel compares cell frequencies measured by BD FACSCanto and BD LSR II. The right panel displays the CV values between two instruments, with the red line marking the 10% acceptance threshold. (*n* = 9) (data represent mean ± SD).

### Inter-instrument variability

To ensure harmonization across different flow cytometers, stained PBMCs were analyzed on two instruments: BD FACSCanto and BD LSR II. The variation in cell frequencies between instruments was assessed.

As illustrated in Figure 5B, the two instruments produced comparable results for major cell populations, with CV values below 10%, supporting instrument consistency. However, for rare events such as apoptotic cells, the CV exceeds 10%, indicating higher variability in detecting low-frequency populations across instruments.

### Inter-operator reproducibility

Due to the complexity of sample preparation and data acquisition in flow cytometry, reproducibility between different operators was evaluated (Figure 6A). The mean difference in cell frequency between operators was significantly below the 5% tolerance limit (Figure 6B). Both CV and the relative change percentage were below 10% for all cell subsets (Figure 6C). However, MFI measurements showed statistically significant differences between operators, including CD3, CD56 and CD66b. Despite these statistical differences, the CV and relative change percentage remained below the predefined 10% tolerance threshold in most cases (Figures 6D–F).

**Figure 6:**
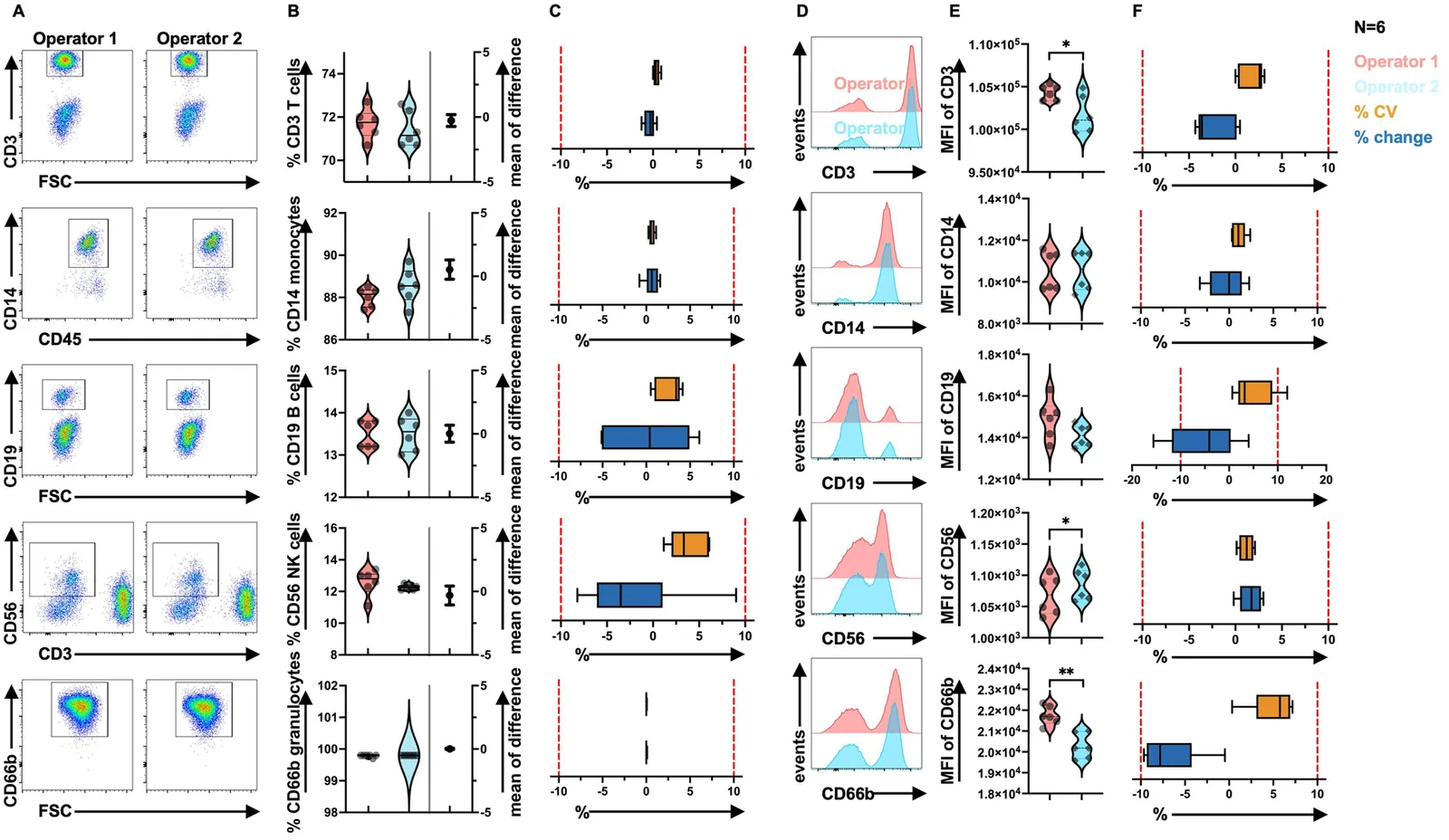
Inter-operator reproducibility. **(A)** Representative dot plots of cell populations analyzed by two operators. **(B)** Statistical analysis of mean difference in cell frequency between operators. **(C)** CV and relative change percentage of cell frequencies measured by different operators. Relative change % was calculated = (cell population by operator 2—cell population by operator 1) × 100/ (cell population by operator 1). Mean of difference was calculated as the mean of (cell population by operator 2—cell population by operator 1). **(D)** Representative histograms of MFI comparisons between operators. **(E)** Statistical analysis of MFI discrepancies between operators. **(F)** CV and relative change percentage of MFI measurements between operators. Relative change % was calculated = (MFI of cell population by operator 2—MFI of cell population by operator 1) × 100/ (MFI of cell population by operator 1). Mean of difference was calculated as the mean of (MFI of cell population by operator 2—MFI of cell population by operator 1). A two-tailed t-test was used for statistical analysis. * means *p* < 0.05. ** means *p* < 0.01. *** means *p* < 0.001. (*n* = 6, data represent mean ± SD).

### Sample stability over time

To assess the stability of stained samples over time, samples were stored for 6 h at room temperature (RT) or 24 h at 4 °C and compared with freshly stained samples (0-hour baseline). Percentage change and CV were calculated at each time point relative to the baseline. Stability was defined as percentage change and CV values remaining within ±20%.

As shown in Figure 7A, CD3+ T cells and CD56+ NK cells remained stable, with percentage change and CV values within the 20% threshold at both 6 and 24 h. However, CD19+ B cells and CD56 + CD3+ NKT cells showed a decrease after 6 h. CD14 + monocytes remained stable for 6 h but decreased after 24 h. Apoptotic cells increased over time, particularly after 24 h, in CD14 + monocytes, CD45 + cells, CD56+ NK cells, and CD56 + CD3+ NKT cells.

**Figure 7:**
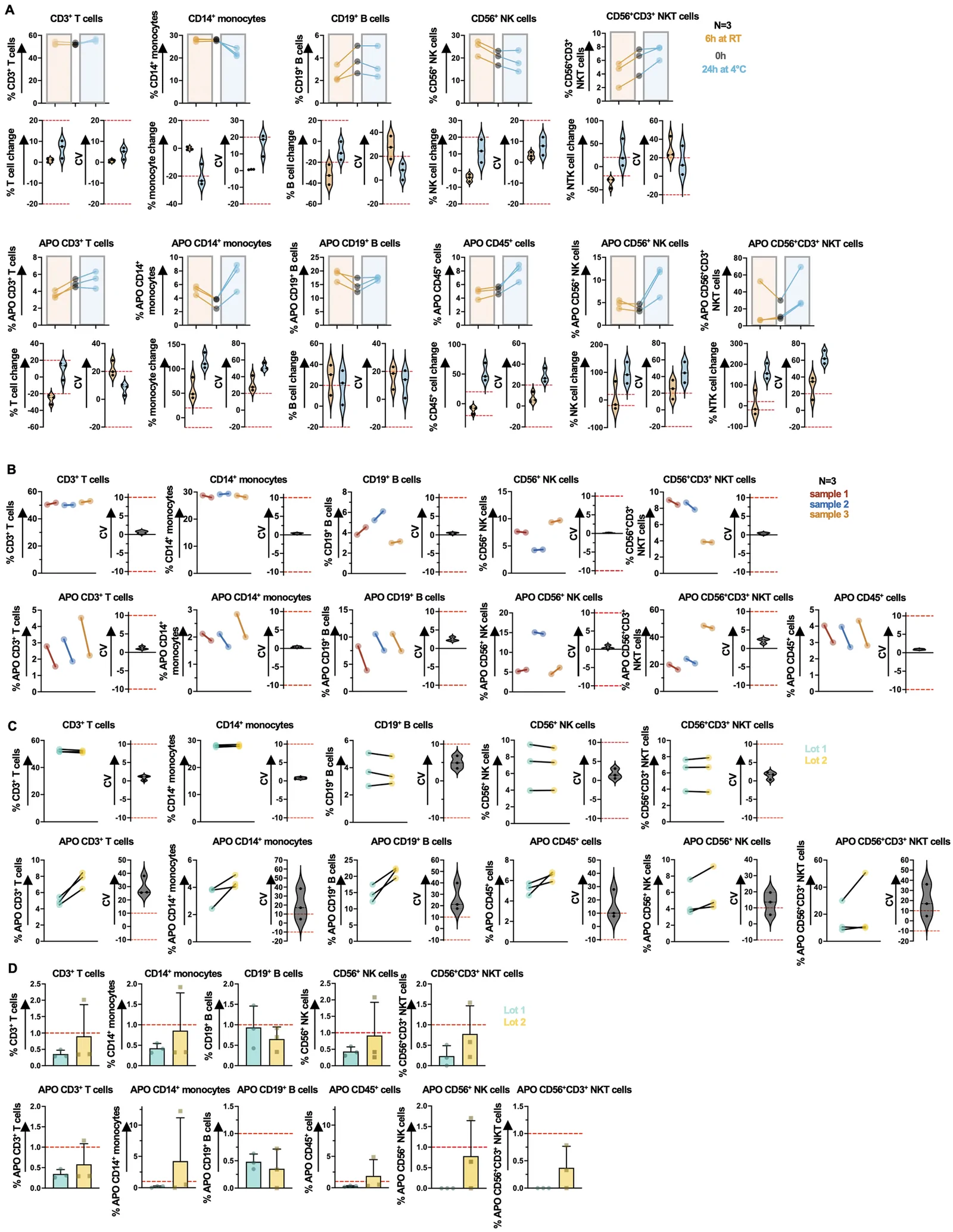
Sample stability, cocktail stability, lot-to-lot variation, and carryover. **(A)** Stability analysis of stained samples stored at 6 h (orange) and 24 h (blue), compared to freshly stained samples (grey) across different cell populations. Percentage change and CV were calculated, with the red line indicating the 20% acceptance threshold. Data from three individual donors were analyzed. **(B)** Stability of pre-mixed versus freshly prepared antibody cocktail. The red line indicates the acceptance threshold of CV < 10%. Data from three individual donors were analyzed. **(C)** Comparison of cell population percentages between Lot 1 and Lot 2 antibodies, highlighting lot-to-lot variability. **(D)** Carryover assessment using two different antibodies lots. The red line marks the 1% acceptance threshold, with higher contamination observed in Lot 2, particularly for rare events. Data from three individual donors were analyzed. Apoptotic cells were abbreviated as APO. (*n* = 3, data represent mean ± SD).

### Antibody cocktail stability

To ensure reagent consistency, a 7-color pre-mixed cocktail stored at 4 °C for 24 h was compared with a freshly prepared cocktail. Stability was assessed by measuring the CV% across different cell populations, with an acceptance criterion of CV < 10%.

As shown in Figure 7B, the pre-mixed cocktail retained optimal performance, with CV values below 10%, confirming that pre-mixing does not affect antibody stability within 24 h.

### Lot-to-lot variability

To evaluate lot-to-lot variability in antibody conjugation and staining intensities, cell percentages were compared between Lot 1 and Lot 2. Acceptable variability was defined as CV < 10% between different lots.

As shown in Figure 7C, T cells, monocytes, B cells, NK cells, and NKT cells remained within the expected range, with CV values between -10% and 10%. However, apoptotic cells (Apotracker+7AAD + and Apotracker+7AAD-) exhibited greater variability, exceeding the expected range. This discrepancy is likely due to the low percentage of apoptotic cells and non-GMP or diagnostic-grade reagents.

### Carryover evaluation

To assess instrument carryover, buffer tubes were inserted between sample tubes, and background events were analyzed using the same gating templates. The acceptance criterion was carryover below 1%.

As shown in Figure 7D, Lot 2 antibodies exceeded the 1% acceptance threshold, indicating a higher risk of carryover contamination. Carryover was especially critical for rare events, such as apoptotic CD14 + monocytes and apoptotic CD45 + cells, where contamination exceeded 5%.

### Effect of lysing on analysis

To evaluate the impact of single versus double lysing procedures on cell frequency and MFI, red blood cells (RBCs) in BD Multi-Check Control samples were lysed once or twice using a lysing solution. CV and relative changes were calculated between single-lysed and double-lysed samples to assess variability.

Although statistically significant differences (*p* < 0.05) were observed in the cell frequencies of T cells, monocytes, and NK cells between single and double lysing, the practical impact was minimal. CV and relative changes remained below the threshold of 10, which is commonly regarded as a practical threshold for low technical variability (7), indicating that the differences might be not biologically significant (Supplementary Figure S9).

MFI values were compared between single-lysed and double-lysed samples to assess potential fluorescence intensity shifts due to RBC removal. CV and relative change were minimal across most markers, except for CD14 (*p* < 0.001. Supplementary Figure S10).

### System suitability

To further validate the accuracy, robustness and reliability of the analytical method under real-world operating conditions, nine independent runs were performed over a four-month period by multiple operators using four lots of BD Multi-Check Control. Across all runs, the measured values for all five monitored leukocyte populations remained within the manufacturing-assigned, lot-specific target ranges (Supplementary Table S3). These results demonstrate consistency with the manufacturer assigned target ranges and support longitudinal assay and system performance across independent runs.

Overall, the evaludated analytical validation parameters, predefined acceptance criteria, and corresponding outcomes are summarized in Supplementary Table S4.

## Discussion

Given the complex nature of ATMPs and the challenges associated with production variability and batch-to-batch inconsistencies, regulatory agencies have established stringent guidelines for analytical method development and validation to ensure product quality, efficacy and patient safety (8–10) These guidelines emphasize fit-for-purpose approaches, ensuring that assays are tailored to the unique characteristics of each product while meeting regulatory expectations. In this context, a validated analytical method capable of accurately measuring the critical quality attributes (CQAs) is essential for ensuring product consistency and potency.

Flow cytometry, with its multiparametric capability, high sensitivity, and rapid high-throughput analysis (11), was selected to evaluate leukocyte composition and apoptotic-cell fractions as representative cellular measurands in the PBMC-based model used in this study. This study aimed to optimize flow cytometry panel and device settings, validate assay specificity, sensitivity, and reproducibility, and provide practical recommendations.

Before assay validation, designing an optimized flow cytometry panel was critical to ensure high signal resolution and minimal background interference. One fundamental principle in panel design is to match low-expression markers with high-intensity fluorochromes, improving gating efficiency and resolution (12). In this study, CD14, CD56, and CD19, markers with relatively low expression levels, were paired with high intensity fluorochromes to enhance fluorescence separation and minimal spectral overlap.

To achieve optimal panel performance, PMT voltage optimization and antibody titration were conducted. PMT voltage settings were carefully adjusted to enhance fluorescence detection while preventing excessive fluorescence spread (13). Research-grade reagents were titrated, while GMP- and diagnostics-grade reagents were used at manufacturer-recommended concentrations, as they undergo strict quality control, lot-to-lot validation, and stability testing, making additional titration unnecessary. The optimized antibody concentrations were applied in all subsequent experiments, ensuring minimal background noise, maximum signal resolution, and great separation between positive and negative populations.

To ensure panel robustness, compensation and steric hindrance were systematically evaluated (14). FMO controls confirmed that removing a specific antibody resulted in signal intensities comparable to fully stained samples, demonstrating minimal spectral spillovers. Additionally, steric hindrance assessments revealed minimal interference across most antibodies, except of 7AAD, which exhibited higher variability, likely due to low frequency of apoptotic cells. This finding suggests that higher cell acquisition thresholds should be set for rare event detection, improving data reliability.

Following the optimization of reagents, device settings and flow cytometry panel design, we proceeded with assay validation, focusing on specificity, sensitivity, and linearity to ensure robustness, reliability and reproducibility (Supplementary Figure S11).

Specificity refers to an analytical method's ability to distinguish the target analyte from non-specific signals, preventing false positive (15). Our results demonstrated high specificity, with false positive rates for CD14, CD3, CD45, and CD56 all below 1%, confirming minimal non-specific antibody binding and reinforcing the reliability of the antibody panel. However, Apotracker and 7AAD exhibited higher false positive rates, likely due to the rarity of apoptotic cells. This suggests that false positive rates increase as the target cell population decreases, emphasizing the need for higher acquisition thresholds when analyzing rare events (16).

Sensitivity in flow cytometry is critical for detecting rare events and dimly expressed antigens (17). To establish the LLOQ, a dilution-based spiking approach was employed (17). Our assay maintains high sensitivity, with precise quantification of low-abundance populations. Additionally, LOB and LOD further confirmed the assay's capability to detect and quantify rare cell populations with high precision.

Flow cytometry is inherently quasi-quantitative, as it provides relative rather than absolute measurements, due to the lack of external calibrators (18). To verify accuracy, serially diluted stained samples were compared to expected values. All tested populations met the ICH Q2(R2) criteria (8), confirming the assay's reliable quantitative performance.

A linear relationship between analyte concentration and response is essential for confirming an analytical procedure's suitability (8). In this study, linearity was evaluated by plotting measured absolute cell counts against expected values across a dilution range, with normalization using counting beads. The results demonstrated strong assay linearity, with R^2^ values exceeding 0.9 across all cell populations.

Ensuring assay reproducibility and robustness is critical for flow cytometry-based analytical methods intended for clinical and regulatory applications. This study systematically evaluated intra-assay precision, inter-instruments variability, inter-operator reproducibility, and lot-to-lot consistency to assess reliability under different conditions. In addition, BD Multi-Check Control, a commercially standardized quality control material, was used as an independent and prospective benchmark for monitoring of assay performance.

Intra-assay results demonstrated high repeatability, confirming consistent results within single runs, even for low-abundance subsets. However, higher variability was observed in NKT cells and apoptotic cells across instruments, likely due to detector sensitivity, laser alignment, and calibration settings (19). Thus, cross-platform validation is essential when switching to a backup flow cytometer due to instrument malfunctions. Inter-operator variability was minimal, with mean differences below 5% and CV < 10% for all subsets, underscoring the importance of detailed SOPs and thorough operator training. Lot-to-lot consistency was exhibited for major cell populations, but apoptotic cells showed greater variability, likely because of low abundance and the use of non-GMP and non-diagnostic-grade reagents. To mitigate reagent-related variability, appropriately qualified GMP- or diagnostic-grade reagents should be used where available. When research-grade reagents are required, each new lot should undergo predefined lot qualification or comparability testing before routine implementation.

Rare event measurements should be interpreted more cautiously than major leukocyte populations. Low frequency apoptotic populations showed greater inter-instrument and lot-to-lot variability and were more susceptible to carryover. Because rare event precision depends strongly on on the number of events acquired within the population-of-interest gate, sufficient gated-event numbers should be ensured to improve precision and reduce variability. Accordingly, population-specific sensitivity limits, adequate acquisition thresholds, and appropriate procedural controls are required for reliable rare-event quantification.

Donor-derived PBMCs are essential for assessing biological variability and real-world assay performance. However, they do not provide fixed reference values for direct evaluation of analytical accuracy. BD Multi-Check Control addressed this limitation by providing manufacturer-assigned, lot-specific target ranges established through rigorous characterization, thereby enabling assessment of method, instrument and operator performance independently of biological variability. By demonstrating that the analytical method consistently yields results within the validated ranges of stabilized controls across multiple months, operators and antibody lots, this study provides supportive evidence of accuracy for the monitored leukocyte populations relative to the manufacturer assigned target ranges, while also demonstrating longitudinal system suitability and operational consistency. This evaluation should not be interpreted as confirming analytical accuracy for all cell therapy products or matrices. Product-specific accuracy should be evaluated according to the relevant measurands and intended use.

In the context of ATMP manufacturing, where product heterogeneity is inherent, such longitudinal system suitability testing is particularly important to demonstrate that the analytical method remains in a validated and controlled state throughout clinical application. Therefore, incorporation of commercial external control should be strongly considered as a standard component of fit-for-purpose flow cytometry method validation frameworks for cell-based therapies, as it enables early detection of assay drift and provides traceable documentation for internal quality management and regulatory inspection readiness.

Beyond assay validation, practical considerations such as sample stability and reagent handling are crucial for ensuring assay reliability in routine applications. Given the complexity of flow cytometry workflows, factors such as delayed sample acquisition and antibody cocktail preparation can influence assay performance and require careful evaluation.

Our results, consistent with previous findings (20), indicate that stained samples should be acquired immediately to prevent signal degradation and population shifts, particularly for low-abundance populations. If delays are unavoidable, validated storage conditions should be implemented (3). In contrast, antibody cocktails can be pre-prepared, improving workflow efficiency and minimizing pipetting errors. However, fluorochromes interactions (21) and laboratory conditions (e.g., aliquot size, usage rate) (22) affect stability, necessitating case-by-case validation.

Other procedural factors such as instrument carryover and sample preparation methods must also be controlled. Carryover contamination, especially in low-abundance populations, can introduce artificial signal and should therefore be actively controlled. Where carryover exceeds the predefined acceptance criterion, additional instrument rinsing or cleaning cycles should be implemented and their effectiveness verified before routine sample acquisition (23). Additionally, sample preparation steps, such as lysing, can influence cell recovery and fluorescence intensity. While our results showed that single and double lysing produced minimal differences, certain markers, such as CD14, exhibited statistically significant shifts. This highlights the importance of standardizing lysing conditions to ensure consistency across experiments.

Moving beyond a one-time validation exercise, this study was designed in alignment with the analytical lifecycle principles of ICH Q2(R2) and Q14. Method performance was evaluated under conditions representative of routine use, including repeatability and intermediate precision across operators and instruments, specificity using FMO controls in donor-derived PBMC samples, dilutional linearity and population-specific sensitivity, and robustness to variations in sample storage, antibody cocktail preparation, reagent lots, carryover, and lysing procedures. Accuracy and longitudinal system performance were further supported using BD Multi-Check Control, with all five monitored leukocyte populations remaining within manufacturer-assigned, lot-specific target ranges across nine independent runs over four months. Importantly, these evaluations defined both acceptable operating conditions and limitations requiring procedural control, particularly for rare-event populations, delayed sample acquisition, and carryover. In line with an analytical lifecycle approach, initial validation confirms suitability for the intended use, while continuous performance monitoring and risk-based re-evaluation should be considered as knowledge regarding the analytical method, product, and process evolves; partial or full revalidation may be required when changes affect relevant performance characteristics. This lifecycle framework supports sustained analytical reliability, traceability, and well-founded decision-making in the areas of quality and development.

In summary, this study provides a PBMC-based proof-of-principle framework for fit-for-purpose validation of flow cytometry assays measuring major leukocyte and apoptotic-cell populations. The present data support the performance of the evaluated assay configuration under the tested PBMC-based conditions and should not be interpreted as validation of a universal cell-therapy assay. Application of the framework to a defined cellular therapy requires product-, matrix-, and analytical decision-specific definition of measurands, markers, acceptance criteria, and analytical performance requirements, followed by appropriate qualification or validation. The framework may support structured analytical development and lifecycle performance monitoring but does not by itself establish regulatory compliance, GMP/GLP suitability, or clinical applicability. Further multicenter and inter-laboratory studies are warranted to assess broader transferability and reproducibility.

## Materials and methods

### Intended analytical use and measurands

The present study used healthy-donor PBMCs as a model matrix to evaluate a proof-of-principle flow cytometry validation framework for quantification of major leukocyte populations, including T cells, B cells, monocytes, NK cells, and NKT cells, together with early and late apoptotic cell fractions in heterogeneous immune cell preparations. The assay configuration evaluated in this study was not developed as a product-specific release or in-process assay. Application of the framework to a defined cellular therapy requires product- and matrix-specific selection of measurands, markers, acceptance criteria, and analytical performance requirements according to the intended use, product characteristics, and mechanism of action.

### Samples preparation

Peripheral blood mononuclear cells (PBMCs) were isolated from healthy donor blood samples and cryopreserved in heat-inactivated fetal bovine serum (h.i. FBS) containing 10% dimethyl sulfoxide (DMSO). Cryopreserved samples were stored in liquid nitrogen until use. Before experimentation, PBMCs were thawed rapidly in a 37 °C water bath, washed, and counted to assess viability.

### Analytical method validation strategy

A fit-for-purpose method validation strategy comprising method development, analytical method validation, and lifecycle performance verification was applied (Supplementary Figure S11). Following establishment of the flow cytometry panel, optimization of antibody concentrations and PMT voltages, assessment of steric hindrance, and validation of compensation, assay performance was evaluated for specificity, sensitivity, precision, linearity, robustness, and system suitability. Predefined acceptance criteria were established based on the intended use of the assay, applicable regulatory guidance, and published recommendations for flow cytometry assay.

### Flow cytometry analysis

PBMCs were transferred into fluorescence-activated cell sorting (FACS) tubes and washed with 1 mL of FACS buffer. To minimize non-specific antibody binding, Fc receptors (FcR) were blocked using human serum for 15 min at 4 °C in the dark. Cells were incubated with pre-titrated fluorochrome-conjugated antibodies for 15 min at RT in the dark, followed by washing with 1 mL of FACS buffer. Cells were then resuspended in 500 μL of FACS buffer for acquisition.

Flow cytometry was performed using BD FACSCanto II and BD LSR II cytometers (BD Biosciences). Data acquisition was conducted using BD FACSDiva software, with compensation established using unstained and single-stained controls.

Data acquisition stopping criteria were predefined according to the objective of each experiment. For PMT voltage range and detector linearity assessment, acquisition was stop after 10,000 bead events, with 50 μL of counting beads added to each tube. For validation of the lysis procedure, acquisition was terminated after collecting 50,000 cells per sample. For antibody titration, 100,000 total cellular events were acquired per sample. For fluorescence minus one (FMO) compensation setup and steric hindrance evaluation, 5,000 events were acquired for compensation tubes and 50,000 total cellular events for experimental tubes. For quantitative assessment of assay sensitivity, specificity, lot-to-lot consistency, antibody cocktail stability, and sample dilution verification, 50 μL of counting beads were added to each FACS tube, and acquisition was automatically stopped upon reaching 5,000 bead events.

### Antibody titration

To optimize antibody staining, PBMCs were incubated with varying antibody concentrations to determine the optimal SI and the pos%. SI was calculated as follows:, where M_pos_ is the median fluorescence intensity (MFI) of the positive population, M_neg_ is the MFI of the negative population, and *σ*_neg_ is the standard deviation of the negative population.

### Determination of optimal PMT voltage

Optimal PMT voltage settings were established using eight-peak rainbow beads to measure the dynamic range and unstained CompBeads to determine background electronic noise.

Samples were analyzed across eight different voltage settings per detector, and the following parameters were calculated:

1) PMT linearity:, where M1 and M2 represent the MFI of the lower and upper bead peaks, respectively.
2) CV: calculated for the M1 bead population.
3) S/B: determined by dividing the M1 through the voltage series by the background MFI of the unstained CompBeads.

Additionally, lymphocytes stained with the Human CD4 Fluorochrome Evaluation Kit were tested at different voltages, and the SI was calculated at each setting to optimize fluorescence signal resolution.

### Assay sensitivity

Assay sensitivity was determined by preparing serial dilutions of stained samples spiked into unstained samples. Before preparation of the dilution series, cell concentrations were determined by manual counting and independently confirmed using a GMP-qualified LUNA automated cell counter (Logos Biosystems). Consistency between the two counting methods was considered acceptable when the CV was < 10%. Following confirmation of cell concentration, the serial dilution series was prepared according to the predefined experimental design.

LOB, LOD, and lower limit of quantification (LLOQ) were calculated as follows:

LOB = Mean_blank_ + 1.645 × SD_blank_

LOD = LOB + 1.645 × SD_near−blank_, near-blank means spiked low fraction of stained samples

LLOQ was defined as the lowest stained sample concentration where the CV is less than 30%. For rare-event measurements, the CV < 30% criterion was predefined based on published recommendations for high-sensitivity flow cytometric assays. In addition to precision, the number of events detected within the population-of-interest gate was considered when interpreting measurements near the LLOQ.

### System suitability

To assess longitudinal assay performance, encompassing the flow cytometry panel, antibodies, staining procedure and operator proficiency, as well as to ensure instrument consistency in terms of setup and optical performance, the BD Multi-Check Control (BD Biosciences) was used as an external quality control. This control consists of stabilized human whole blood with manufacturer-assigned lot-specific target values (TV) and acceptable ranges for defined cell populations, providing a standardized, biologically relevant benchmark across independent experimental runs. Control samples were processed according to the manufacturer's instructions and stained with the optimized flow cytomtry panels following the established staining standard operating procedures (SOPs). Measurements acquired across different dates, operators, and reagent lots were compared against the corresponding lot-specific target ranges. Assay performance was considered acceptable when the measured values (MV) remained within the manufacturer-defined target ranges.

### Data analysis

Flow cytometry data were analyzed using FlowJo software (version 10.8.1, BD Biosciences). The gating strategy is illustrated in Supplementary Figure S12. Representative original FACS dot plots have been provided in Supplementary Figure S13. Microsoft Excel (Microsoft, Redmond, WA, USA) and Prism 9 (San Diego, CA, USA) were used to perform statistical analysis. For paired comparisons, parametric tests were applied when the distribution of paired differences was considered approximately normal. Otherwise, Wilcoxon signed-rank tests were used. For datasets with very small numbers of independent observations, non-parametric tests were preferred as formal tests for normal distribution have limited power. *p* values < 0.05 were considered statistically significant. No adjustment for multiple comparisons was applied because hypothesis testing was exploratory and supportive. Analytical validation decisions were primarily based on predefined acceptance criteria. Results are reported as mean ± SD unless otherwise specified. Figures and tables were generated using Excel, PowerPoint (Microsoft, Redmond, WA, USA), Prism 9, RStudio (version 4.1.2; RStudio, Boston, MA, USA) and FlowJo.

## Data Availability

The original contributions presented in the study are included in the article/Supplementary Material, further inquiries can be directed to the corresponding author.
