# Supplementary material for: A PBMC-based proof-of-principle framework for fit-for-purpose validation of flow cytometry assays in cell therapy development
Source: Front Med Technol. 2026 Oct 14;8:1905186. doi: 10.3389/fmedt.2026.1905186 (PMC13713653; doi:10.3389/fmedt.2026.1905186)
Supplement: Supplementary file 1 [file Datasheet1.pdf]

Supplemental Material

Figure S1

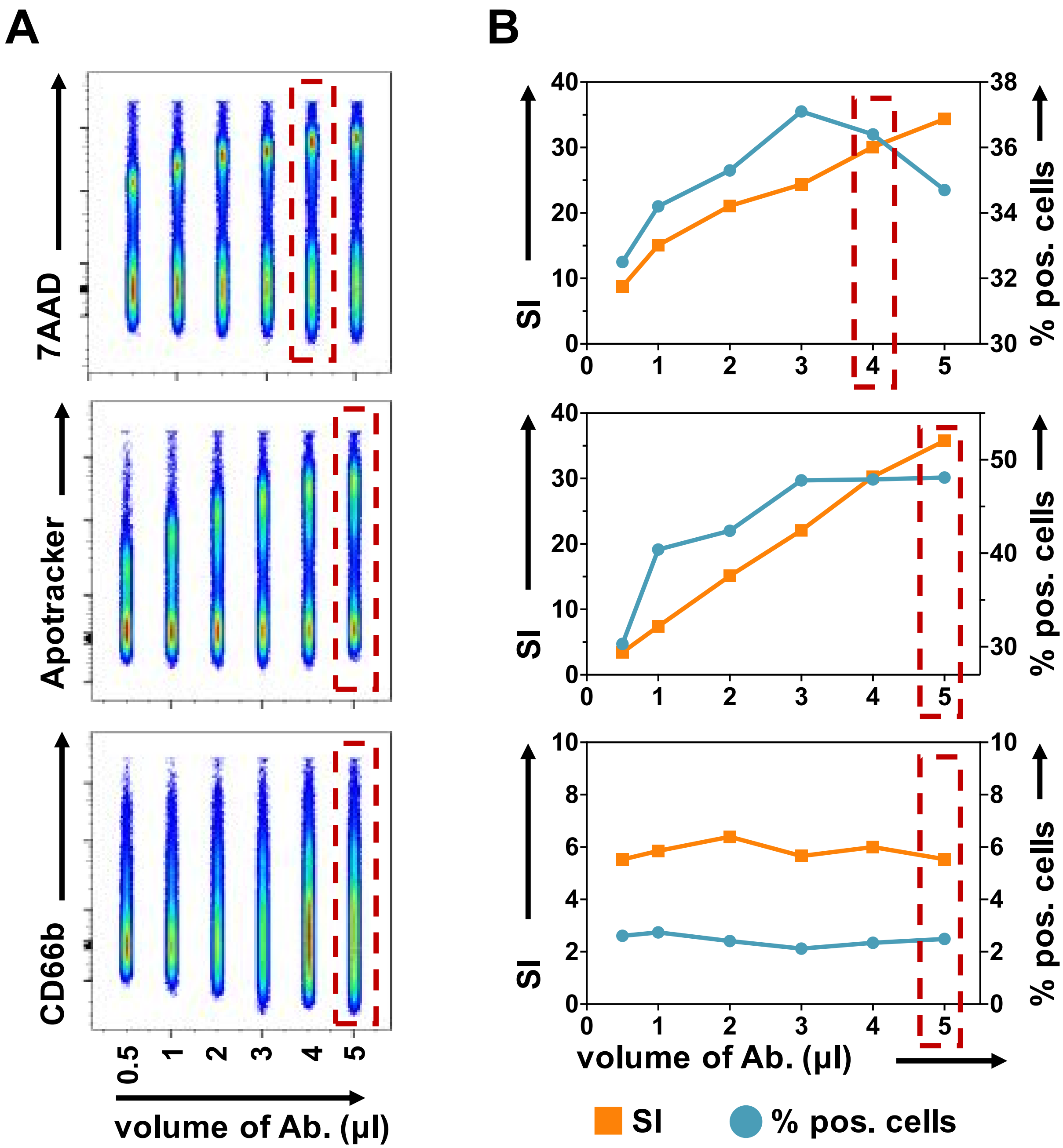

A
B
7AAD
Apotracker
CD66b
0.5
1
2
3
4
5
volume of Ab. (µl)
SI
% pos. cells
0
10
20
30
40
32
34
36
38
40
50
2
4
6
8

Figure S1. Antibody titration using staining index (SI) and percentage of positive cells (% pos.)

(A) Representative flow cytometry plots illustrating antibody titration across six different volumes (0.5 μL, 1 μL, 2 μL, 3 μL, 4 μL, and 5 μL), combined into a single concatenated plot. (B) Titration curves displaying SI and % pos. across antibody volumes (0.5 μL to 5 μL). Red rectangles indicate the identified optimal titer.

Figure S2

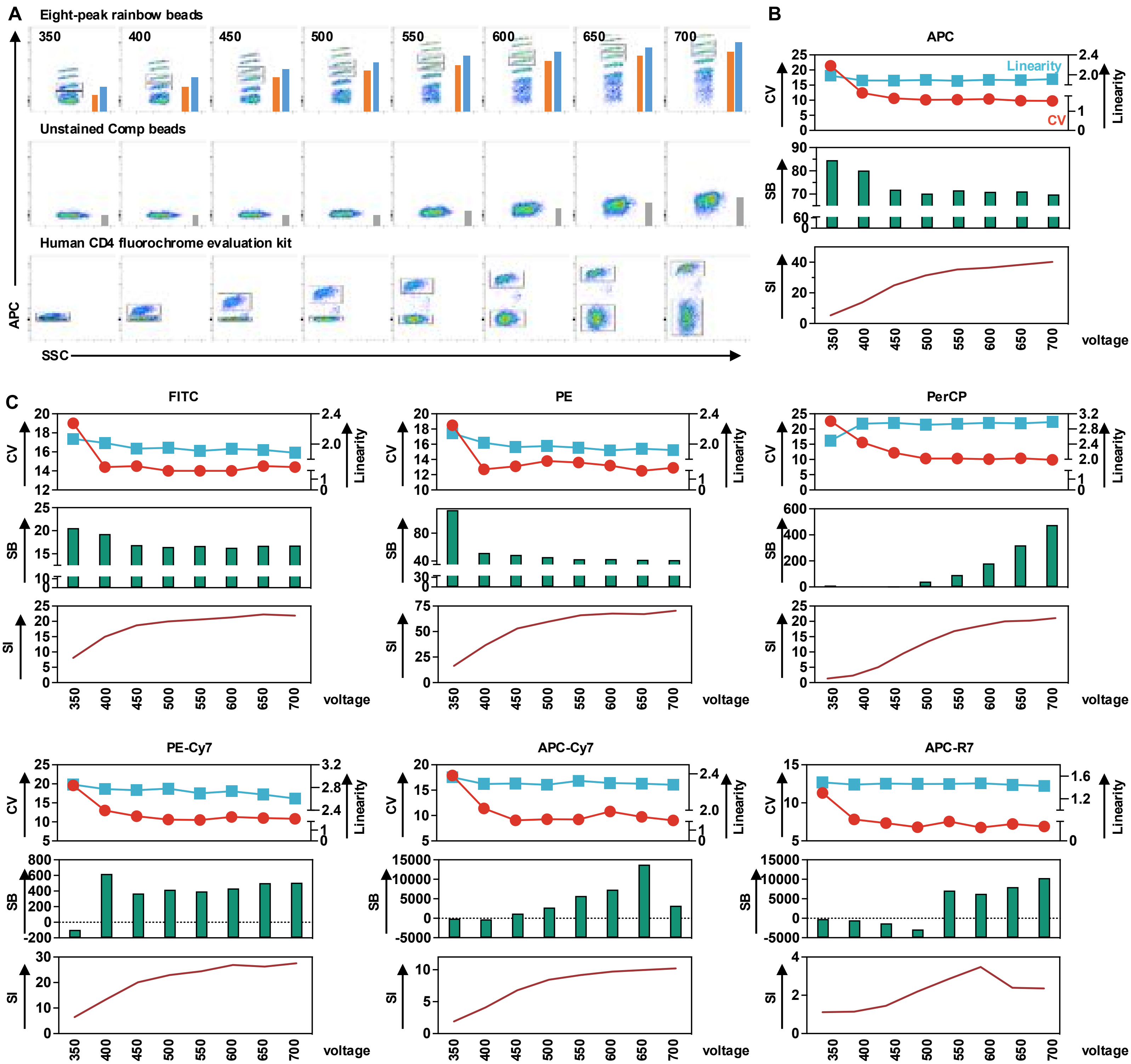

A
Eight-peak rainbow beads
350
400
450
500
550
600
650
700
Unstained Comp beads
Human CD4 fluorochrome evaluation kit
APC
SSC
B
APC
Linearity
CV
SB
SI
voltage
C
FITC
PE
PerCP
PE-Cy7
APC-Cy7
APC-R7

Figure S2. Determination of the optimal PMT voltage

(A) Dot plots showing eight-peak rainbow beads and unstained CompBeads across voltages in the APC channel (upper and middle panels). Two reference peaks (M1 and M2, indicated by rectangular gates) were selected for analysis. The same tube of lymphocytes stained with APC was analyzed at eight different voltages (lower panel). (B-C) CV, linearity, SB and SI were calculated for each voltage setting. The PMT was considered linear when the slope of the linearity curve was zero.

Figure S3

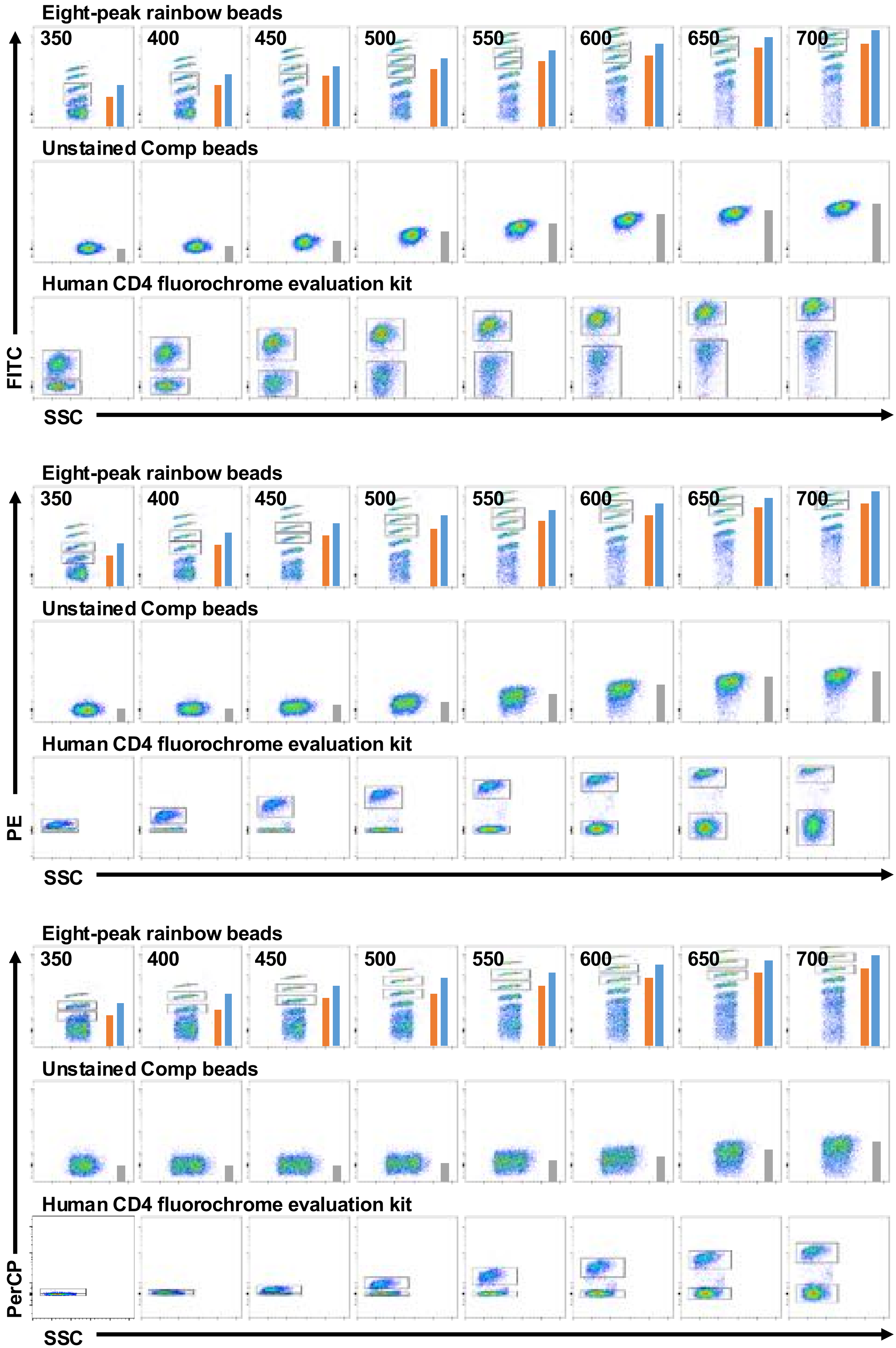

Eight-peak rainbow beads
350
400
450
500
550
600
650
700
Unstained Comp beads
Human CD4 fluorochrome evaluation kit
FITC
SSC
Eight-peak rainbow beads
350
400
450
500
550
600
650
700
Unstained Comp beads
Human CD4 fluorochrome evaluation kit
PE
SSC
Eight-peak rainbow beads
350
400
450
500
550
600
650
700
Unstained Comp beads
Human CD4 fluorochrome evaluation kit
PerCP
SSC

Figure S3 Continued

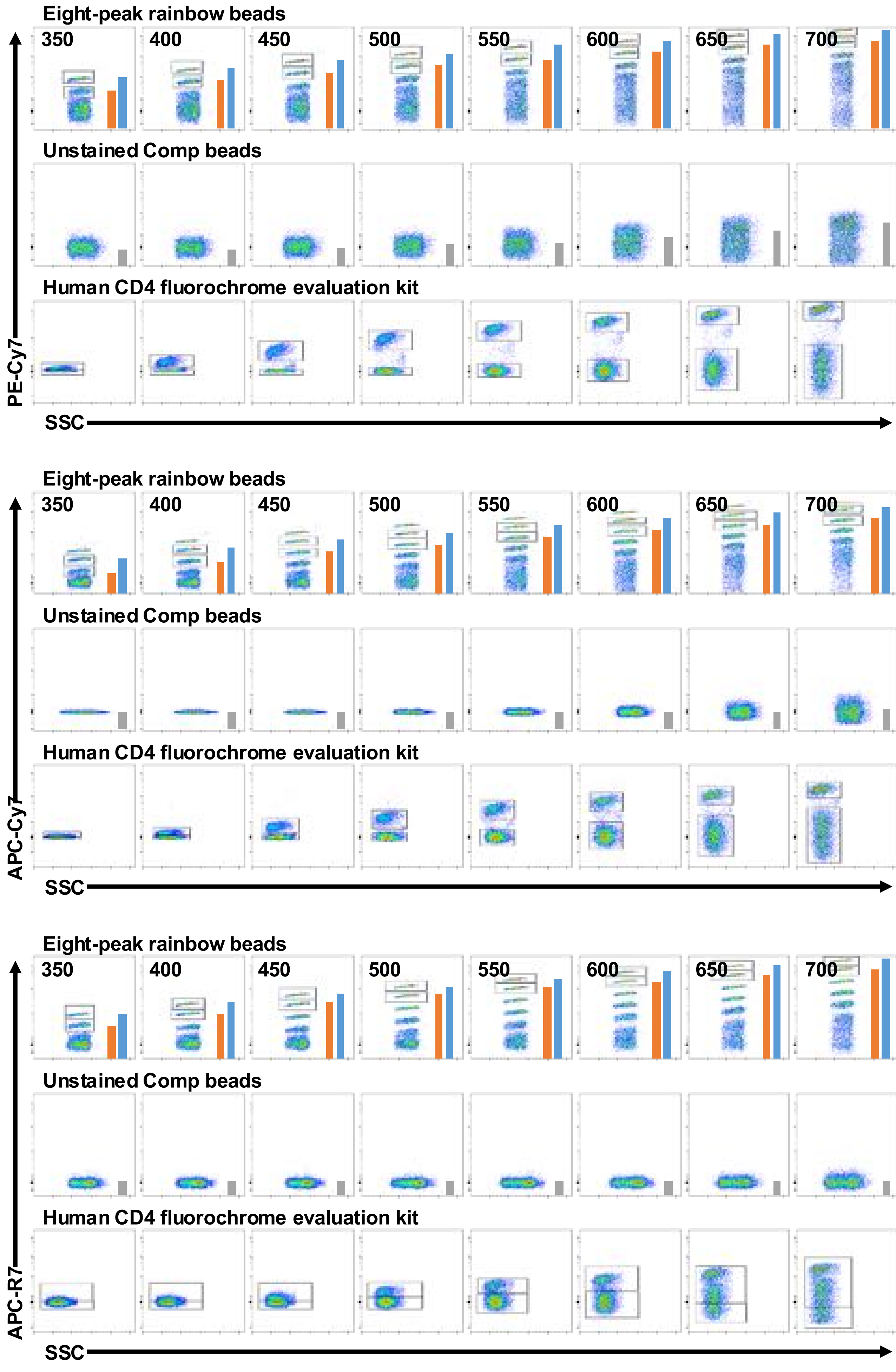

Figure S3. Representative dot plots for PMT voltage optimization across all fluorochrome channels

Figure S4

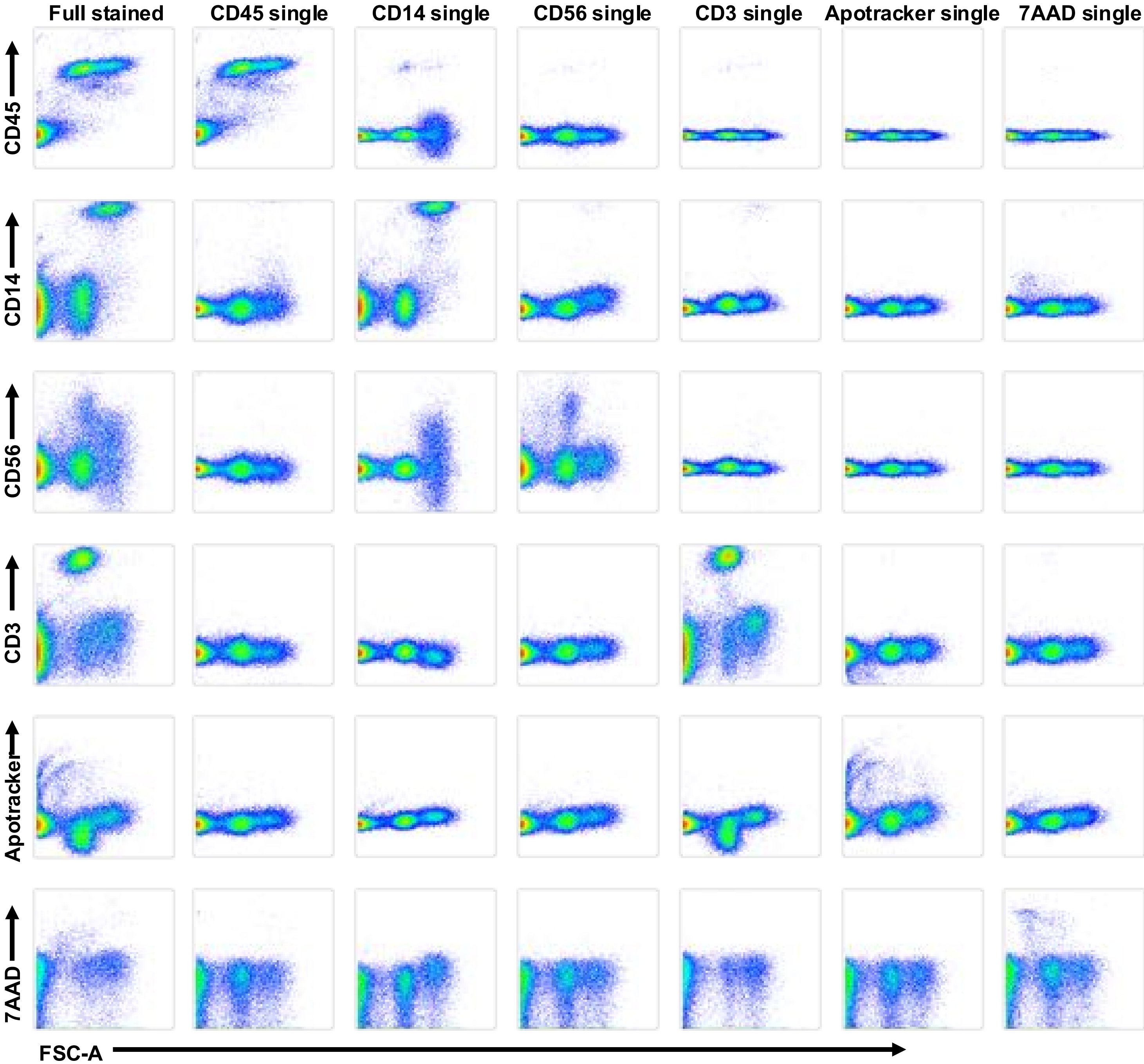

Full stained
CD45 single
CD14 single
CD56 single
CD3 single
Apotracker single
7AAD single
CD45
CD14
CD56
CD3
Apotracker
7AAD
FSC-A

Figure S4. Identification of steric hindrance in panel 1

Comparison of fully stained versus single-antibody-stained samples. FSC-A (x-axis) plotted against all antibodies (y-axis) to detect steric hindrance.

Figure S5

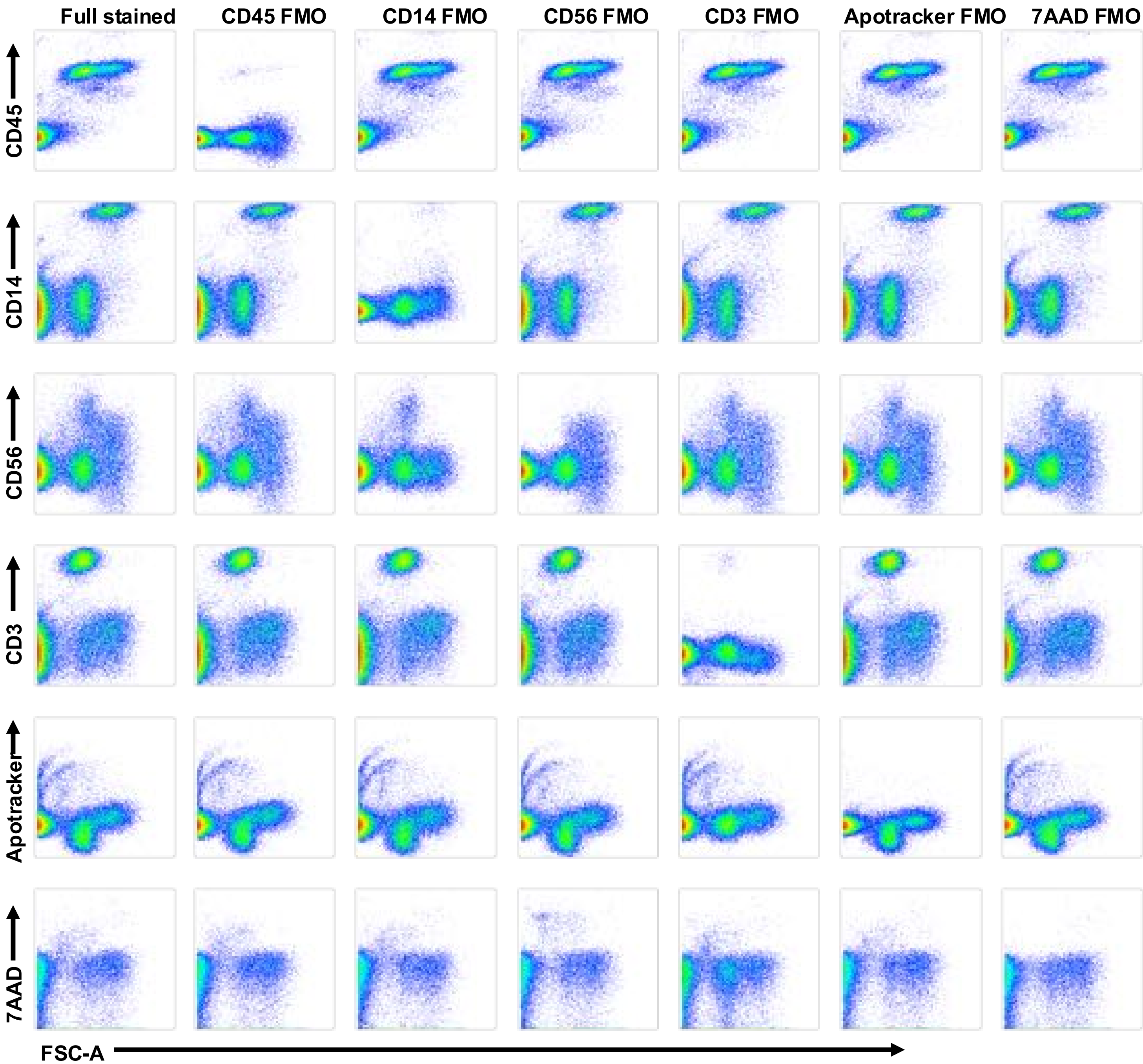

Full stained
CD45 FMO
CD14 FMO
CD56 FMO
CD3 FMO
Apotracker FMO
7AAD FMO
CD45
CD14
CD56
CD3
Apotracker
7AAD
FSC-A

Figure S5. Spreading error analysis in panel 1

Comparison of fully stained versus FMO controls. FSC-A (x-axis) plotted against all antibodies (y-axis) to evaluate spillover effects.

Figure S6

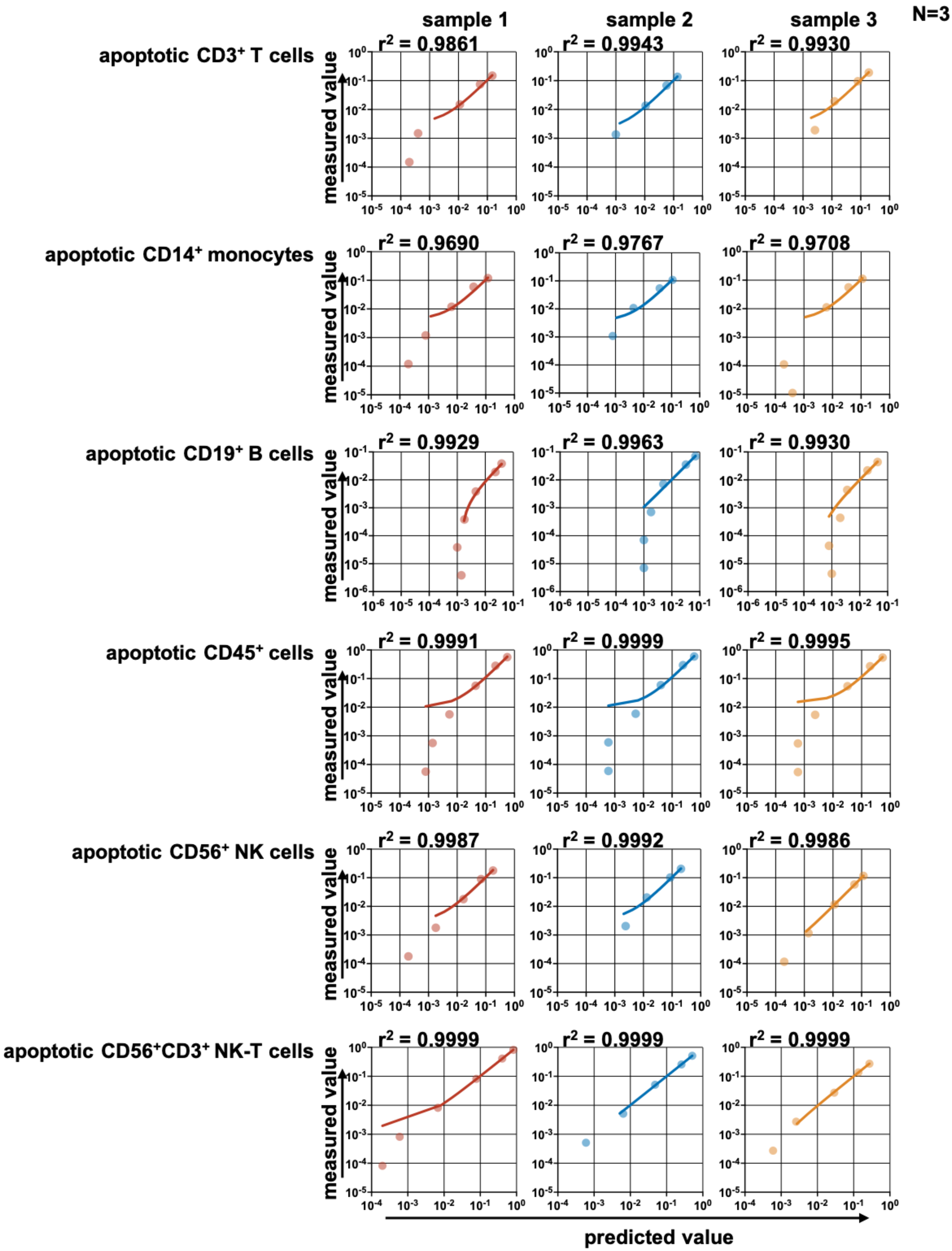
N=3
sample 1
sample 2
sample 3
apoptotic CD3+ T cells
r2 = 0.9861
r2 = 0.9943
r2 = 0.9930
apoptotic CD14+ monocytes
r2 = 0.9690
r2 = 0.9767
r2 = 0.9708
apoptotic CD19+ B cells
r2 = 0.9929
r2 = 0.9963
r2 = 0.9930
apoptotic CD45+ cells
r2 = 0.9991
r2 = 0.9999
r2 = 0.9995
apoptotic CD56+ NK cells
r2 = 0.9987
r2 = 0.9992
r2 = 0.9986
apoptotic CD56+CD3+ NK-T cells
r2 = 0.9999
r2 = 0.9999
r2 = 0.9999
measured value
predicted value

Figure S6. Linearity of apoptotic cells

The measured values of apoptotic cells were plotted against expected values using logarithmic scales, and linear regression analysis was performed for each apoptotic cell population. Data were obtained from three independent samples. (n=3)

Figure S7

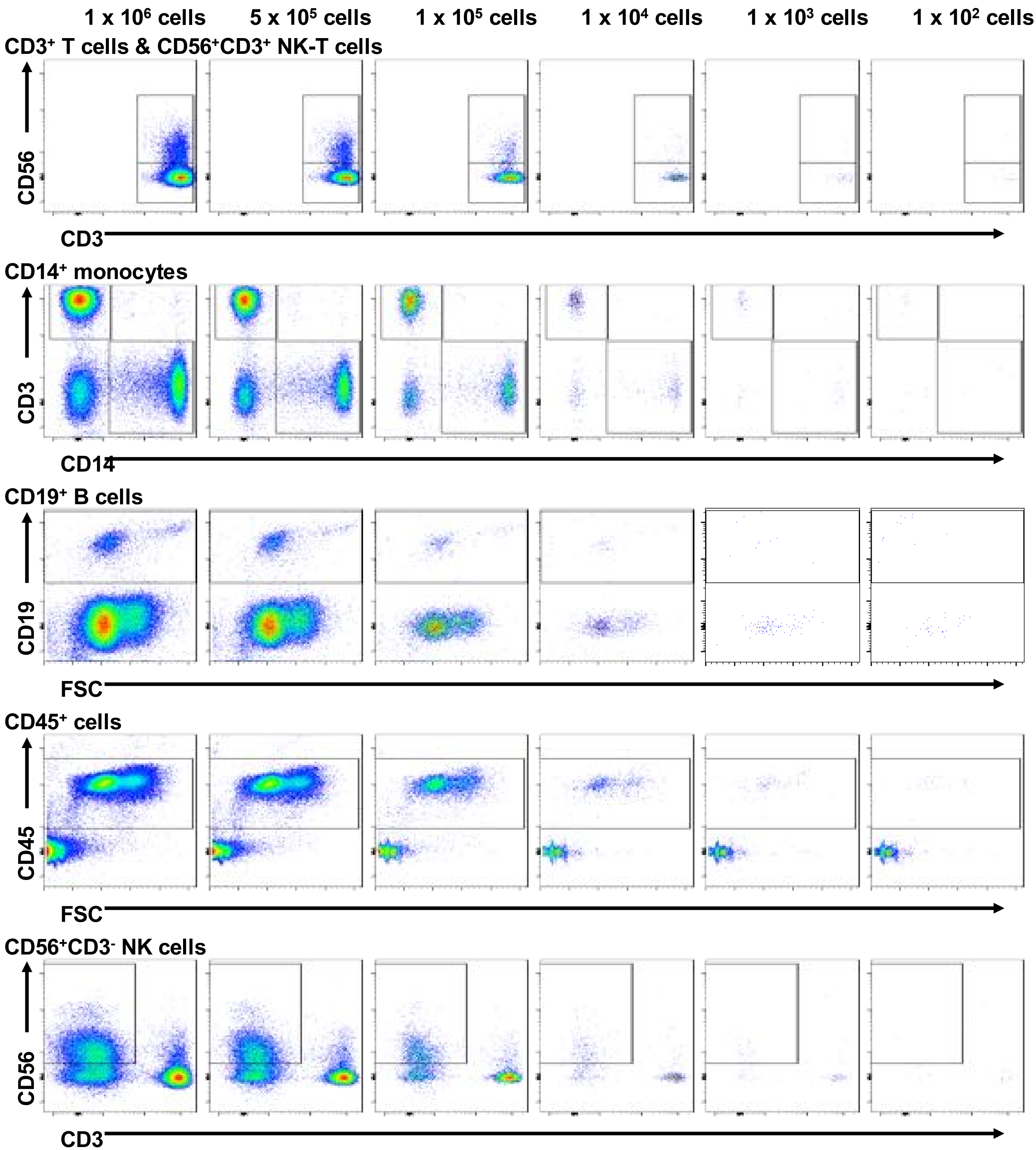
1 x 10^6 cells
5 x 10^5 cells
1 x 10^5 cells
1 x 10^4 cells
1 x 10^3 cells
1 x 10^2 cells
CD3+ T cells & CD56+CD3+ NK-T cells
CD56
CD3
CD14+ monocytes
CD3
CD14
CD19+ B cells
CD19
FSC
CD45+ cells
CD45
FSC
CD56+CD3- NK cells
CD56
CD3

Figure S7. Representative dot plots of different cell populations

Representative flow cytometry dot plots of different cell populations across dilution series.

Figure S8

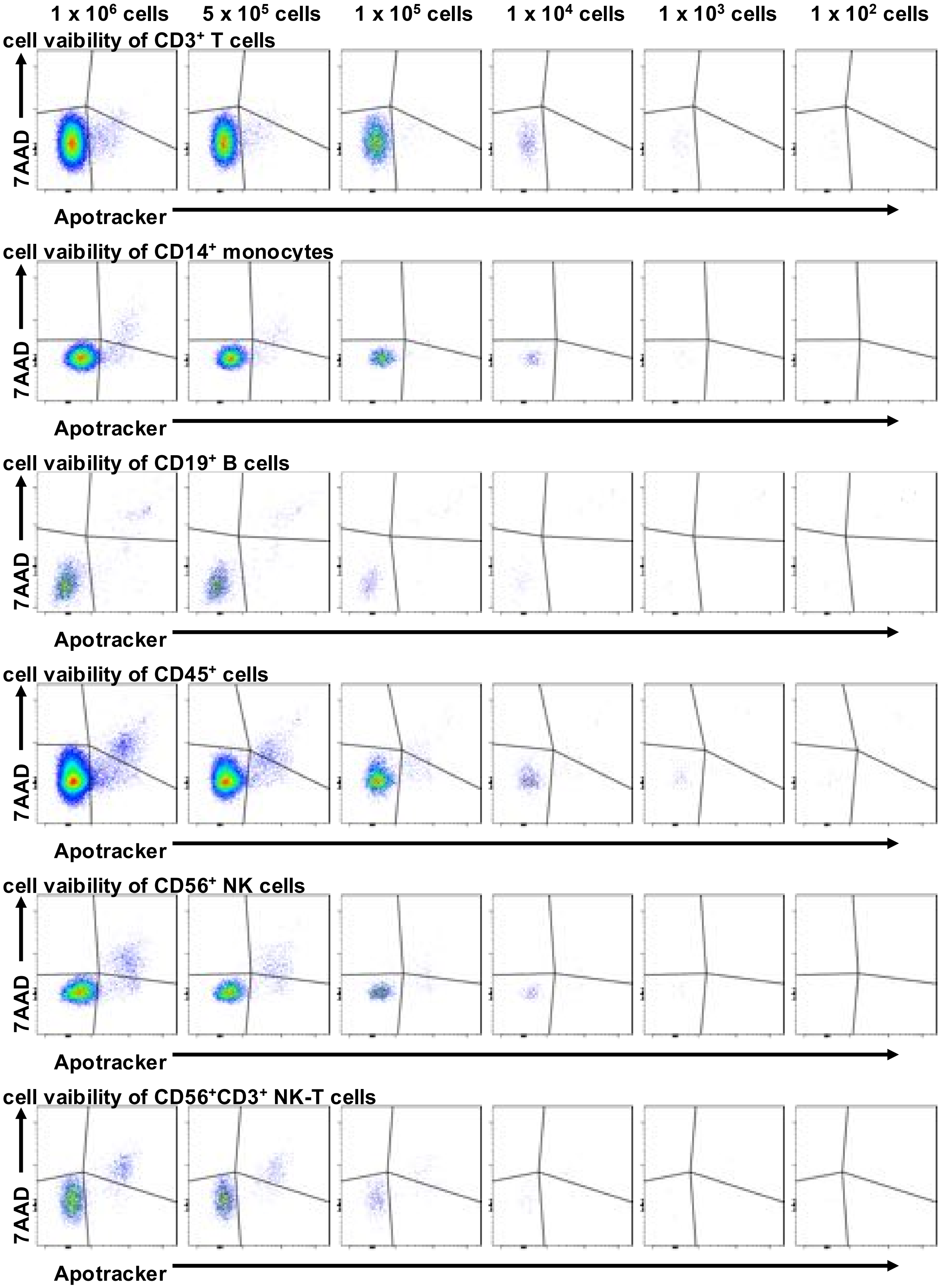

1 x 10^6 cells
5 x 10^5 cells
1 x 10^5 cells
1 x 10^4 cells
1 x 10^3 cells
1 x 10^2 cells
cell vaibility of CD3+ T cells
7AAD
Apotracker
cell vaibility of CD14+ monocytes
7AAD
Apotracker
cell vaibility of CD19+ B cells
7AAD
Apotracker
cell vaibility of CD45+ cells
7AAD
Apotracker
cell vaibility of CD56+ NK cells
7AAD
Apotracker
cell vaibility of CD56+CD3+ NK-T cells
7AAD
Apotracker

Figure S8. Representative dot plots of apoptotic cells

Representative flow cytometry dot plots of apoptotic cells across dilution series.

Figure S9

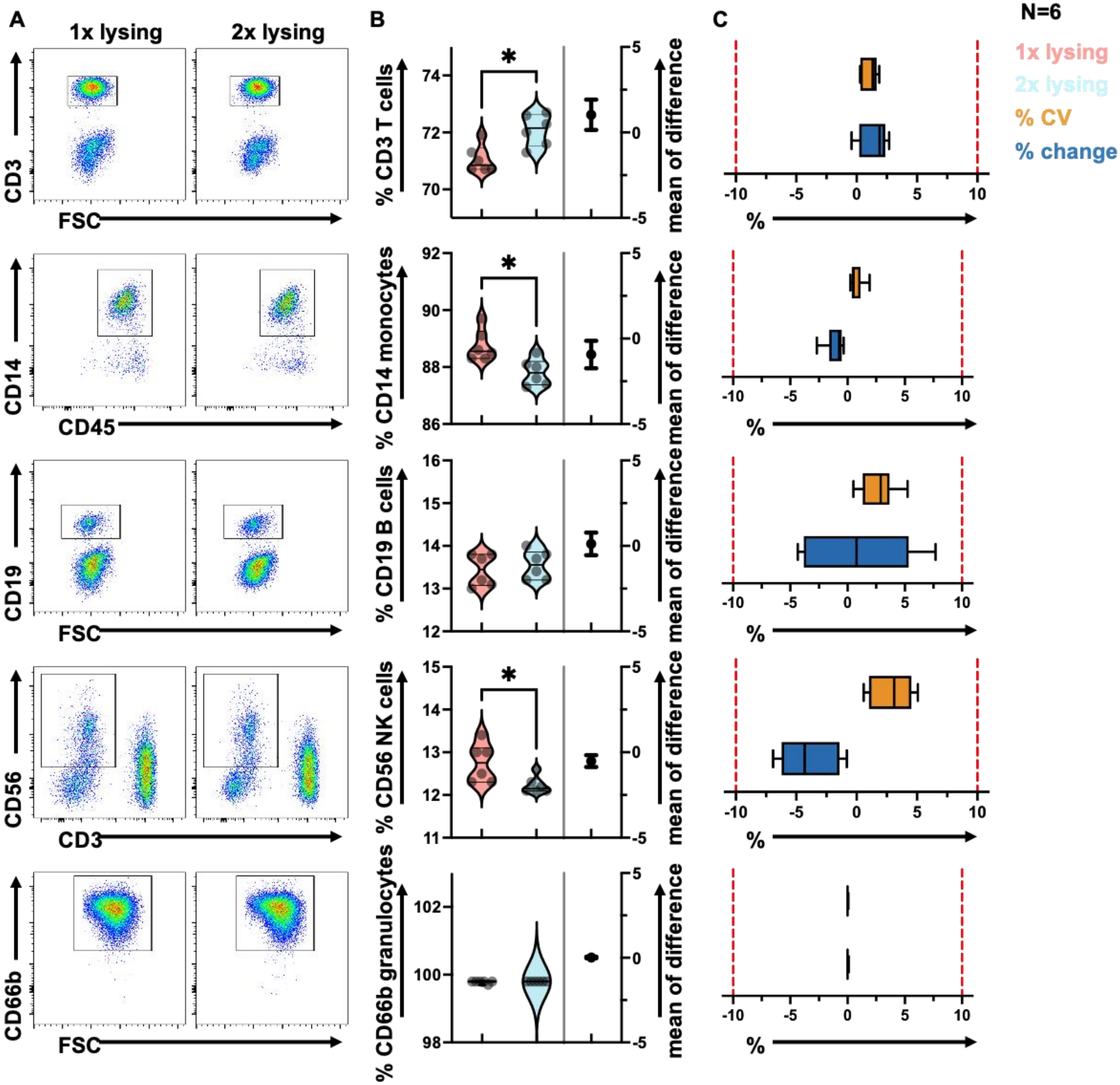

A
1x lysing
2x lysing
CD3
FSC
CD14
CD45
CD19
FSC
CD56
CD3
CD66b
FSC
B
*
% CD3 T cells
mean of difference
% CD14 monocytes
% CD19 B cells
% CD56 NK cells
% CD66b granulocytes
C
%
N=6
1x lysing
2x lysing
% CV
% change

Figure S9. Cell frequency comparison between single and double lysing

(A) Representative dot plots of cell populations after one-time versus two-time lysing. (B) Statistical analysis and mean difference of cell frequency after one-time versus two-time lysing. (C) CV and relative change in cell frequency. Relative change % was calculated = (cell population after two-time lysing – cell population after one-time lysing) x 100/ (cell population after one-time lysing). Mean difference was calculated as the mean of (cell population after two-time lysing – cell population after one-time lysing). A two-tailed t-test was used for statistical analysis. * means $p < 0.05$. ** means $p < 0.01$. *** means $p < 0.001$. n=6, data represent mean ± SD.

Figure S10

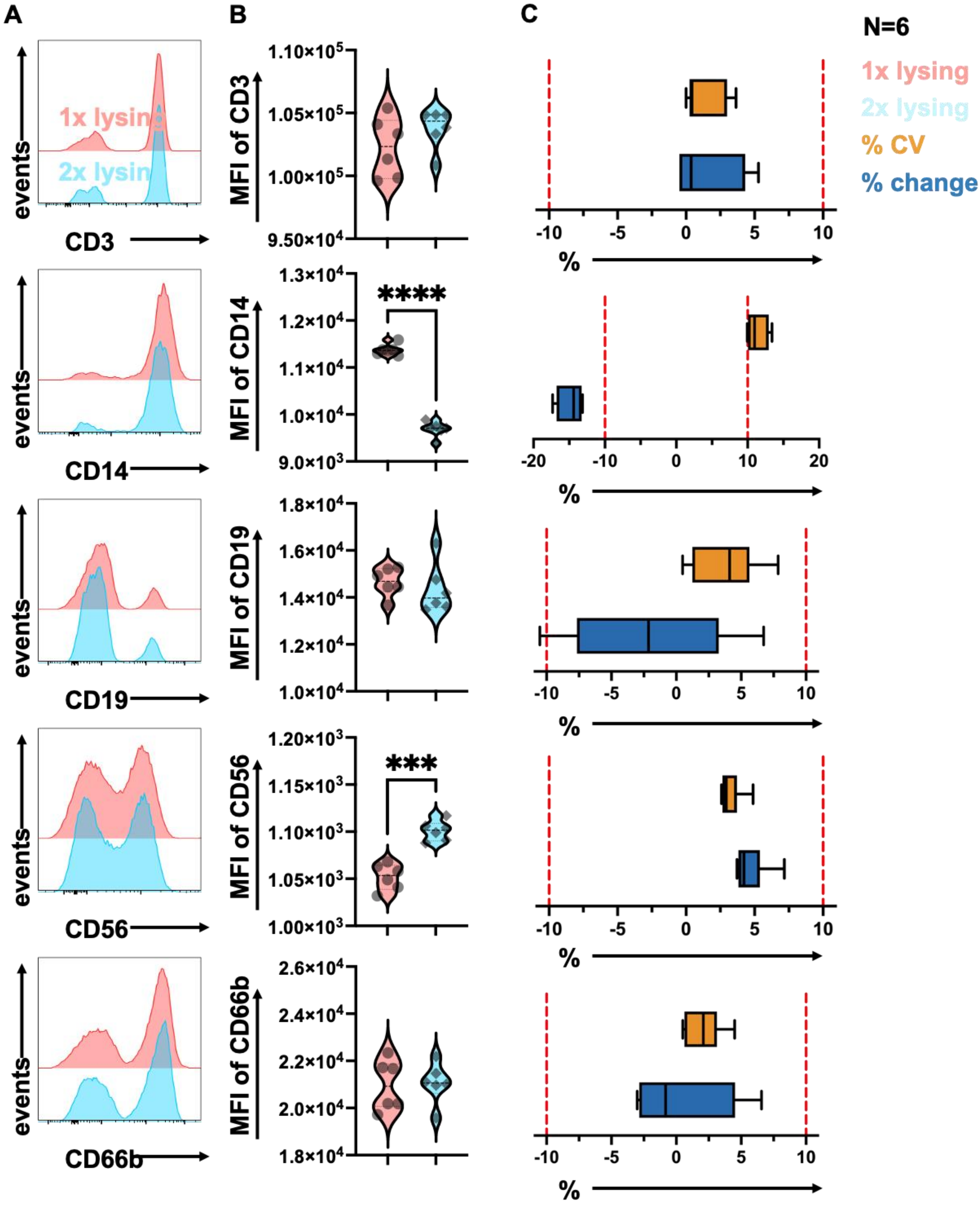

A
B
C
N=6
1x lysing
2x lysing
% CV
% change
events
CD3
CD14
CD19
CD56
CD66b
MFI of CD3
MFI of CD14
MFI of CD19
MFI of CD56
MFI of CD66b
****
***
%

Figure S10. MFI comparison between single and double lysing

(A) Representative histogram of MFI after one-time versus two-time lysing. (B) Statistical analysis of MFI differences after lysing. (C) CV and relative change in MFI across markers. Relative change was calculated = (MFI of cell population after two-time lysing – MFI of cell population after one-time lysing) x 100/ (MFI of cell population after one-time lysing). Mean difference was calculated as the mean of (MFI of cell population after two-time lysing – MFI of cell population after one-time lysing). A two-tailed t-test was used for statistical analysis. * means $p < 0.05$. ** means $p < 0.01$. *** means $p < 0.001$. n=6, data represent mean ± SD.

Figure S11

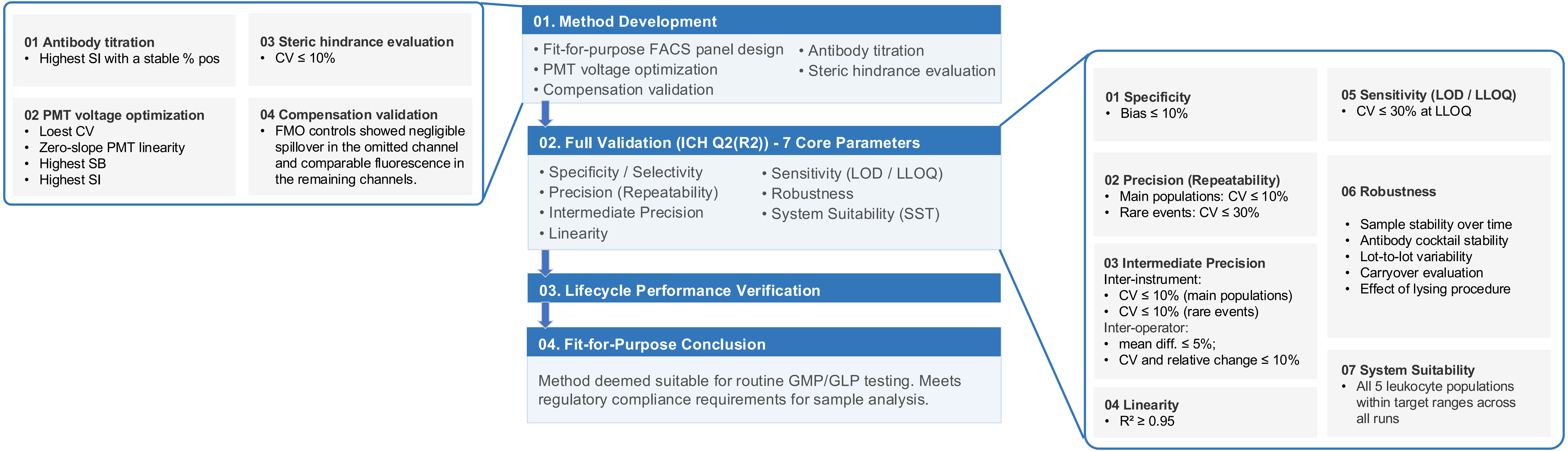
01 Antibody titration
• Highest SI with a stable % pos
03 Steric hindrance evaluation
• CV ≤ 10%
02 PMT voltage optimization
• Loest CV
• Zero-slope PMT linearity
• Highest SB
• Highest SI
04 Compensation validation
• FMO controls showed negligible spillover in the omitted channel and comparable fluorescence in the remaining channels.
01. Method Development
• Fit-for-purpose FACS panel design
• PMT voltage optimization
• Compensation validation
• Antibody titration
• Steric hindrance evaluation
02. Full Validation (ICH Q2(R2)) - 7 Core Parameters
• Specificity / Selectivity
• Precision (Repeatability)
• Intermediate Precision
• Linearity
• Sensitivity (LOD / LLOQ)
• Robustness
• System Suitability (SST)
03. Lifecycle Performance Verification
04. Fit-for-Purpose Conclusion
Method deemed suitable for routine GMP/GLP testing. Meets regulatory compliance requirements for sample analysis.
01 Specificity
• Bias ≤ 10%
05 Sensitivity (LOD / LLOQ)
• CV ≤ 30% at LLOQ
02 Precision (Repeatability)
• Main populations: CV ≤ 10%
• Rare events: CV ≤ 30%
06 Robustness
• Sample stability over time
• Antibody cocktail stability
• Lot-to-lot variability
• Carryover evaluation
• Effect of lysing procedure
03 Intermediate Precision
Inter-instrument:
• CV ≤ 10% (main populations)
• CV ≤ 10% (rare events)
Inter-operator:
• mean diff. ≤ 5%;
• CV and relative change ≤ 10%
04 Linearity
• R² ≥ 0.95
07 System Suitability
• All 5 leukocyte populations within target ranges across all runs

Figure S11. Schematic of flow cytometry assay development and ICH Q2(R2)-based full-validation strategy.

This figure summarizes assay-development procedures, seven core ICH Q2(R2) analytical-validation parameters with predefined acceptance criteria, lifecycle-performance verification items, and the fit-for-purpose conclusion. Development steps include FACS panel design, PMT optimization, compensation validation, antibody titration, and steric-hindrance assessment. Validated parameters contain specificity, precision (repeatability and intermediate precision), linearity, sensitivity (LOD/LLOQ), robustness, and system suitability testing (SST). Lifecycle assessments cover sample stability, antibody-cocktail stability, lot-to-lot variation, carryover, and lysing-procedure effects. The assay is considered fit-for-purpose for routine GMP/GLP-regulated testing and meets regulatory-compliance demands.

Figure S12

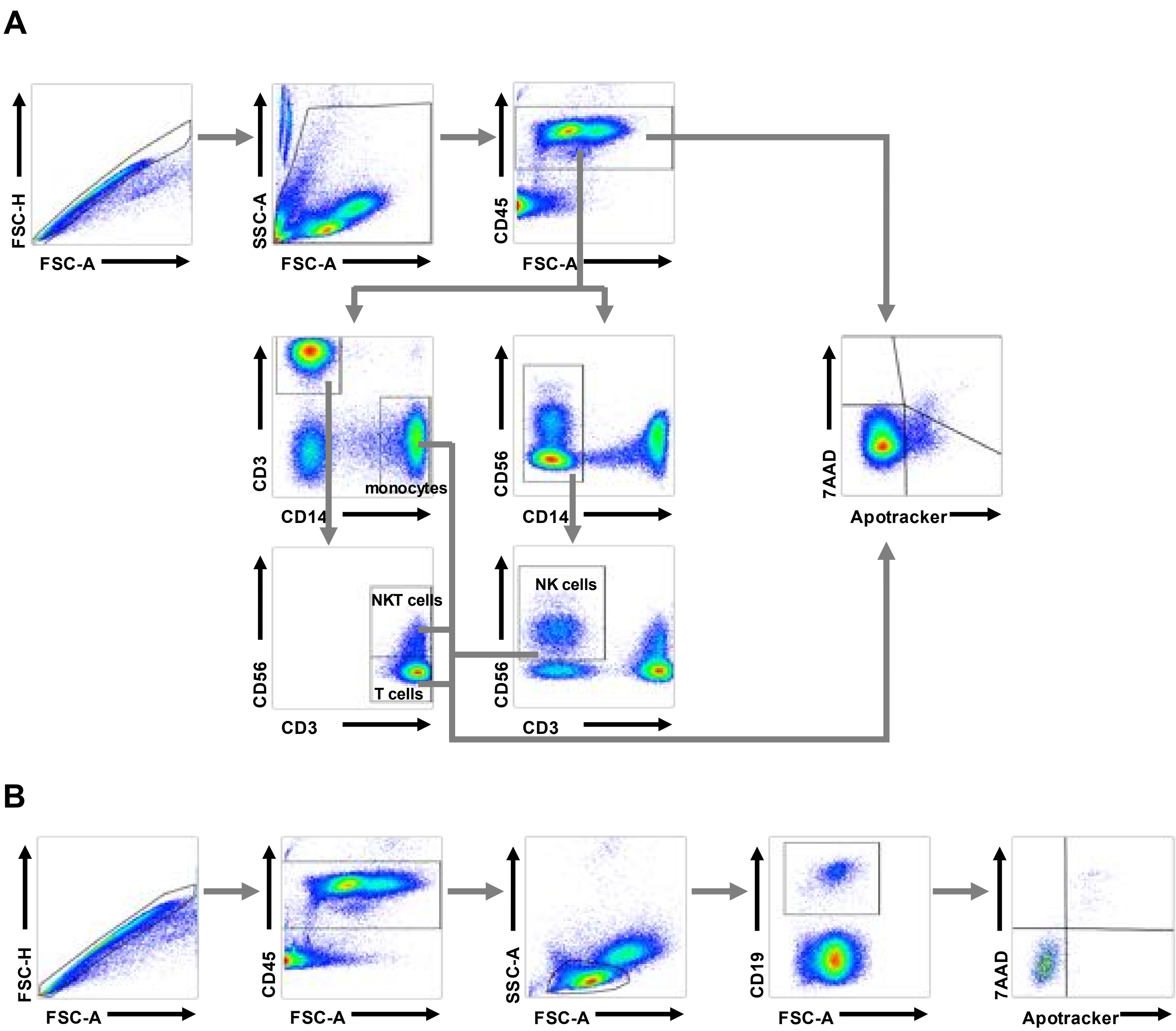
A
FSC-H
FSC-A
SSC-A
FSC-A
CD45
FSC-A
CD3
CD14
monocytes
CD56
CD14
7AAD
Apotracker
CD56
CD3
NKT cells
T cells
CD56
CD3
NK cells
B
FSC-H
FSC-A
CD45
FSC-A
SSC-A
FSC-A
CD19
FSC-A
7AAD
Apotracker

Figure S12. Gating strategy

(A) Gating strategy for panel 1. (B) Gating strategy for panel 2.

Figure S13

Specificity

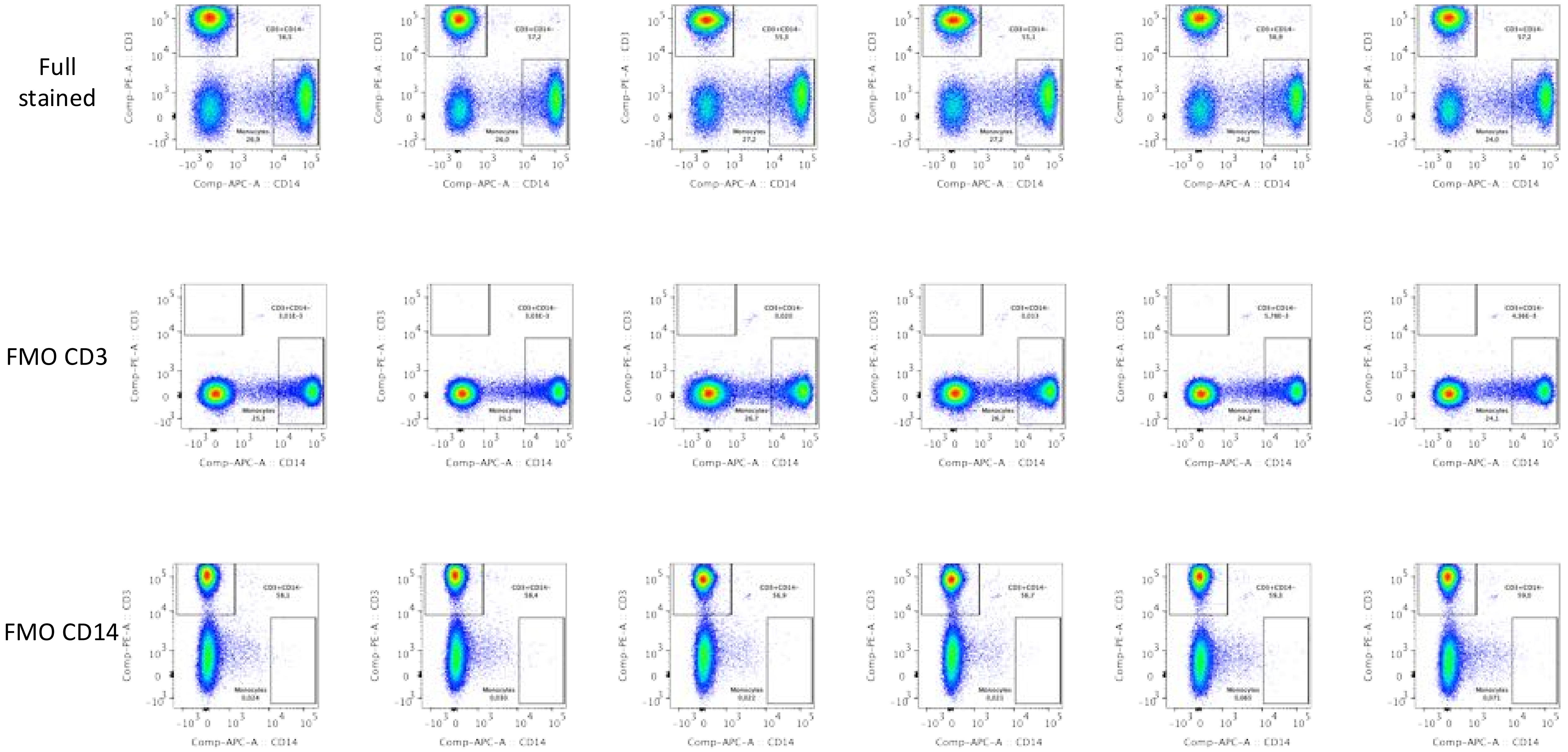

Specificity

Full stained

FMO CD19

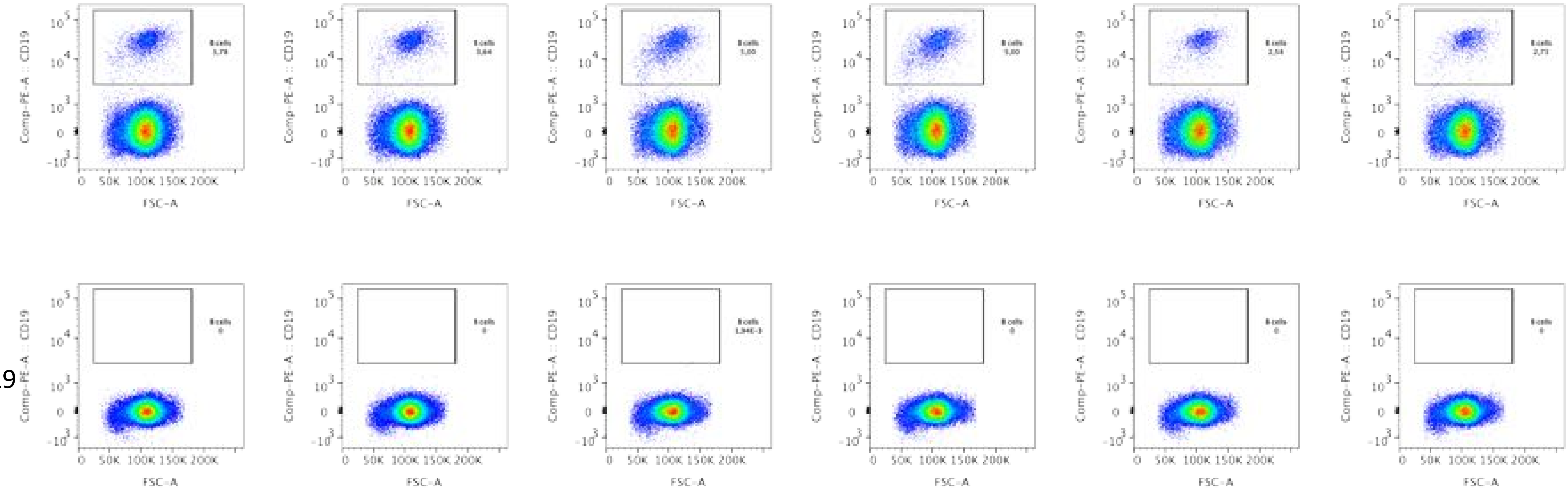

Specificity

Full stained

FMO CD45

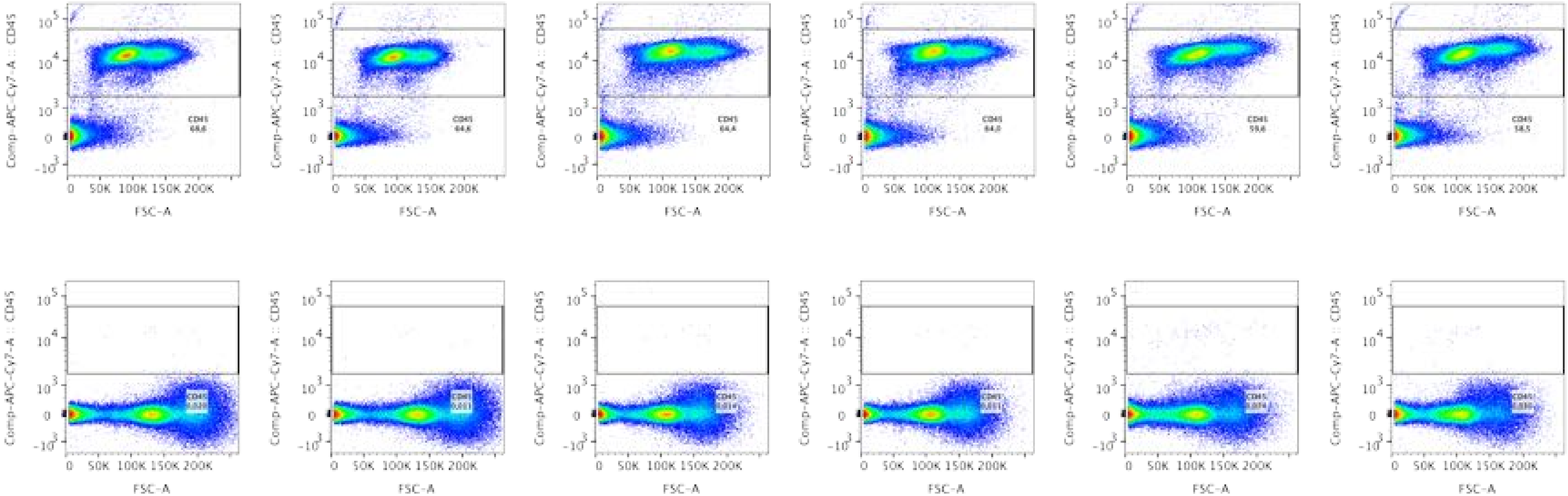

Specificity

Full stained

FMO CD56

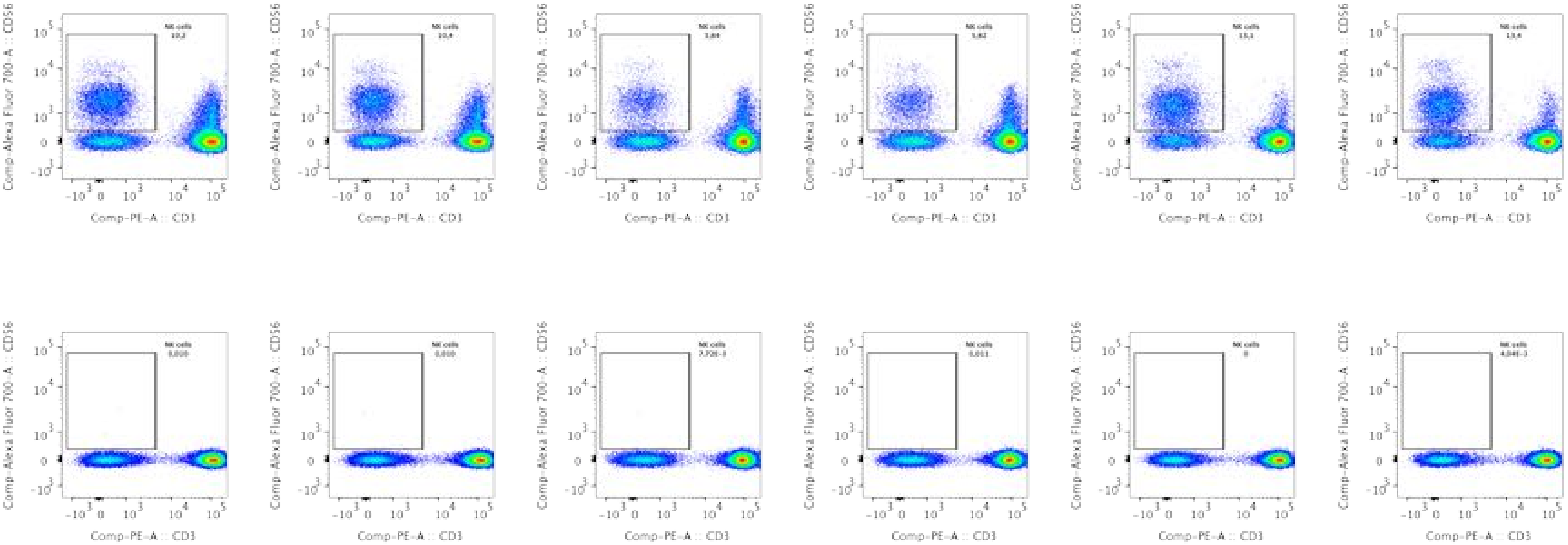

Specificity

Full stained

FMO CD56

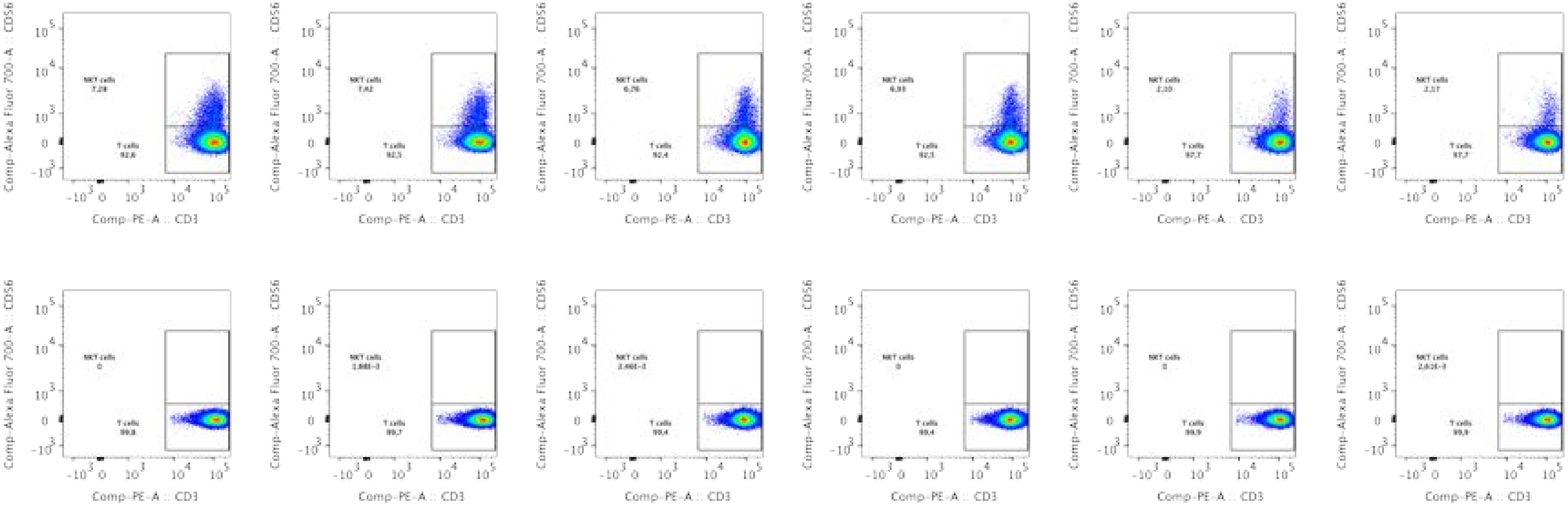

Specificity

Full stained
CD3 cells APO

FMO Apotracker

FMO 7AAD

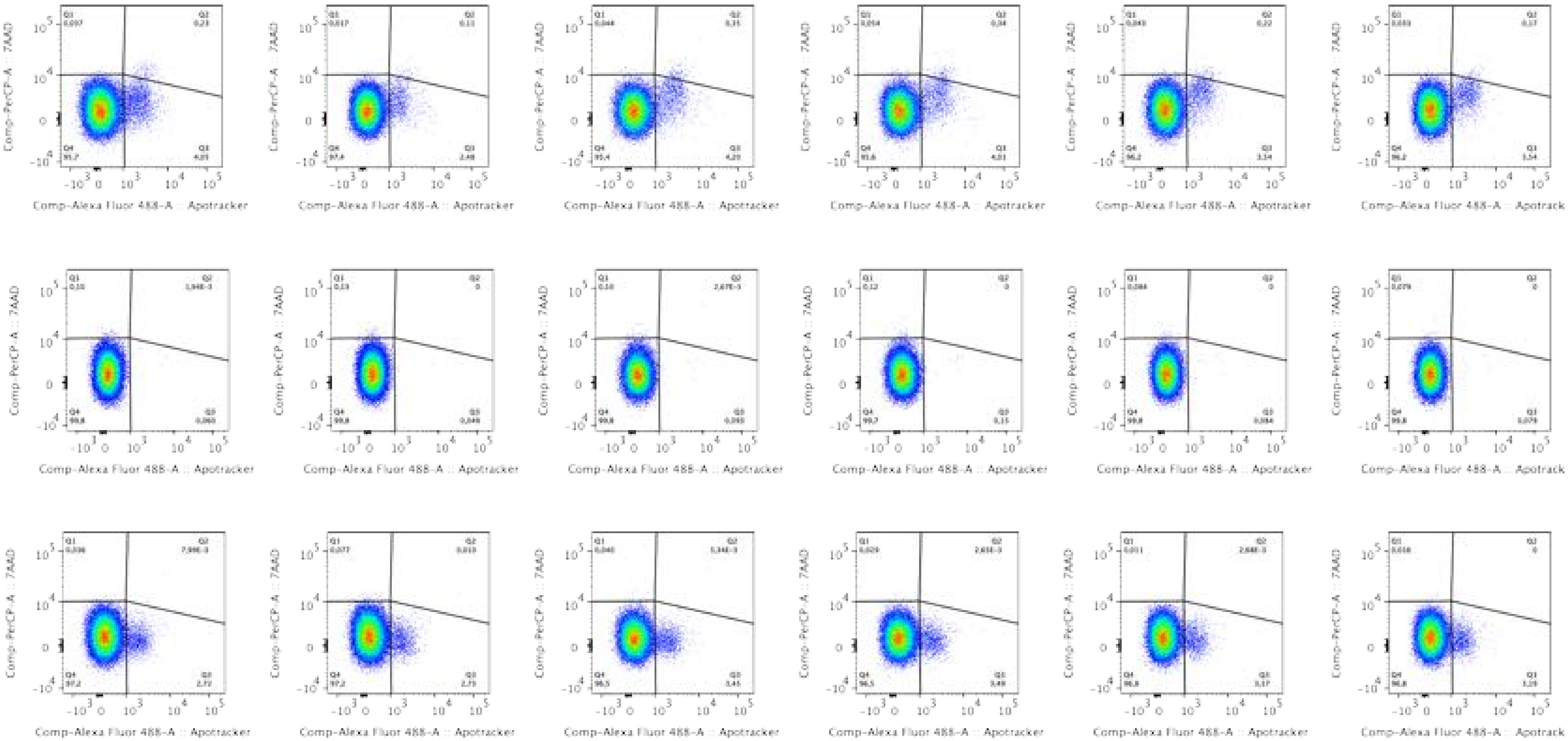

Specificity

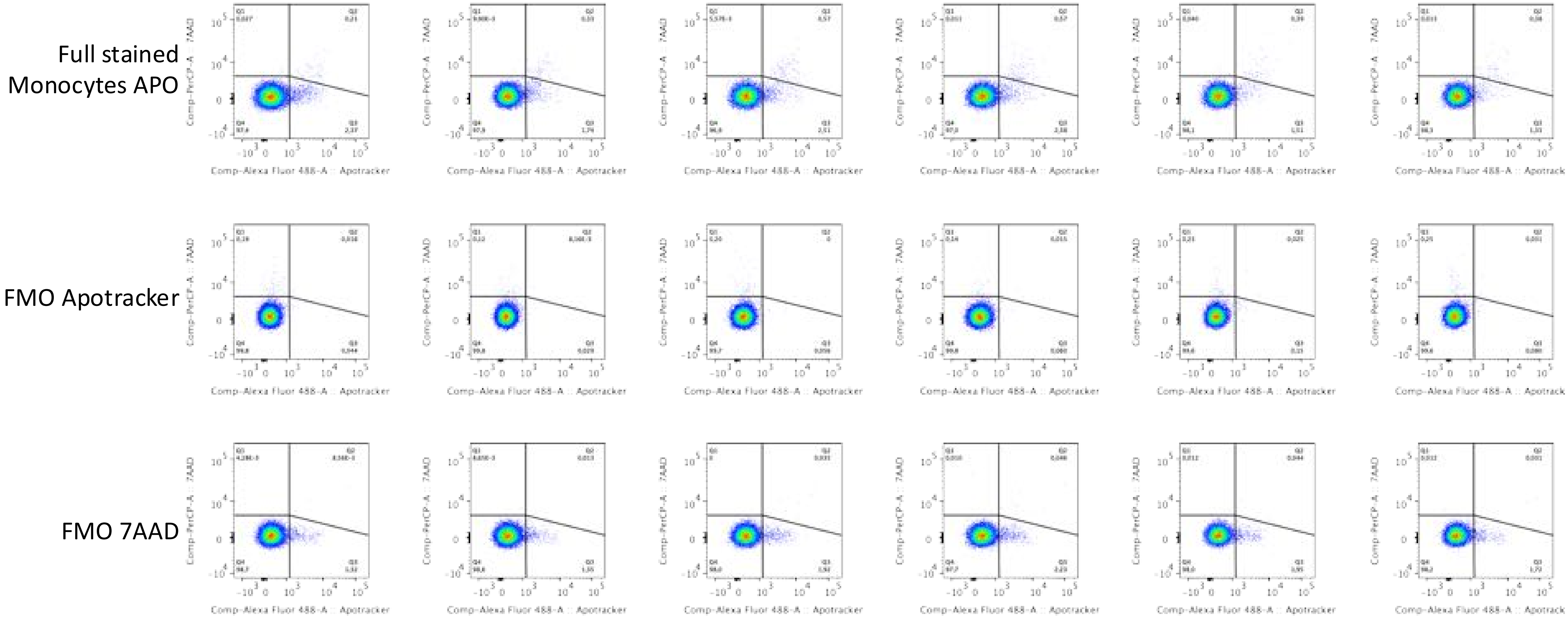

Specificity

Full stained
B cells APO

FMO Apotracker

FMO 7AAD

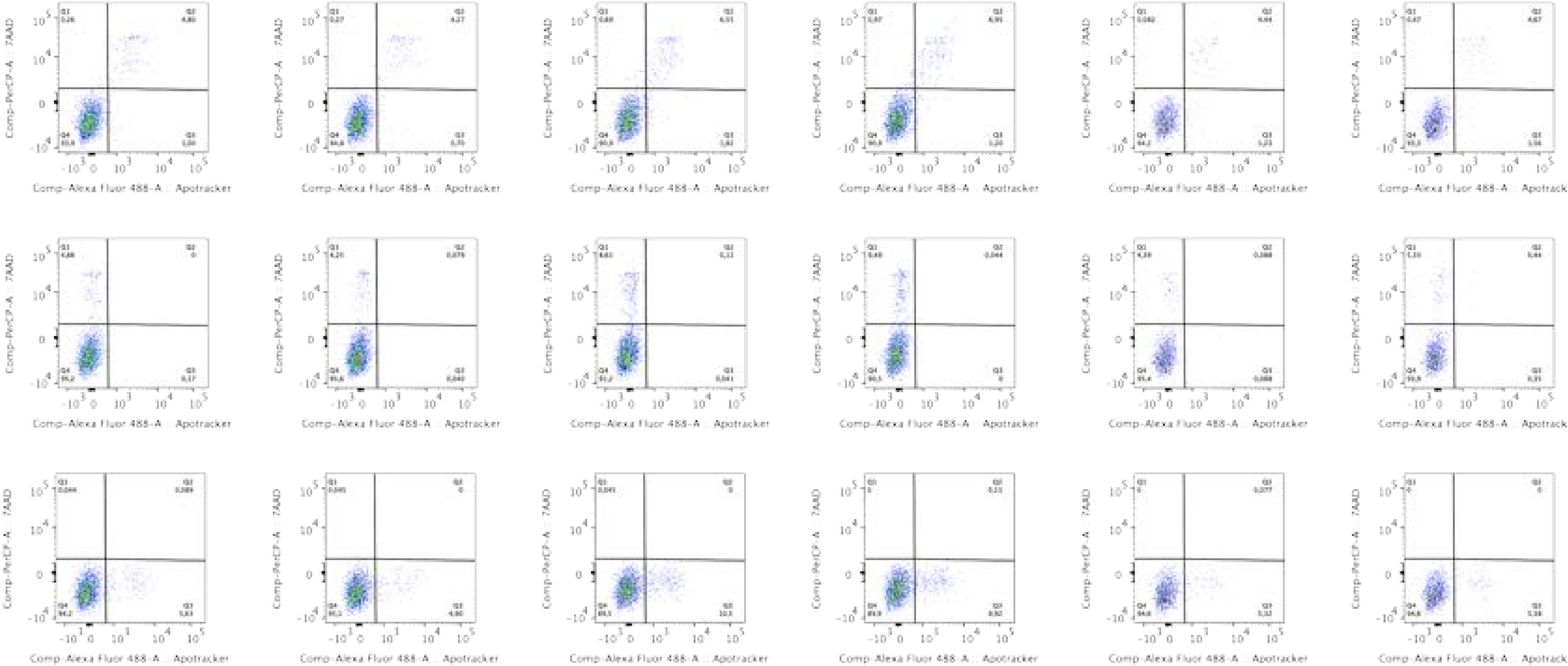

Specificity

Full stained CD45 cells APO

FMO Apotracker

FMO 7AAD

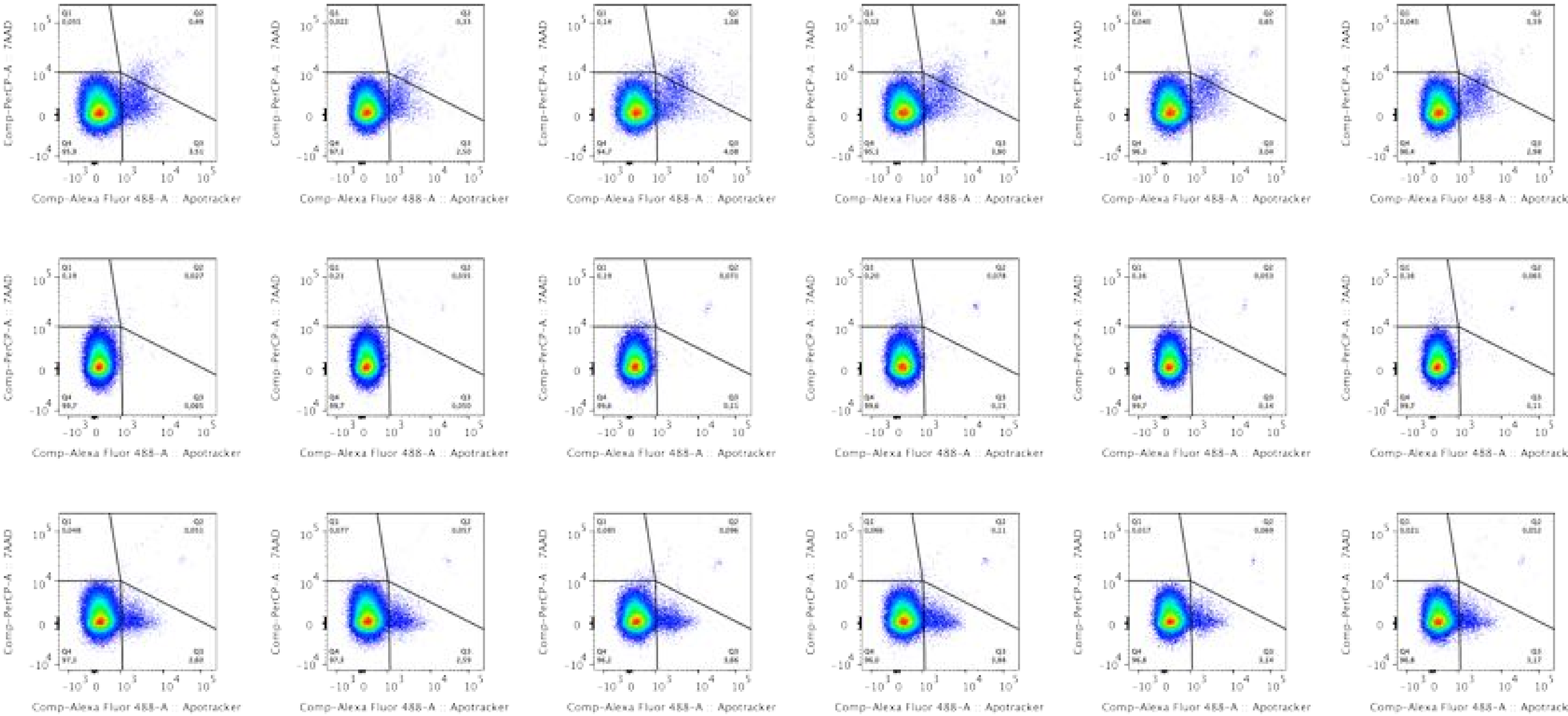

Specificity

Full stained NK cells APO

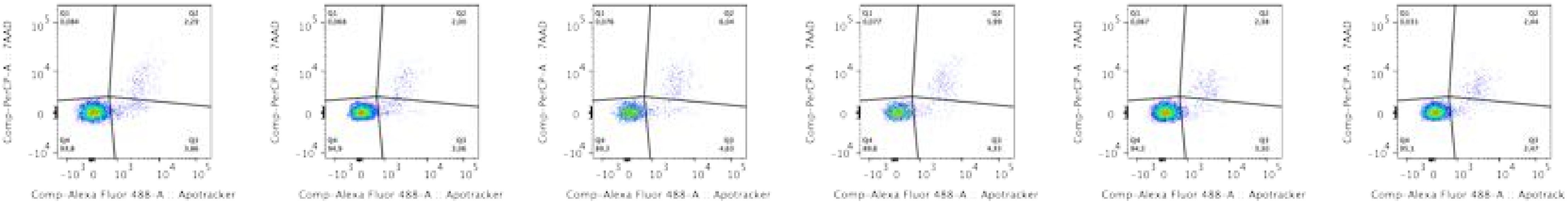

FMO Apotracker

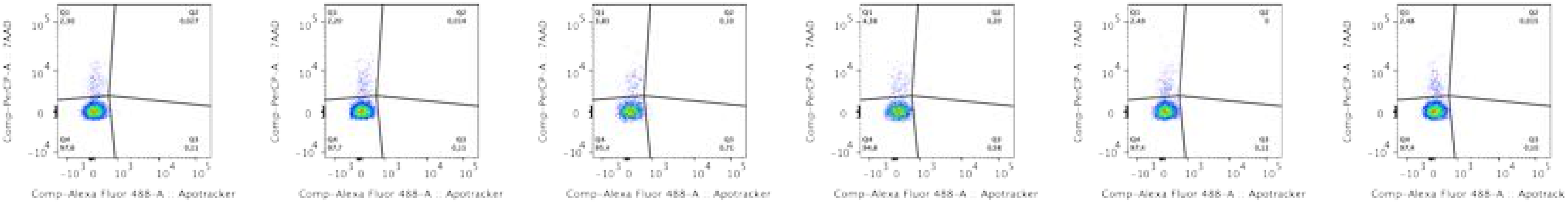

FMO 7AAD

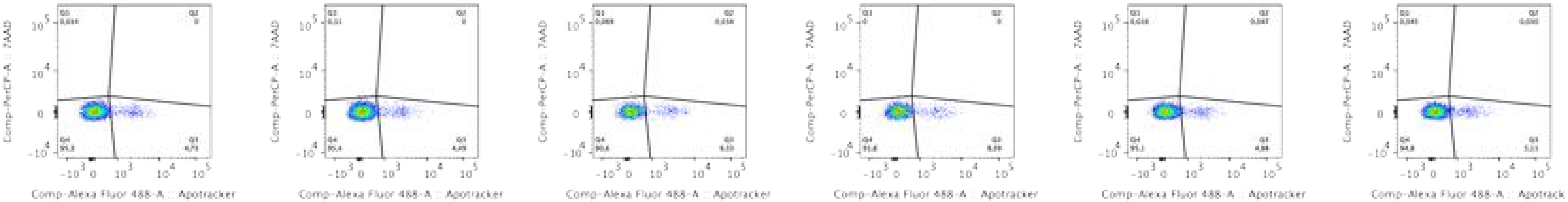

Specificity

Full stained NKT cells APO

FMO Apotracker

FMO 7AAD

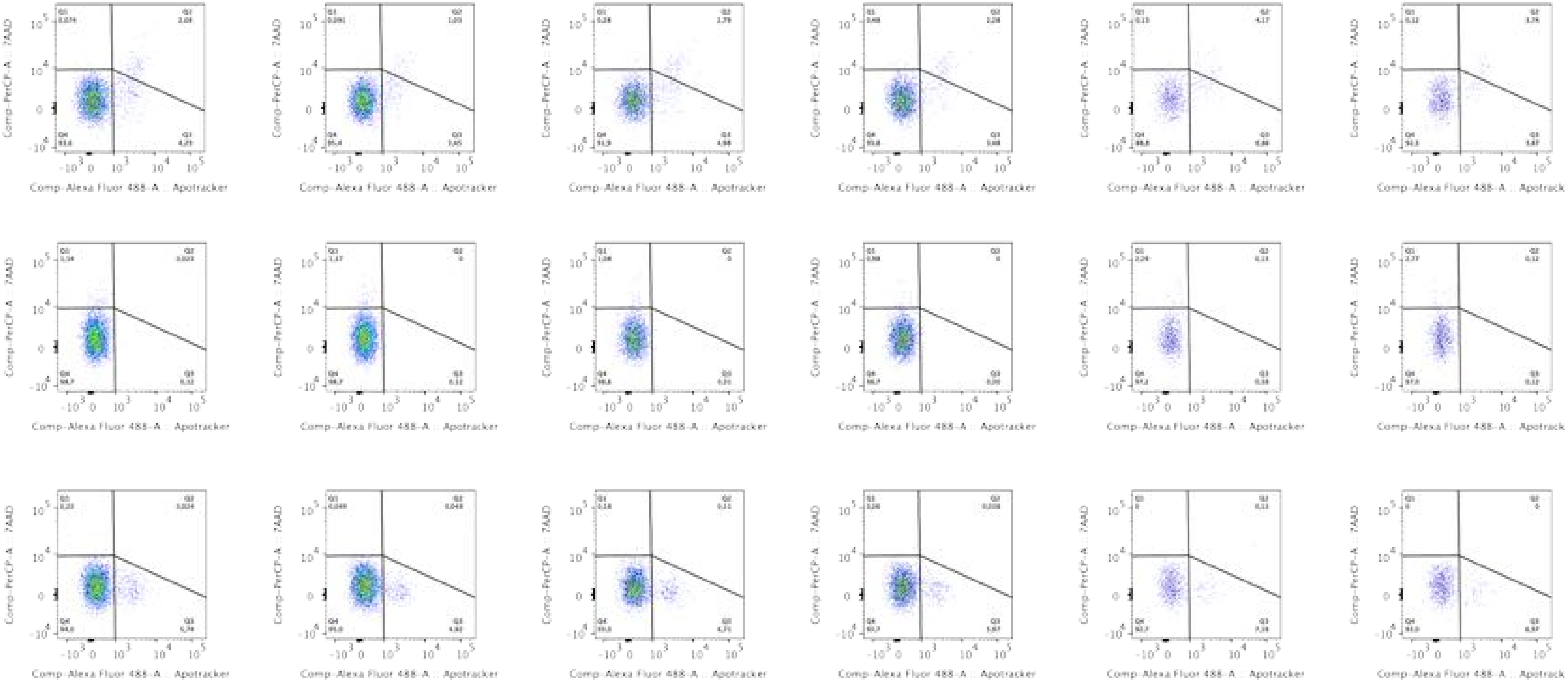

Sensitivity

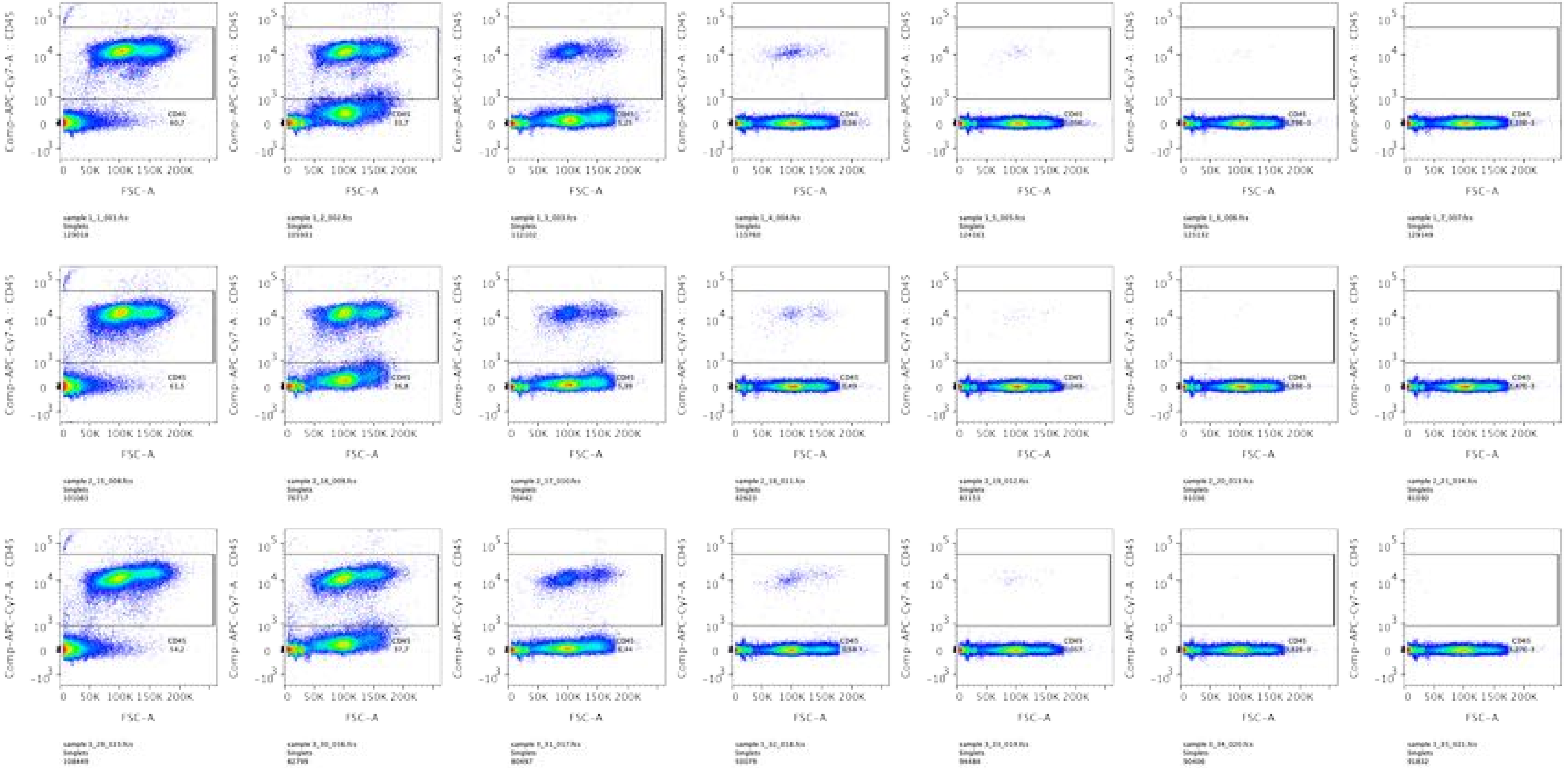

Sample1

Sample2

Sample3

Sensitivity

Sample1

Sample2

Sample3

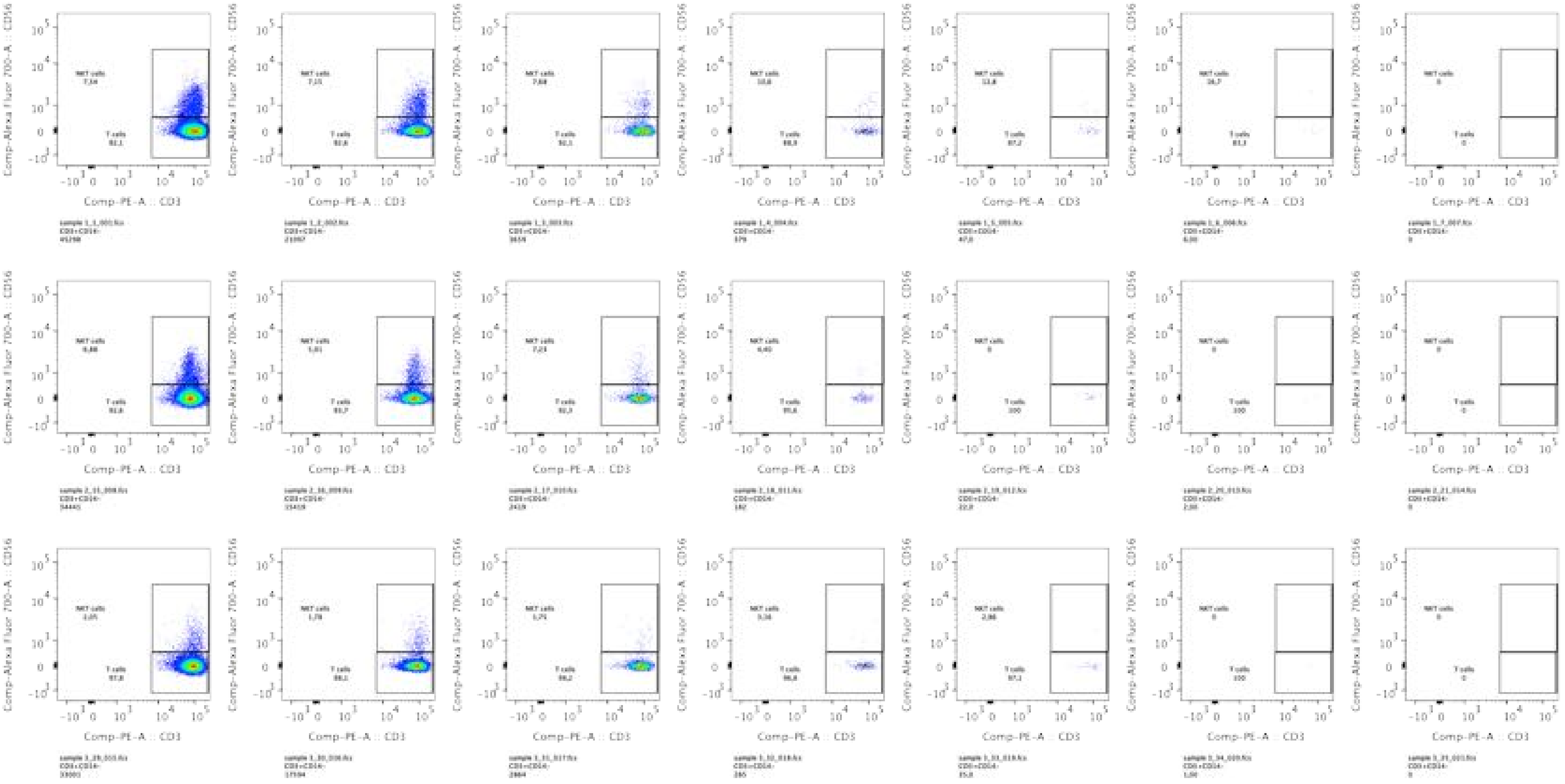

Sensitivity

Sample1

Sample2

Sample3

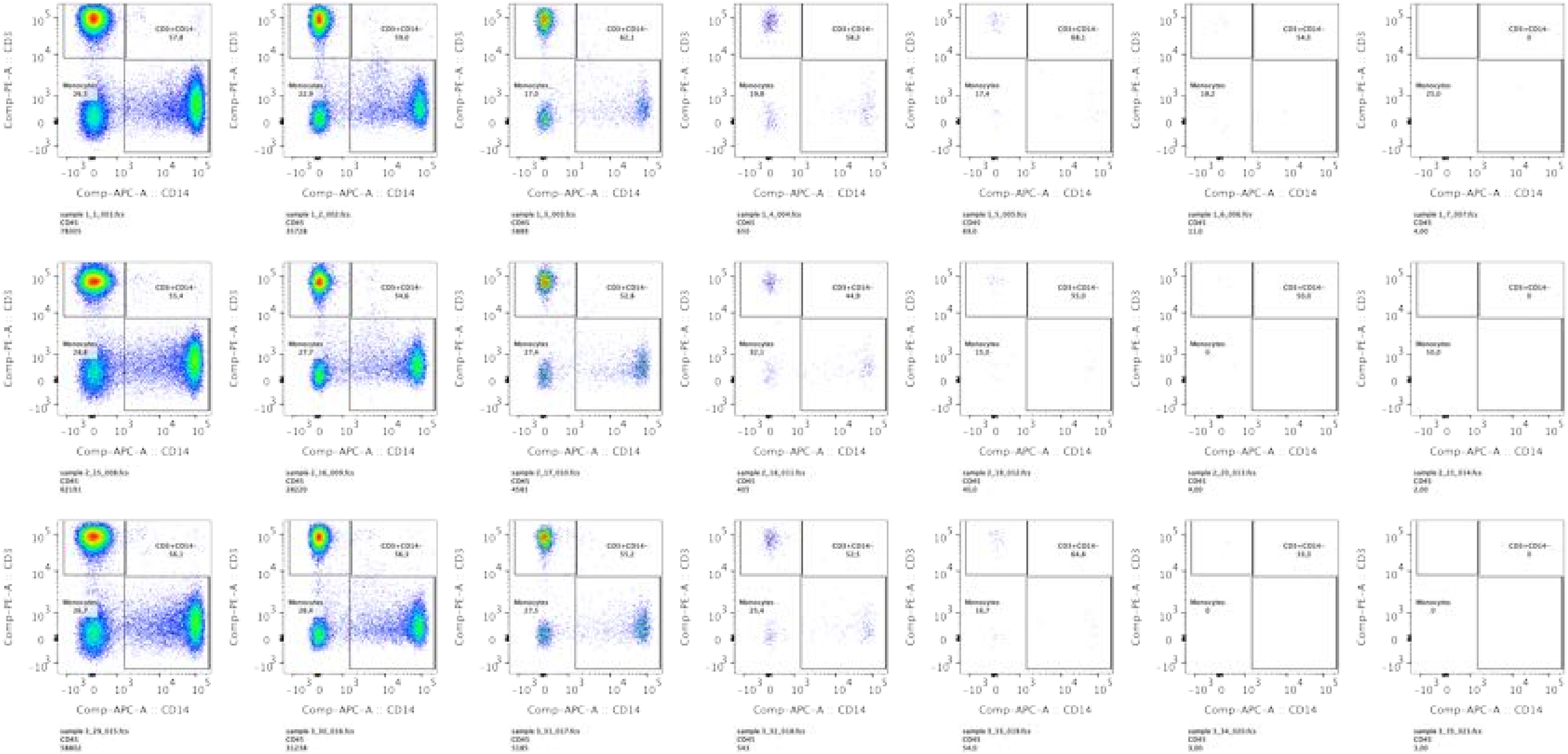

Sensitivity

Sample1

Sample2

Sample3

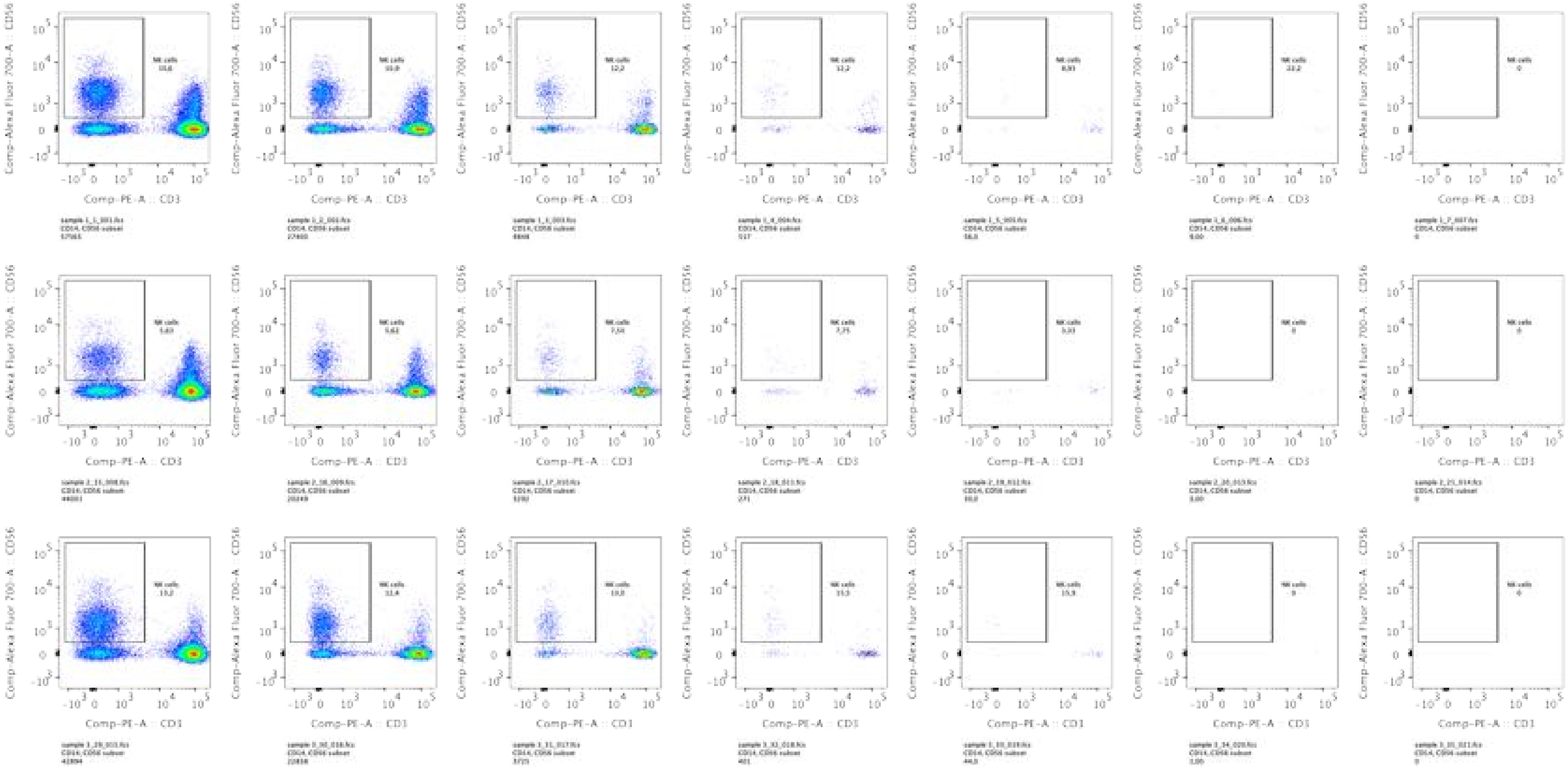

Sensitivity

Sample1

Sample2

Sample3

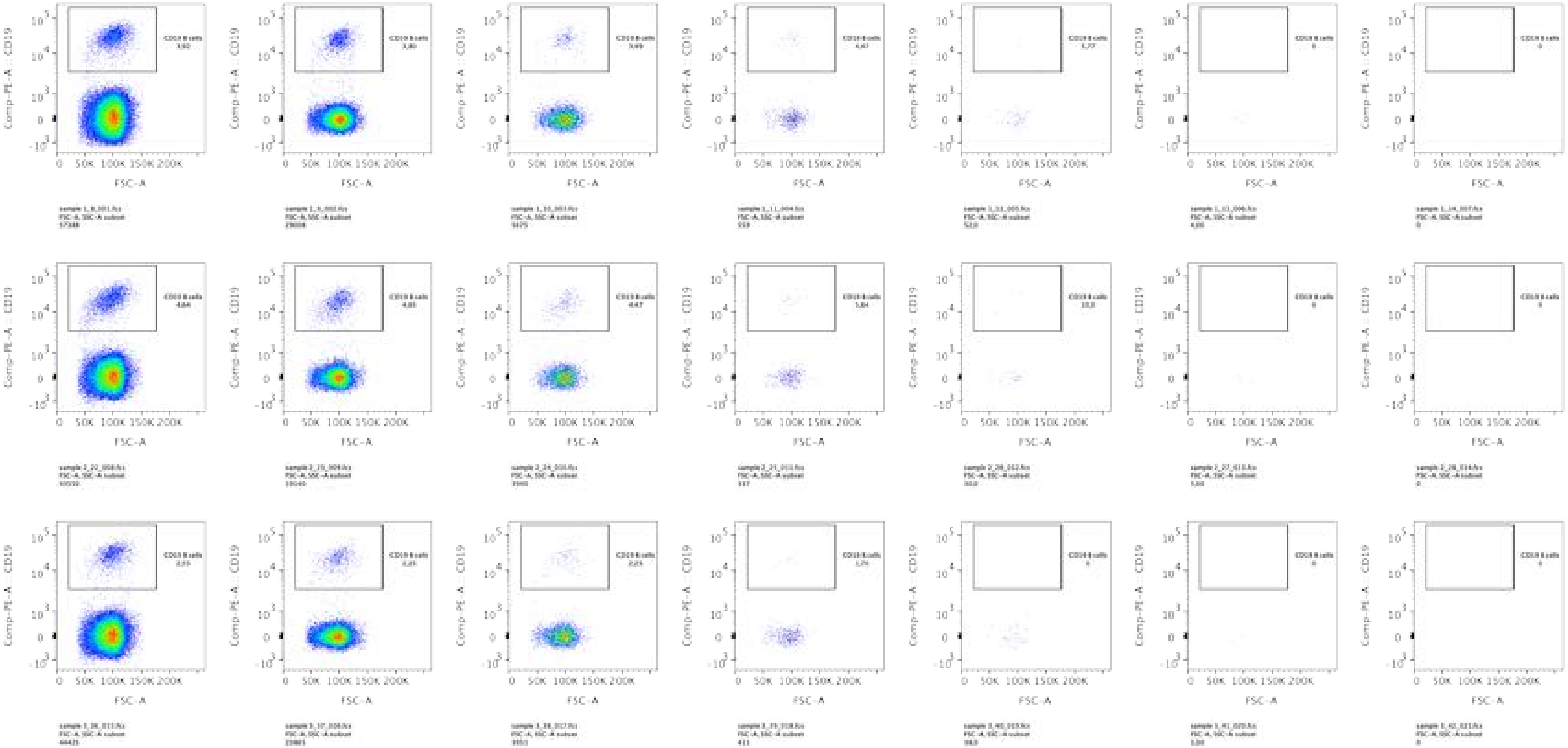

Inter-instrument: Canto

Intra-assay

Inter-instrument: LSRII

Sample1

Sample2

Sample3

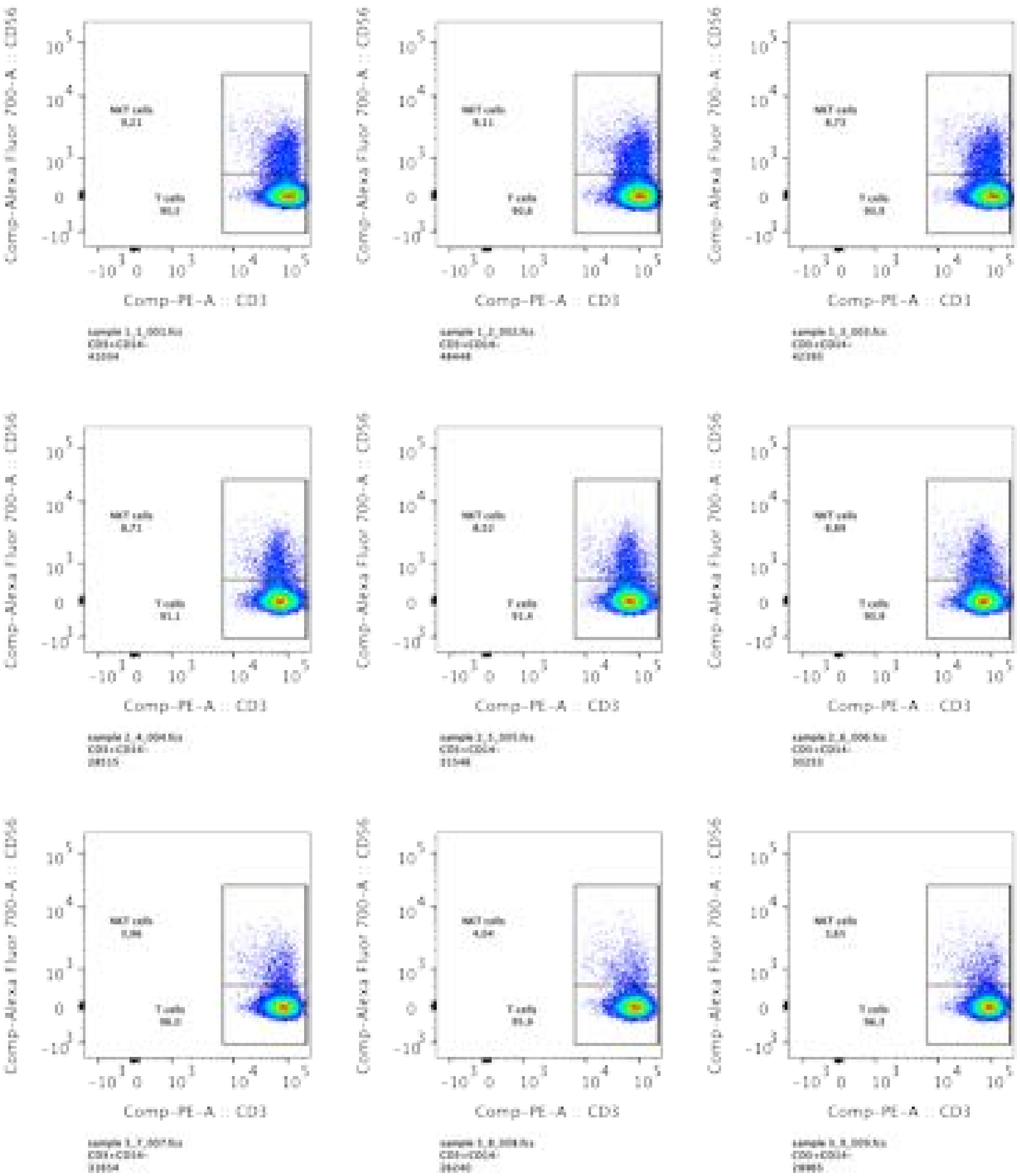

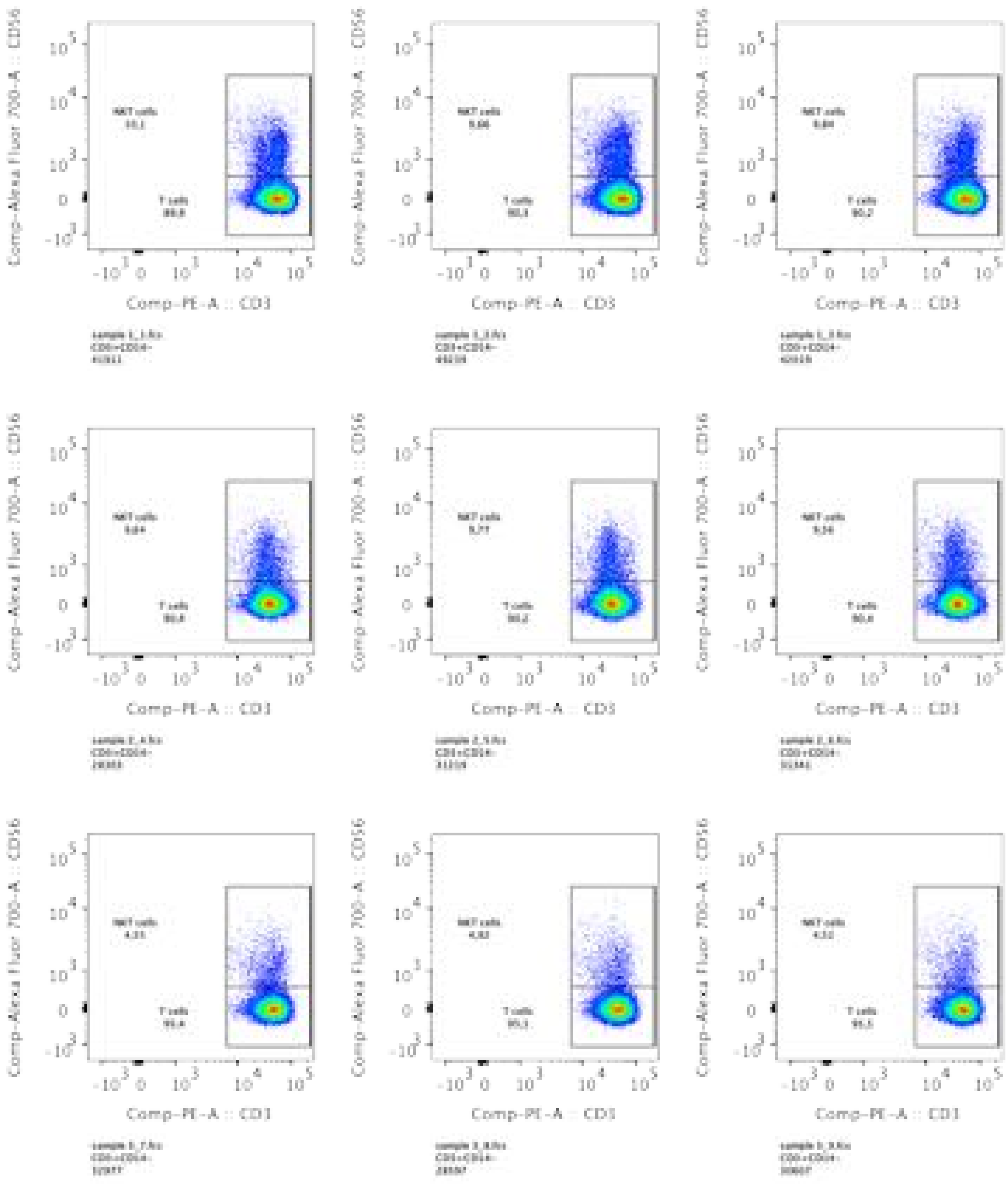

Inter-instrument: Canto

Intra-assay

Inter-instrument: LSRII

Sample1

Sample2

Sample3

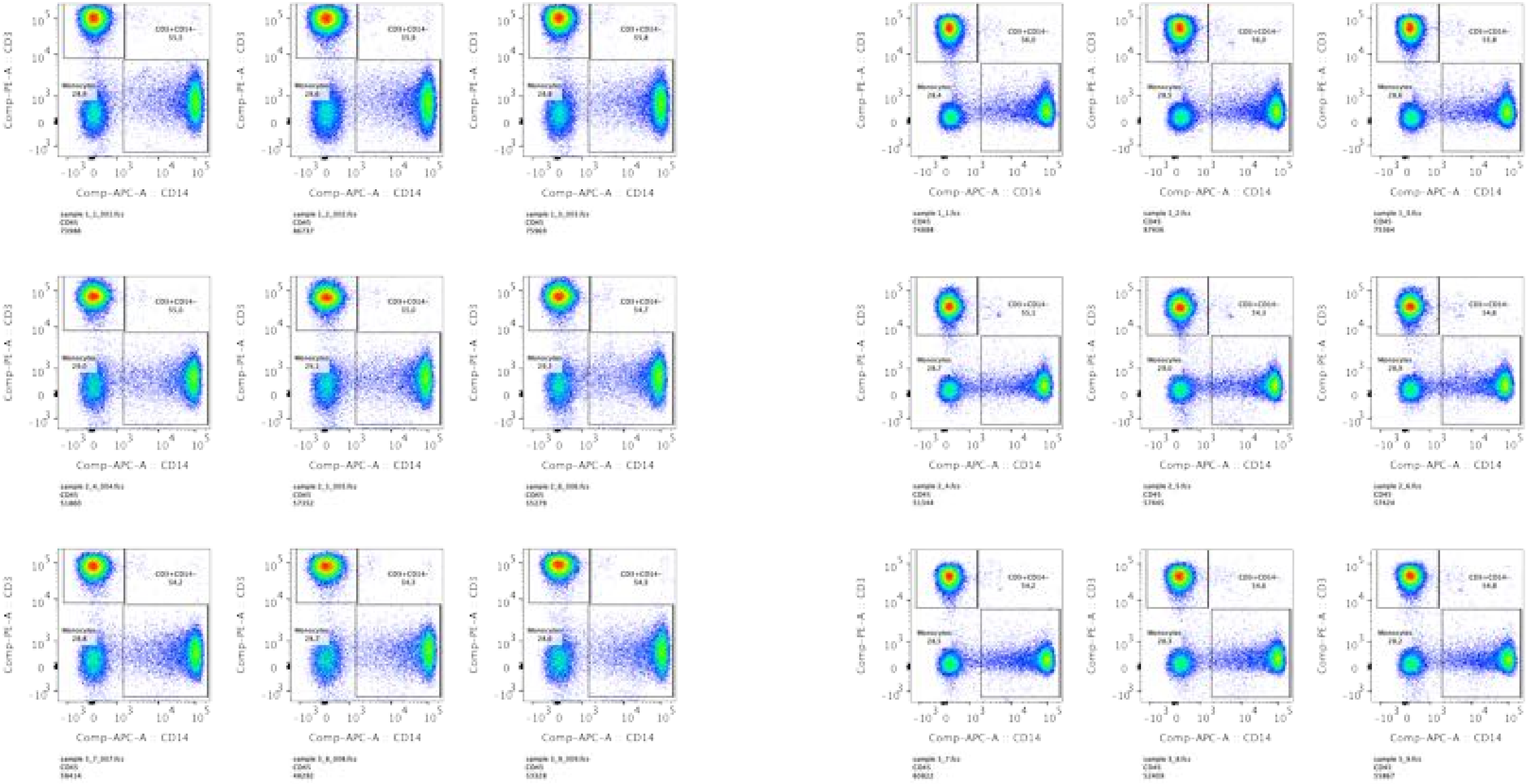

Inter-instrument: Canto

Intra-assay

Inter-instrument: LSRII

Sample1

Sample2

Sample3

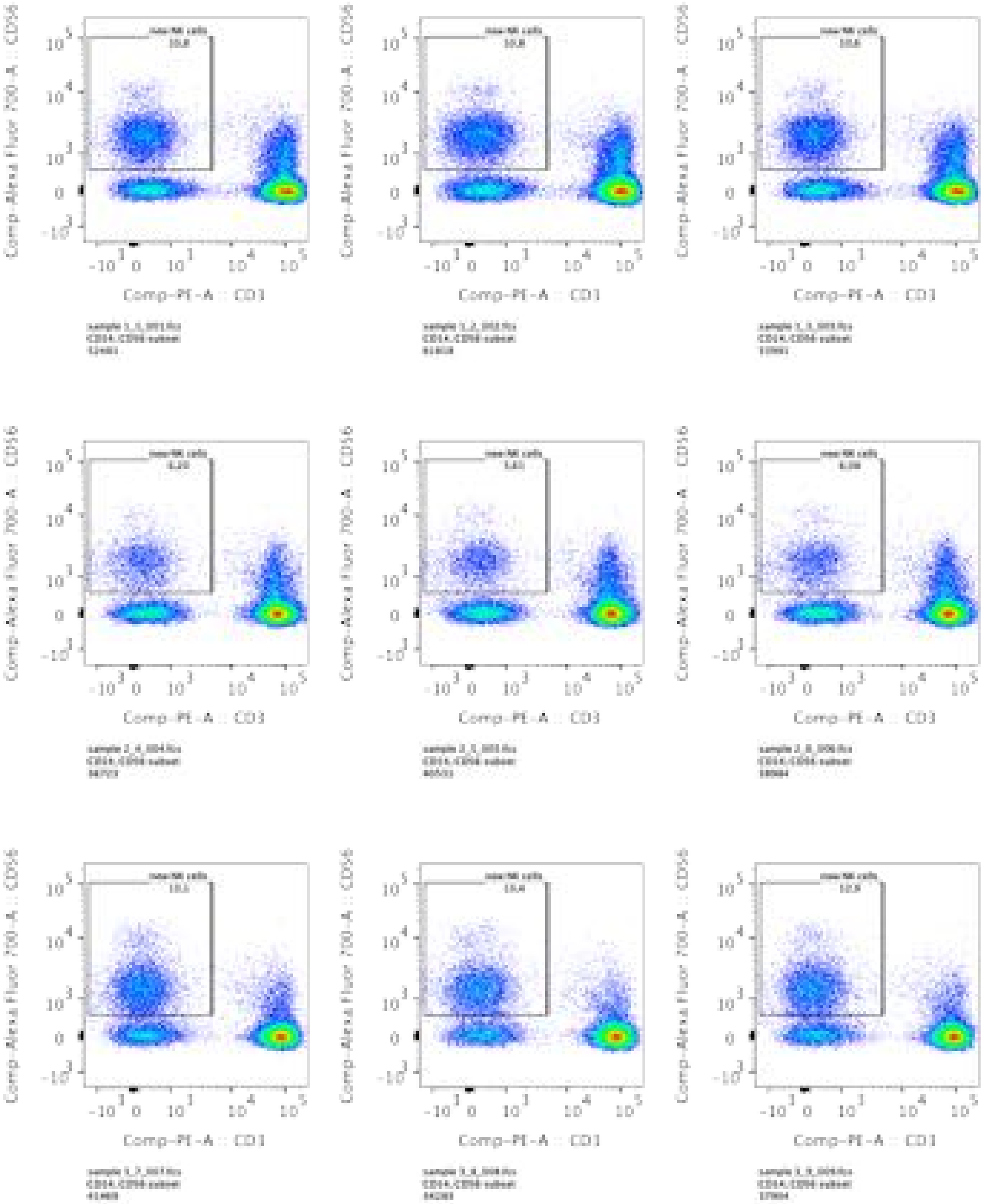

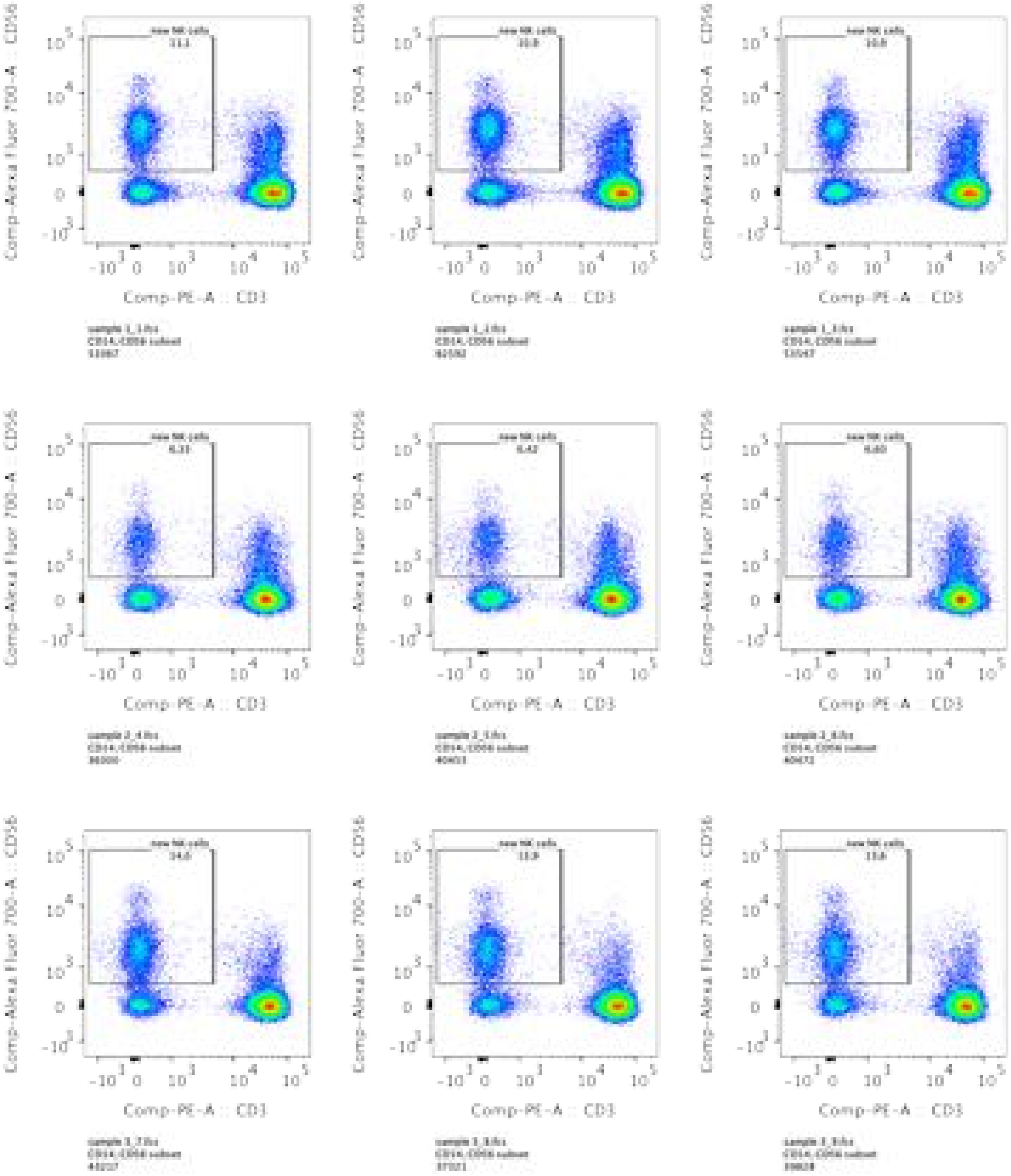

Inter-instrument: Canto

Intra-assay

Inter-instrument: LSRII

Sample1

Sample2

Sample3

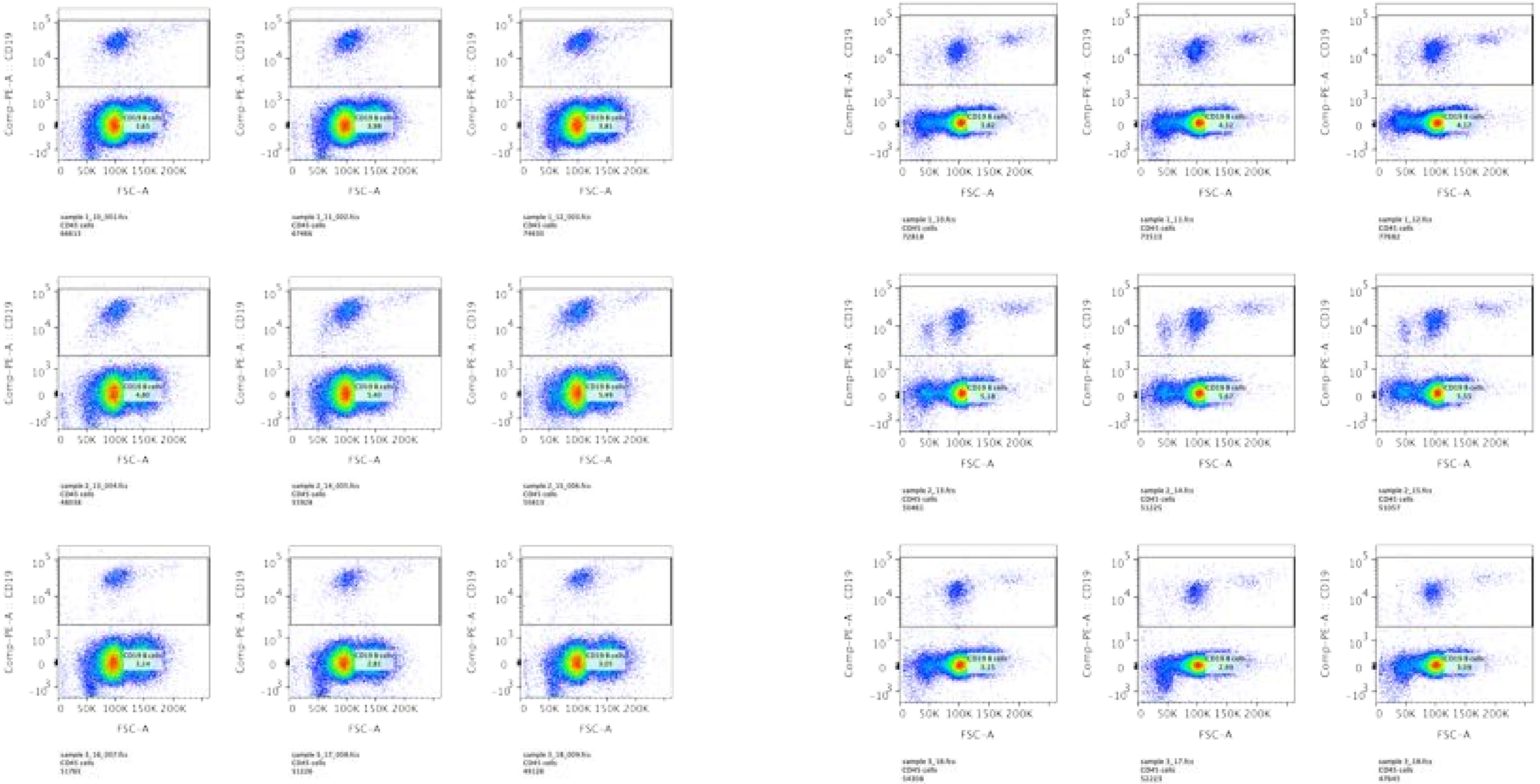

Inter-instrument: Canto

Intra-assay

Inter-instrument: LSRII

Sample1

Sample2

Sample3

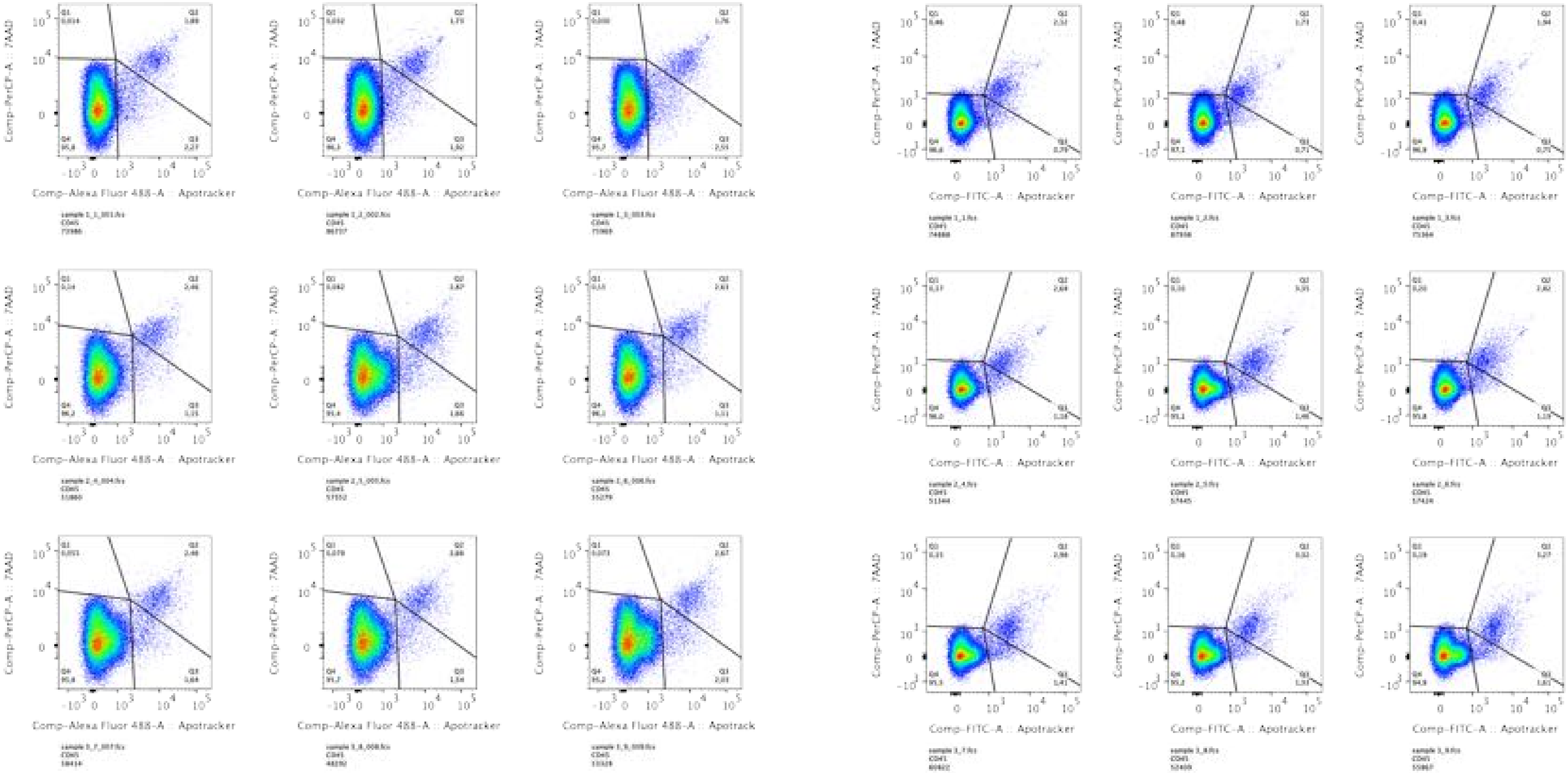

Inter-instrument: Canto

Intra-assay

Inter-instrument: LSRII

Sample1

Sample2

Sample3

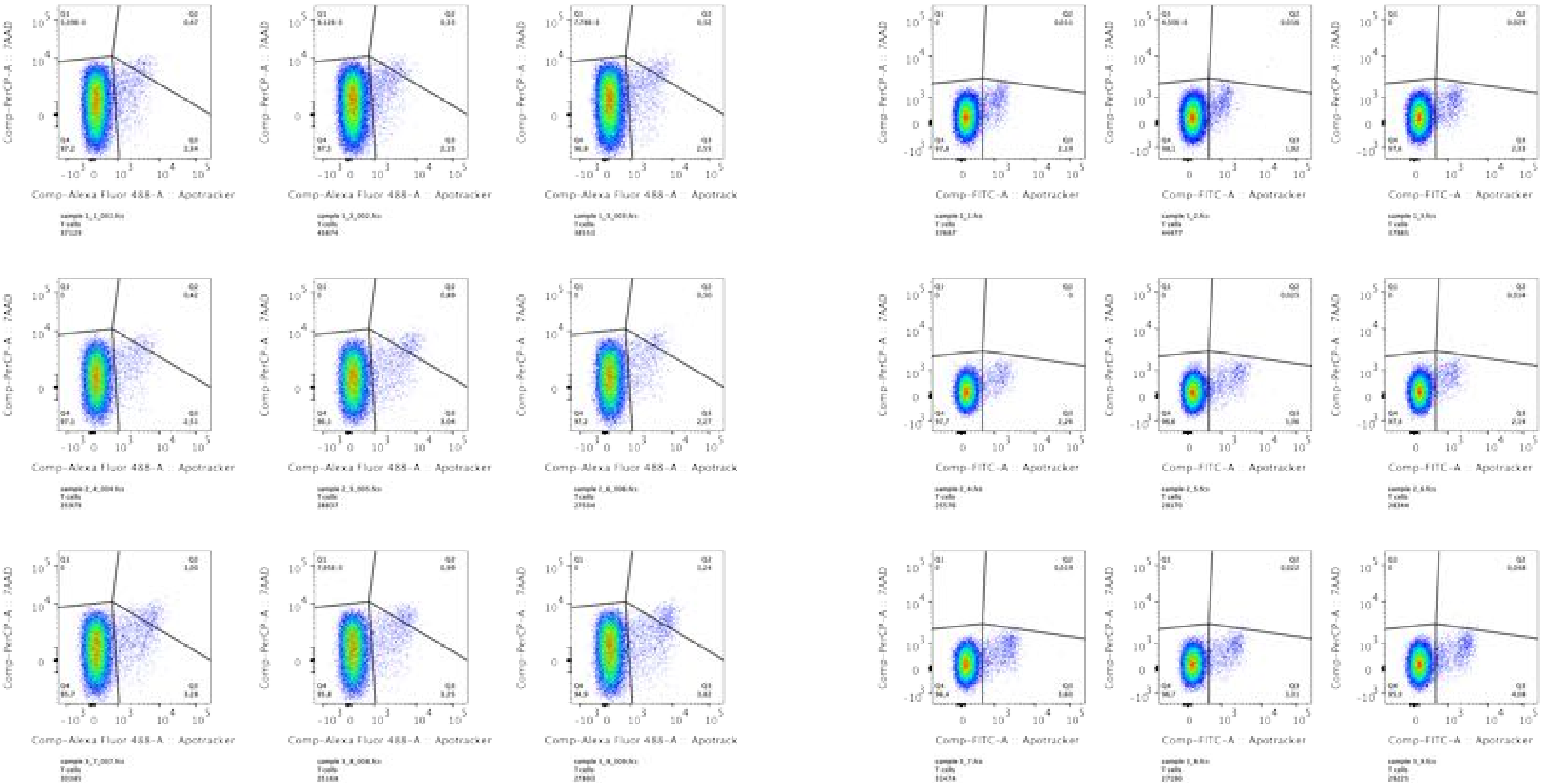

Inter-instrument: Canto

Intra-assay

Inter-instrument: LSRII

Sample1

Sample2

Sample3

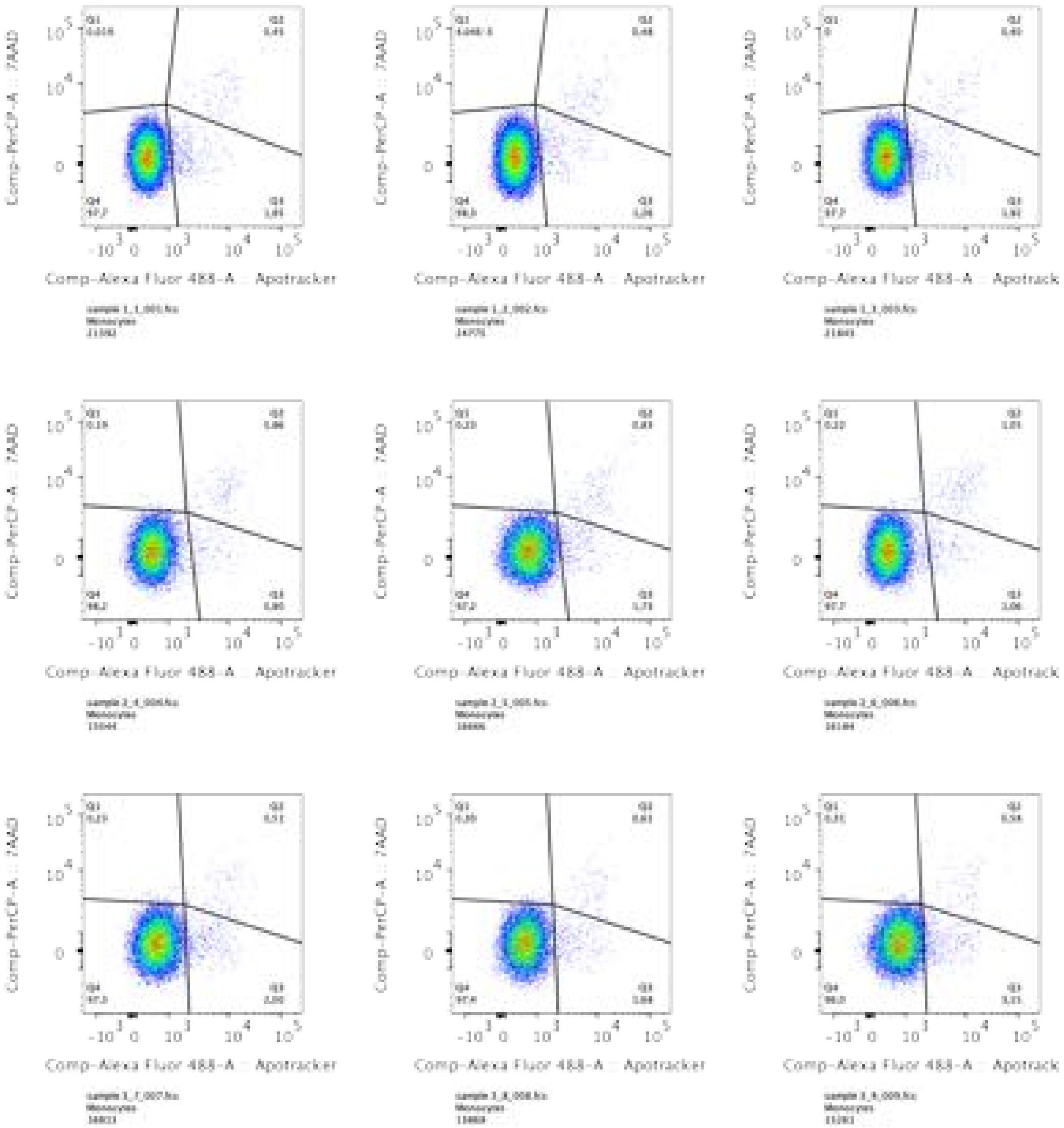

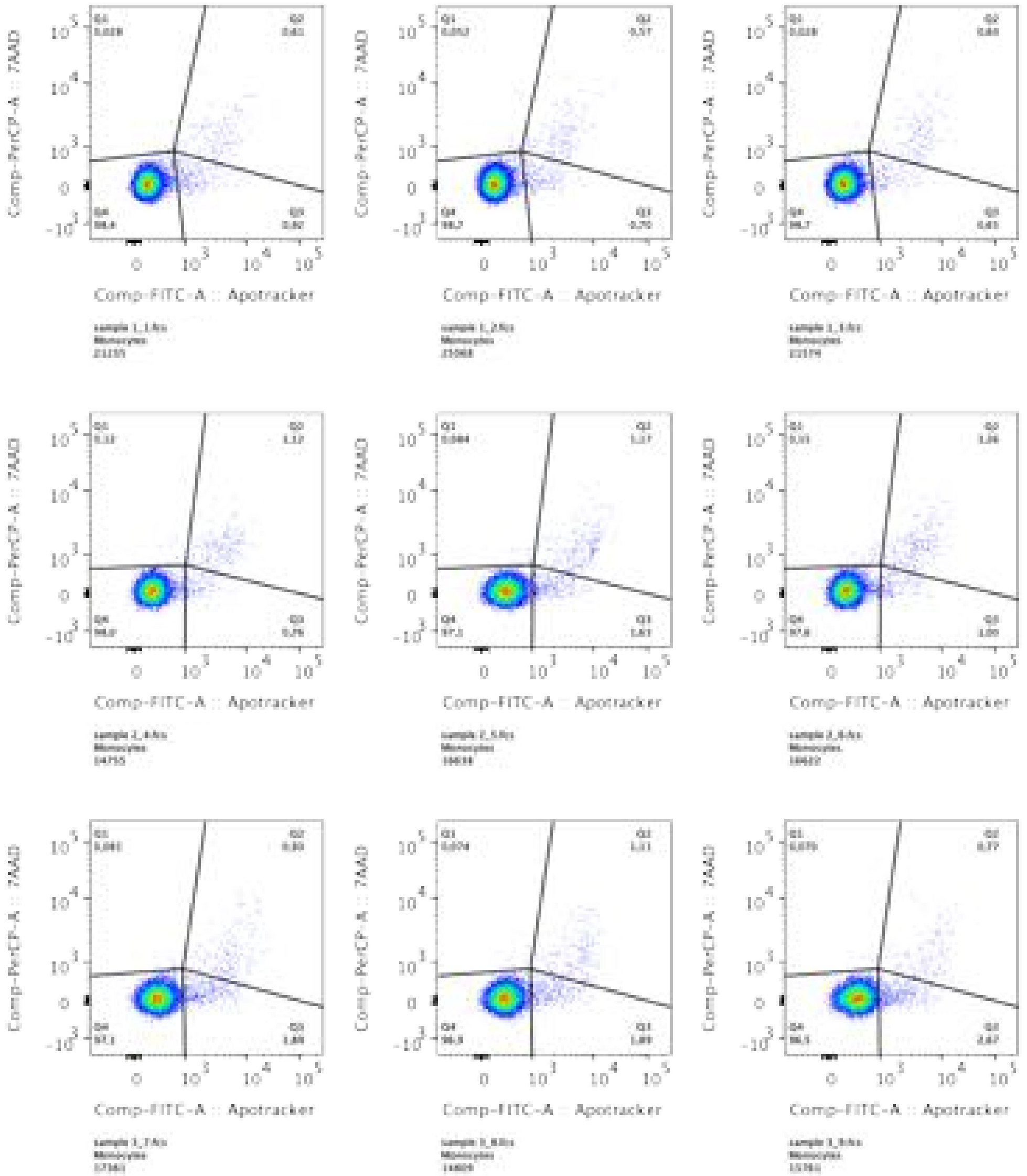

Inter-instrument: Canto

Intra-assay

Inter-instrument: LSRII

Sample1

Sample2

Sample3

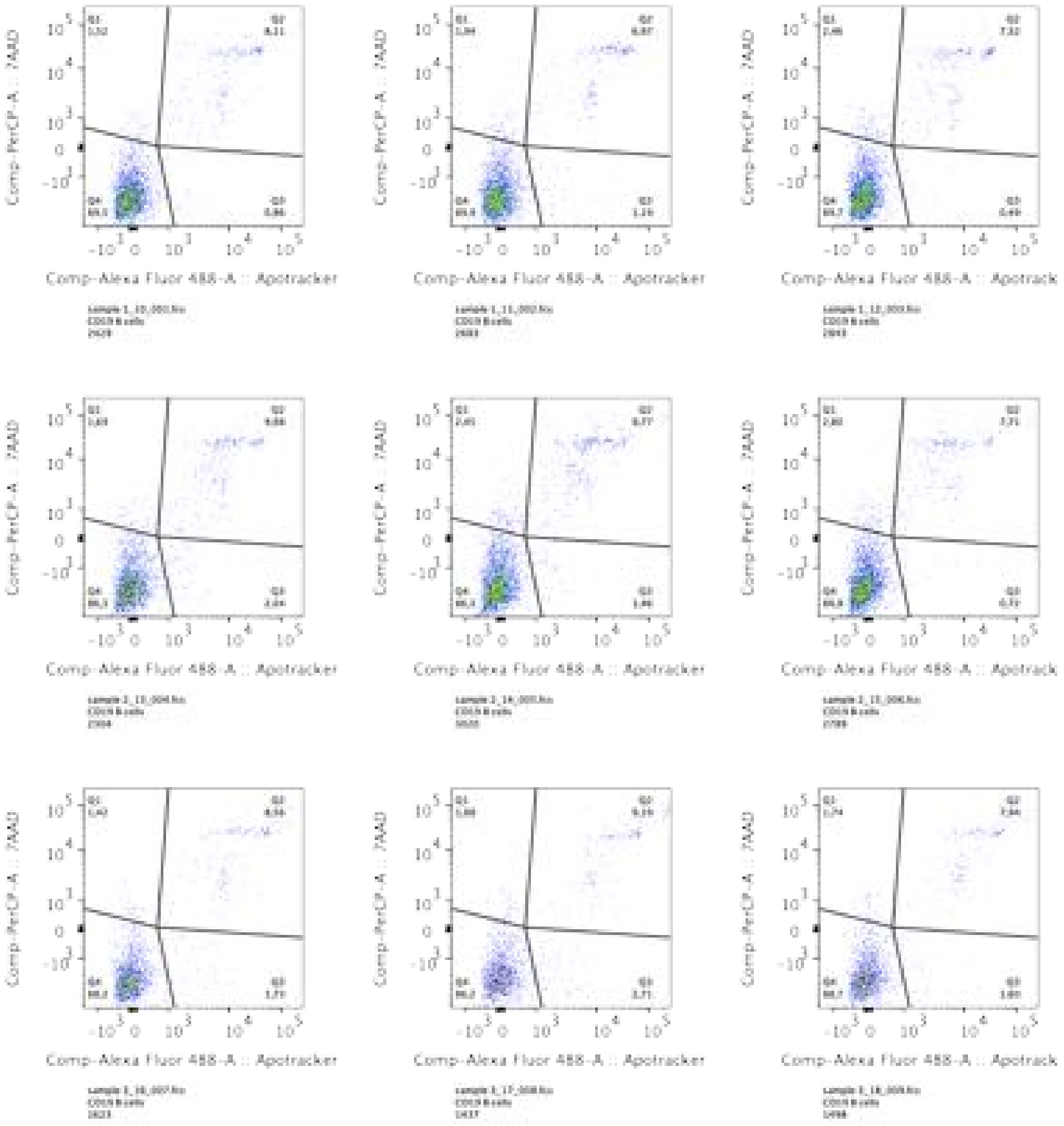

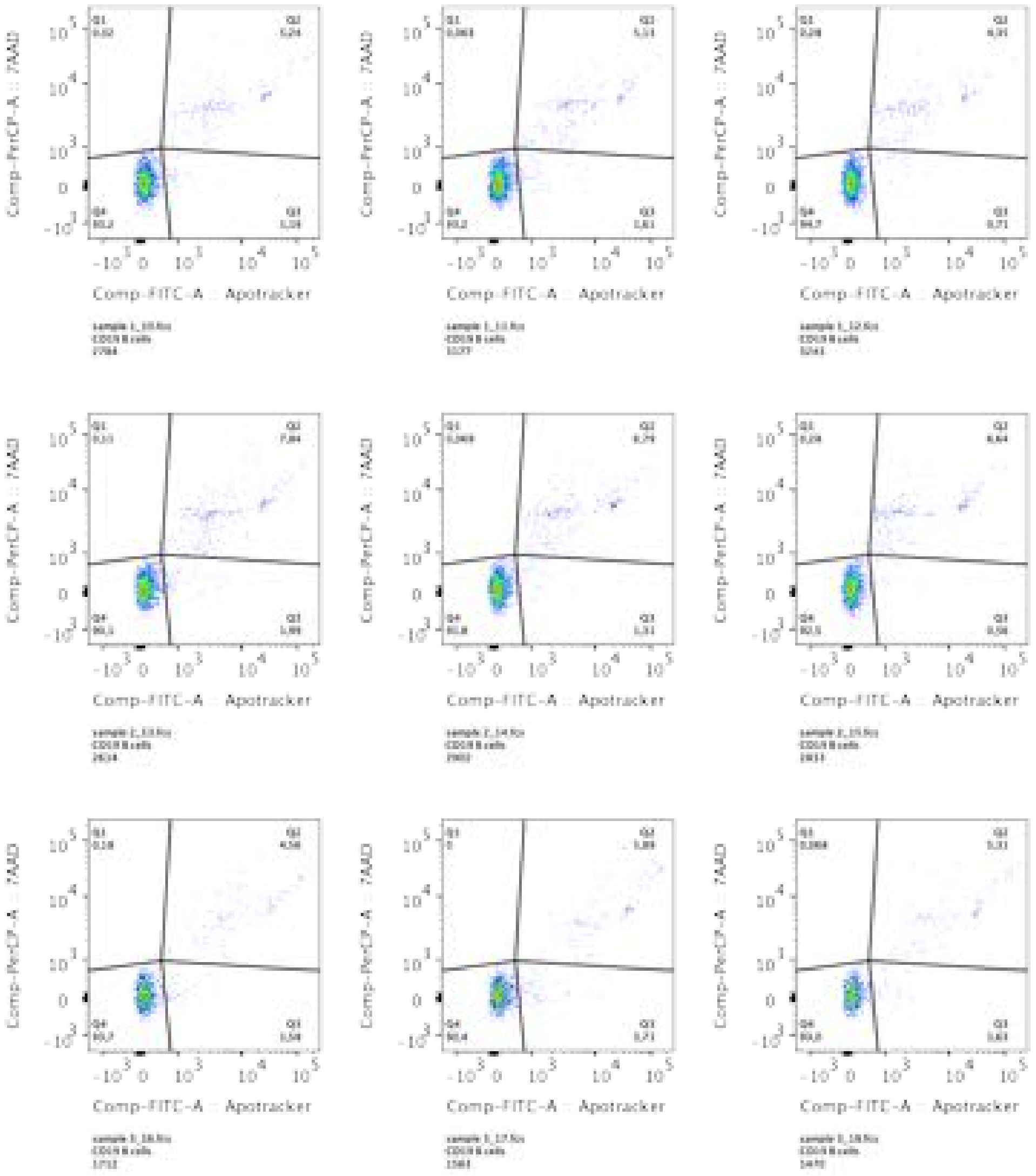

Inter-instrument: Canto

Inter-instrument: LSRII

Intra-assay

Sample1

Sample2

Sample3

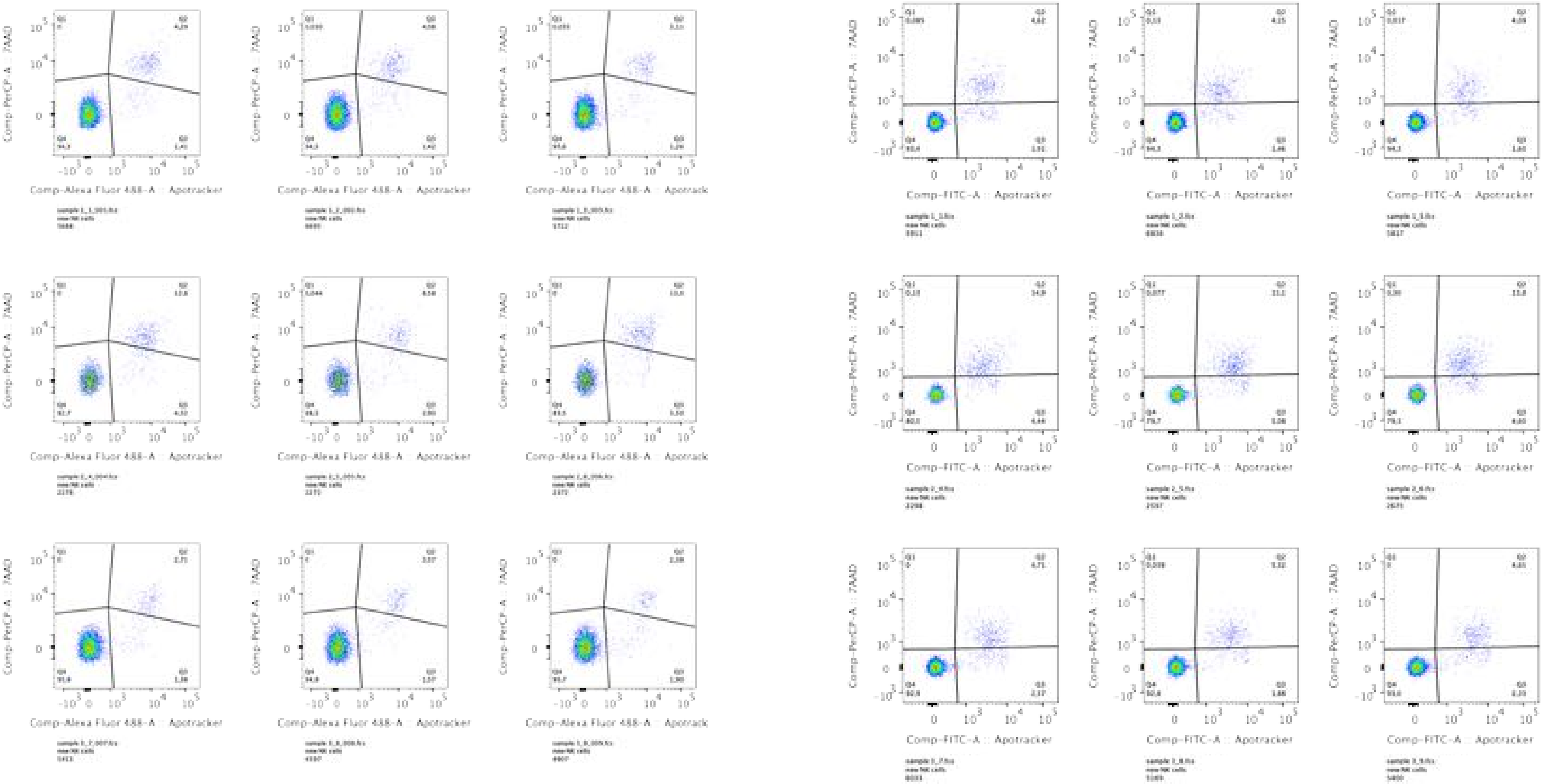

Inter-instrument: Canto

Intra-assay

Inter-instrument: LSRII

Sample1

Sample2

Sample3

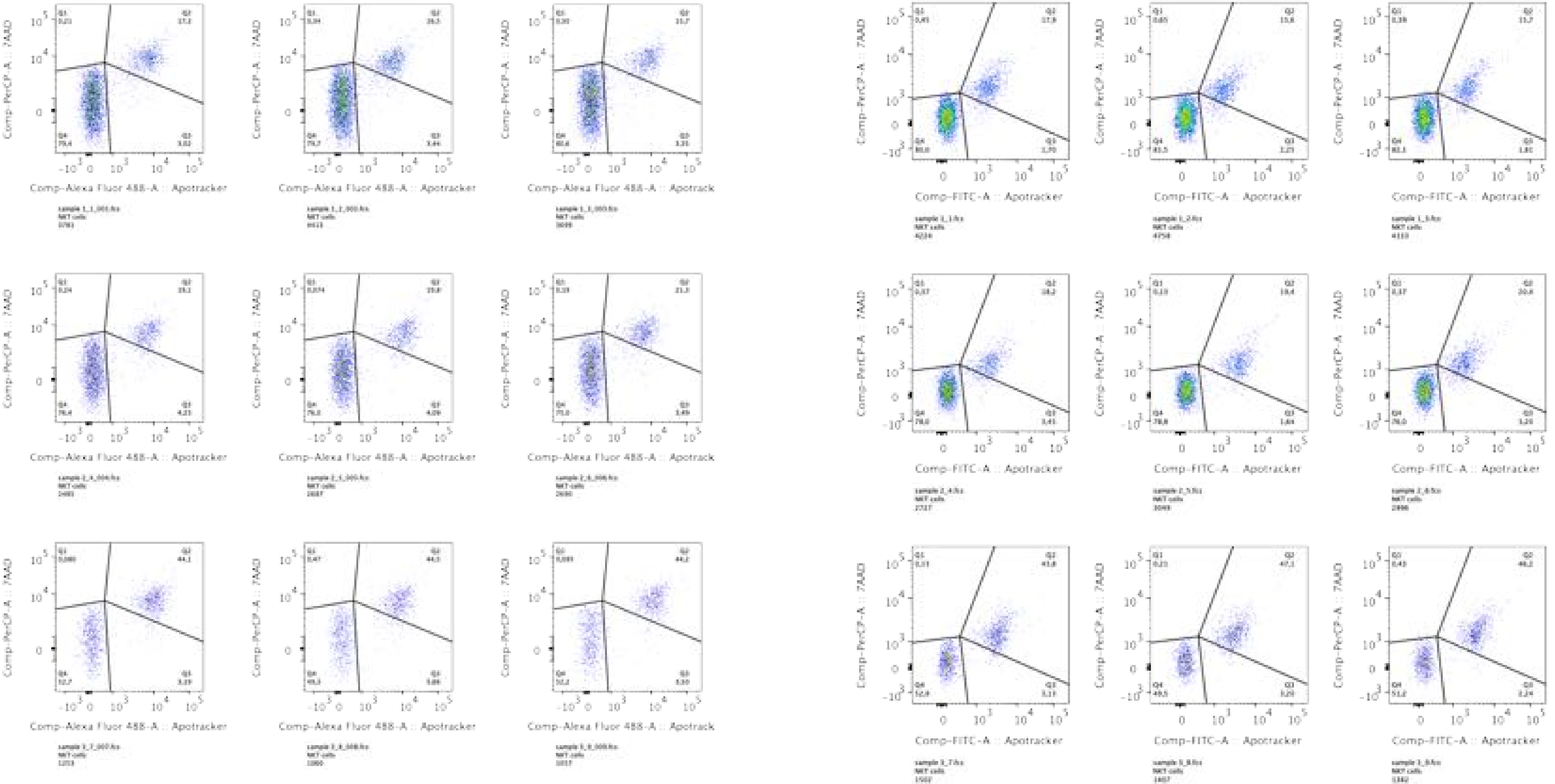

Inter-operator

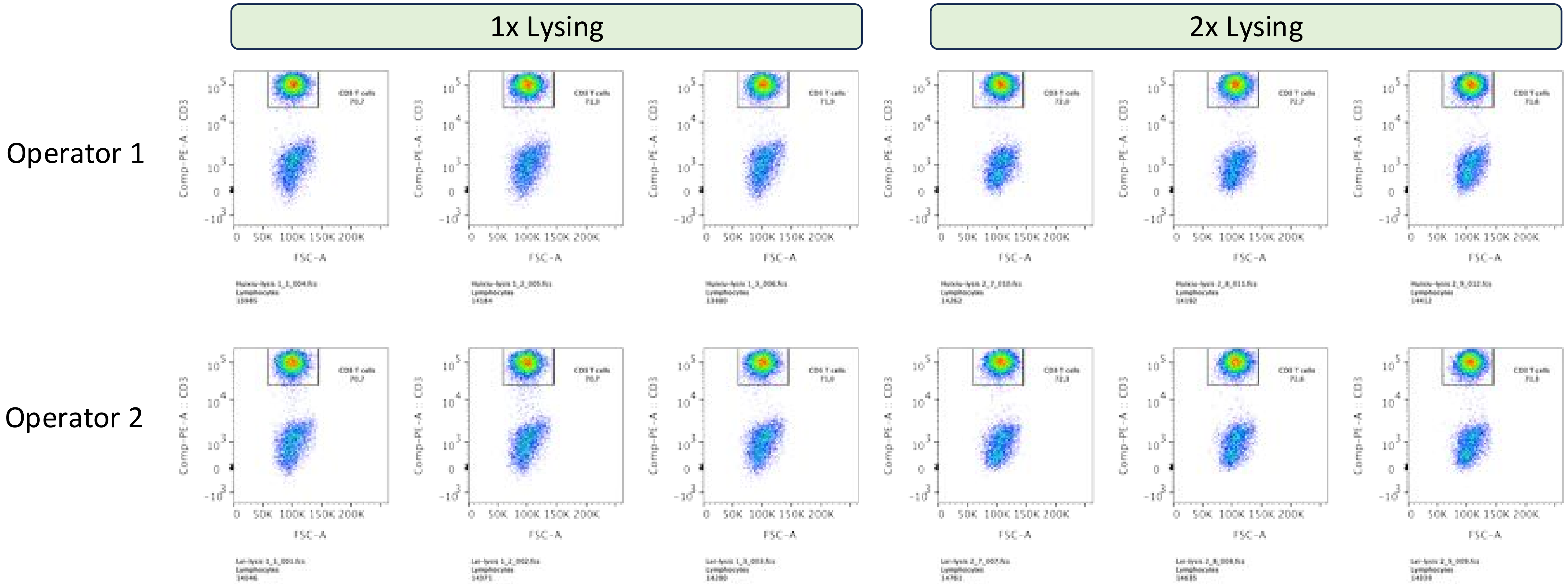

Inter-operator

1x Lysing

2x Lysing

Operator 1

Operator 2

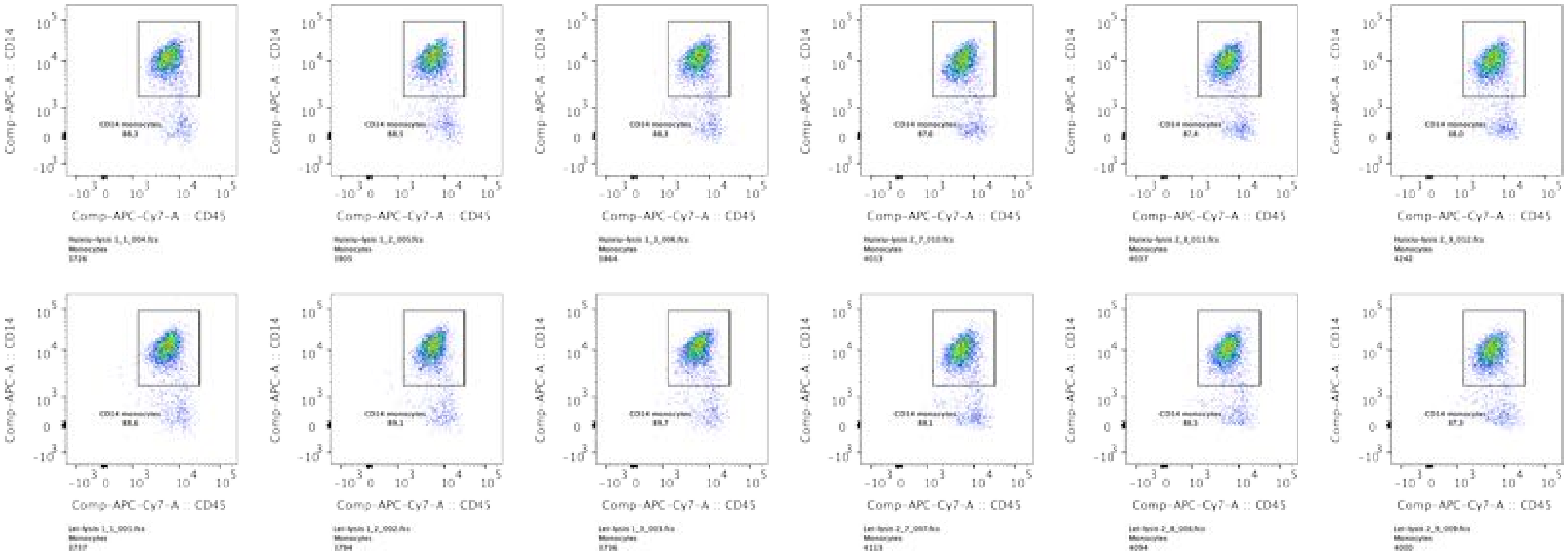

Inter-operator

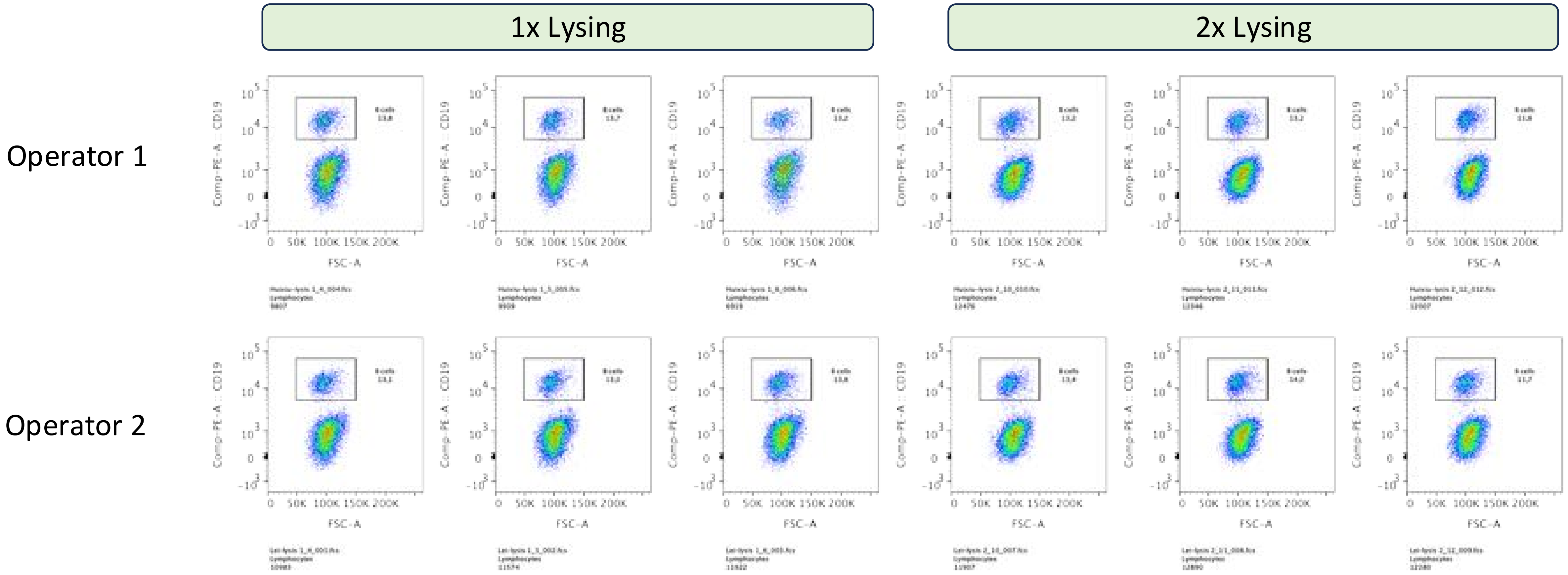

Inter-operator

	1x Lysing	2x Lysing
Operator 1		
Operator 2		

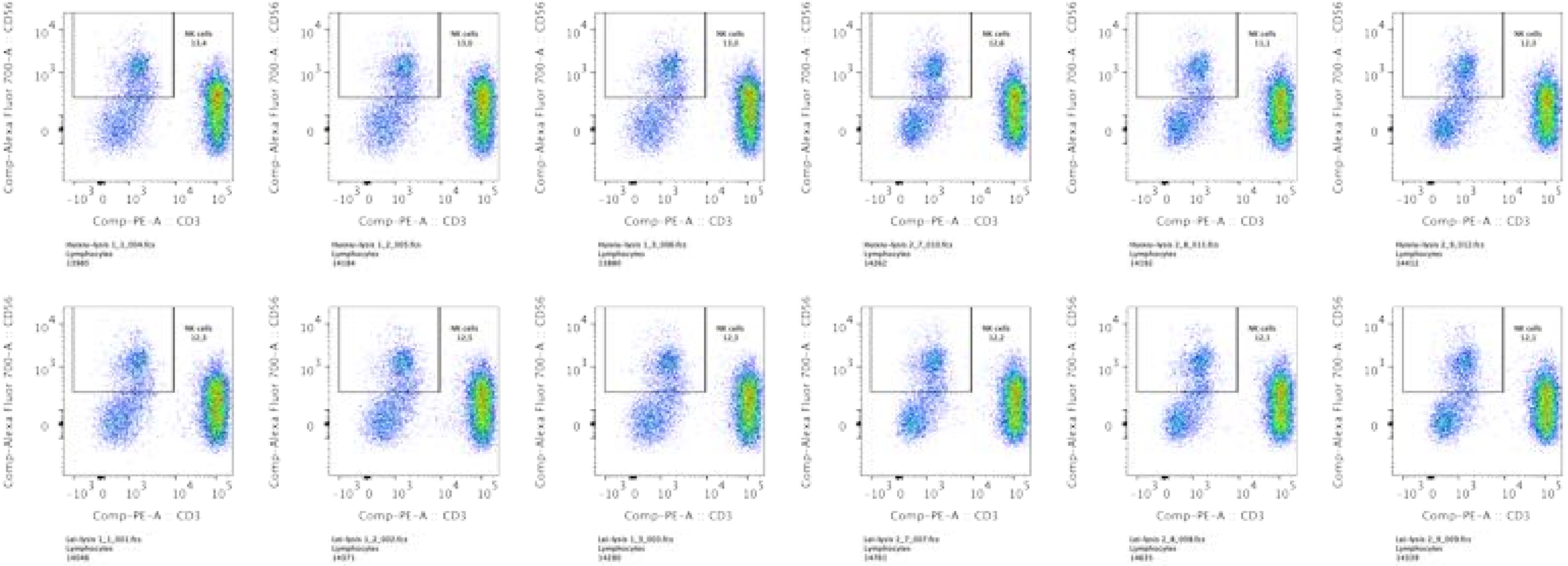

Inter-operator

	1x Lysing	2x Lysing
Operator 1		
Operator 2		

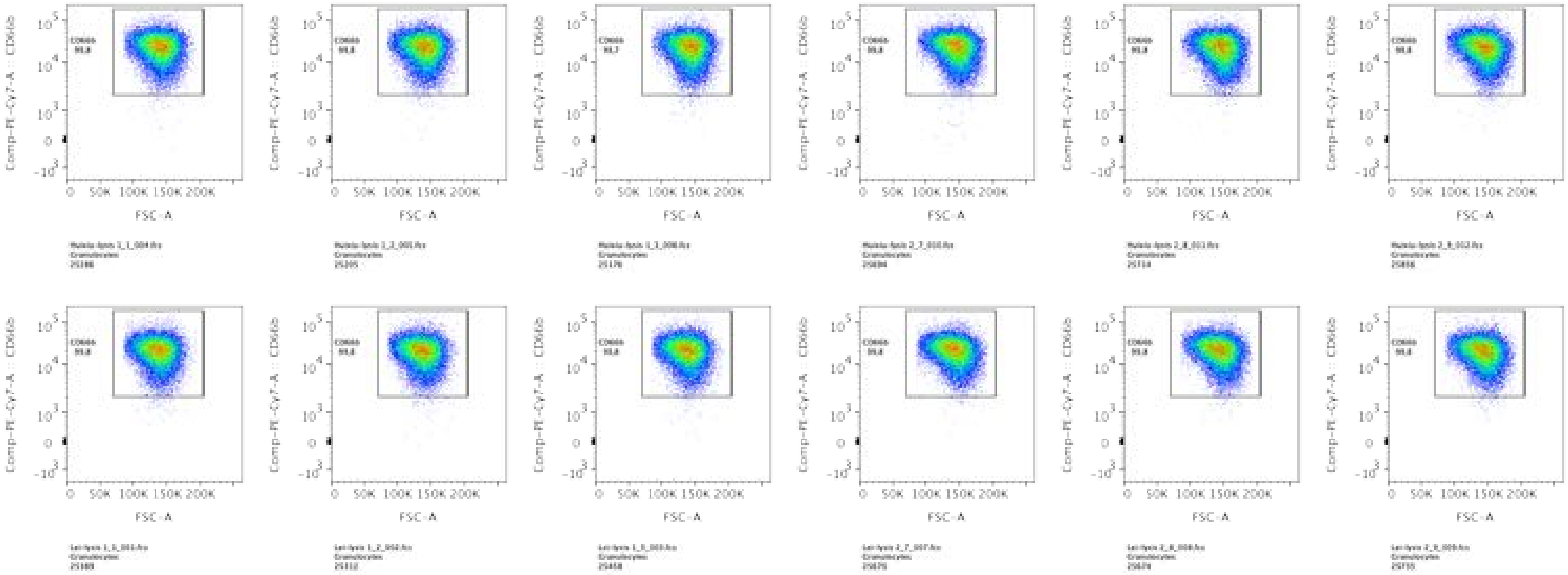

Sample stability over time

0h

6h

24h

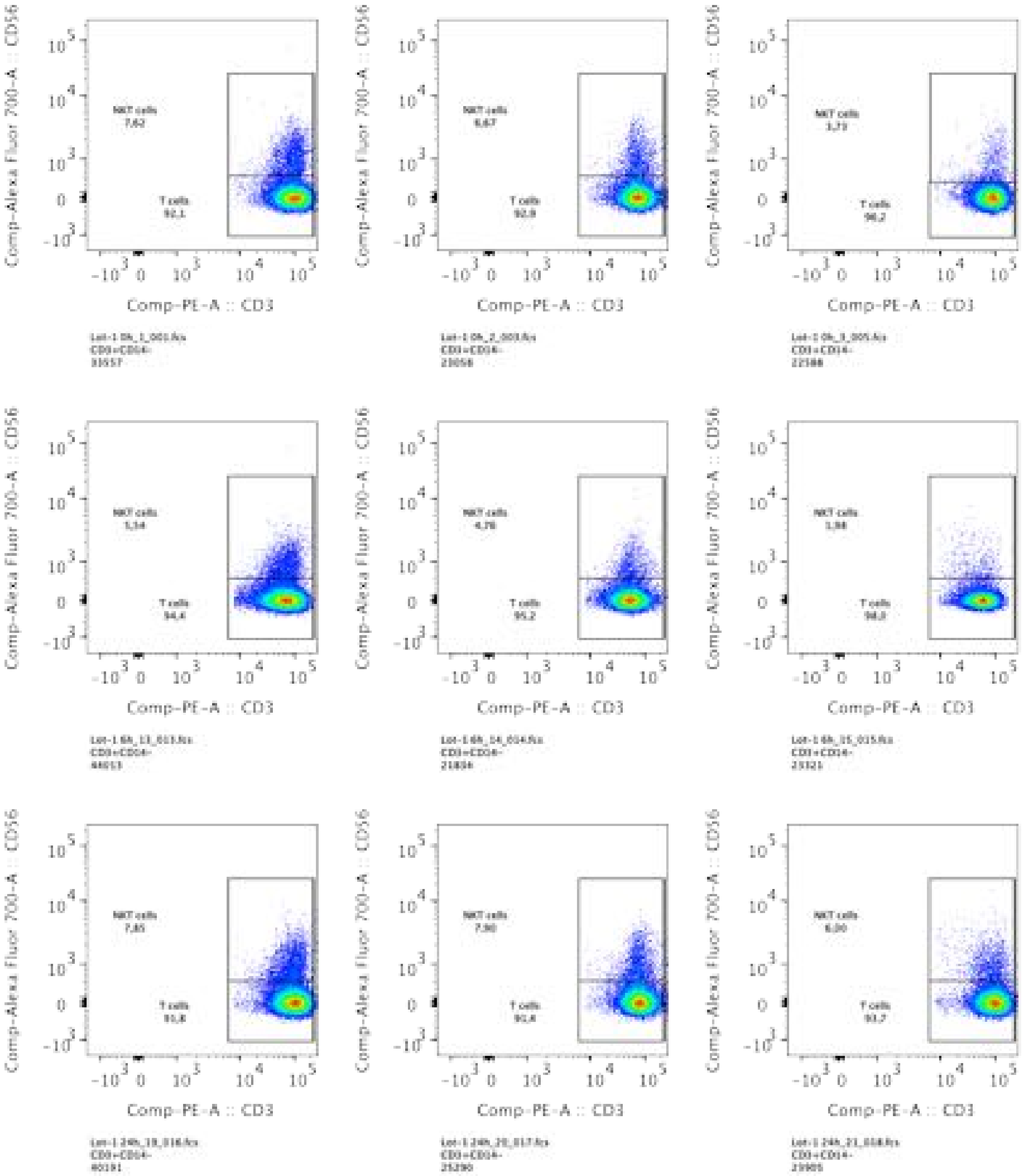

Sample stability over time

0h

6h

24h

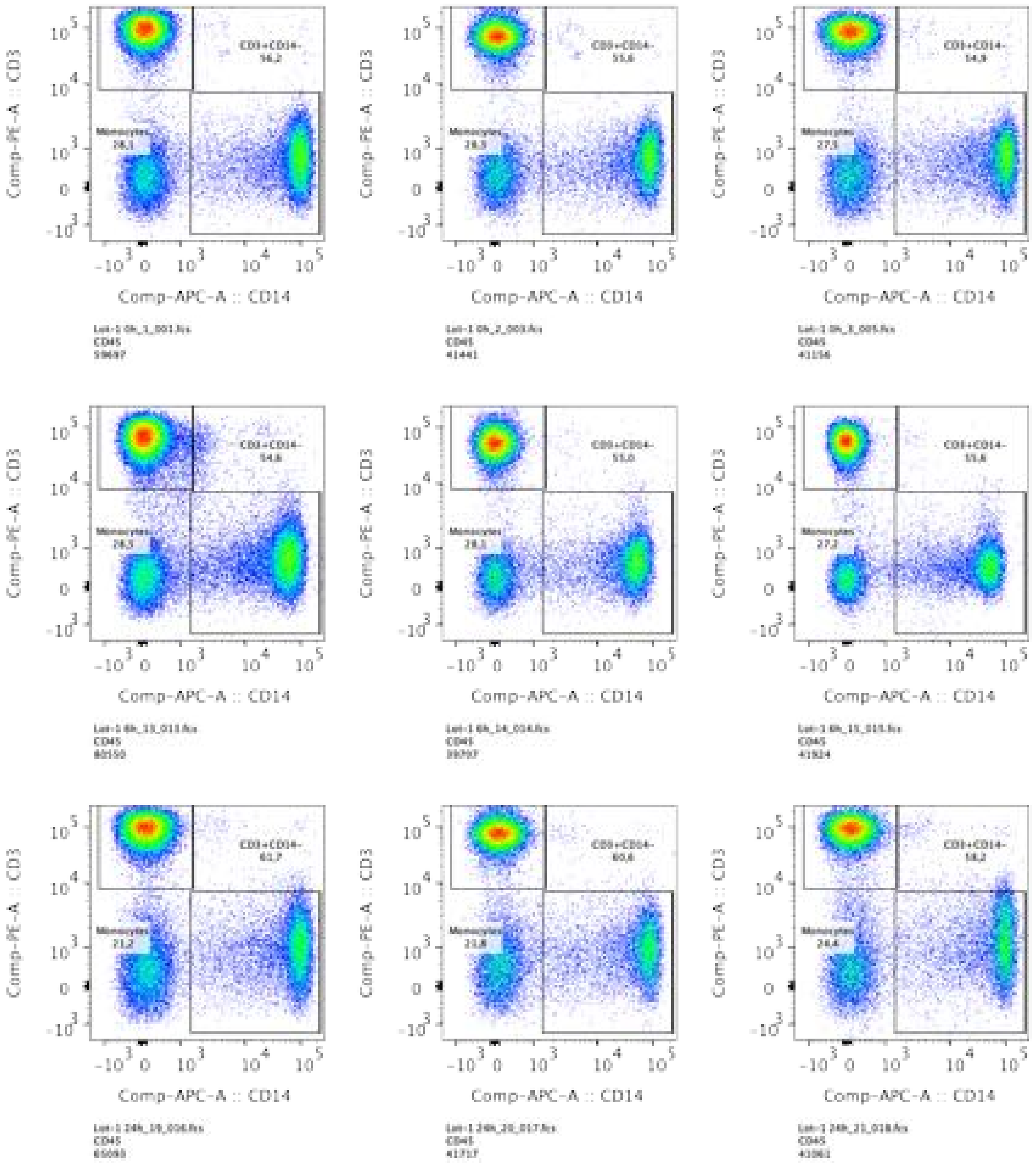

Sample stability over time

0h

6h

24h

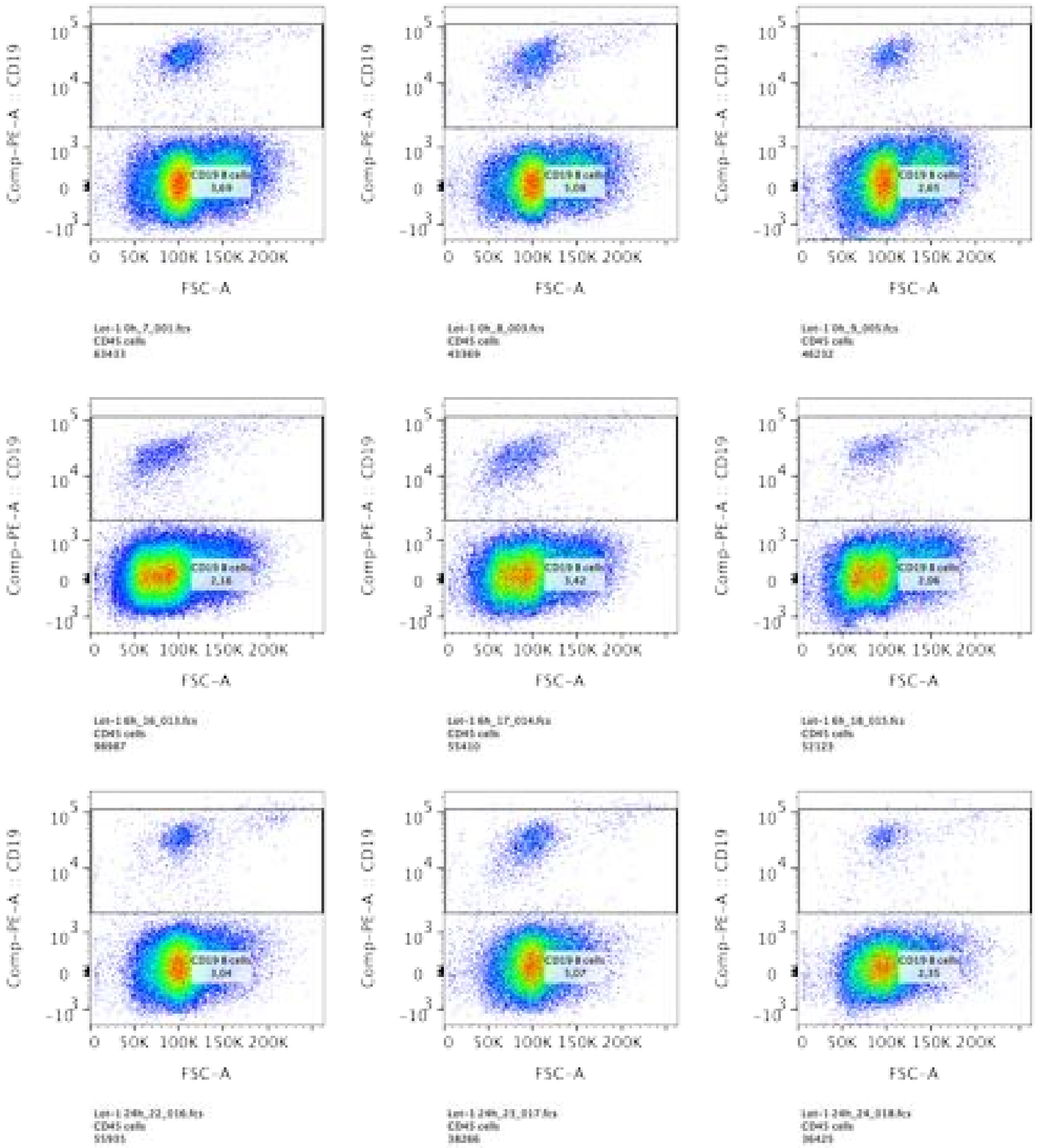

Sample stability over time

0h

6h

24h

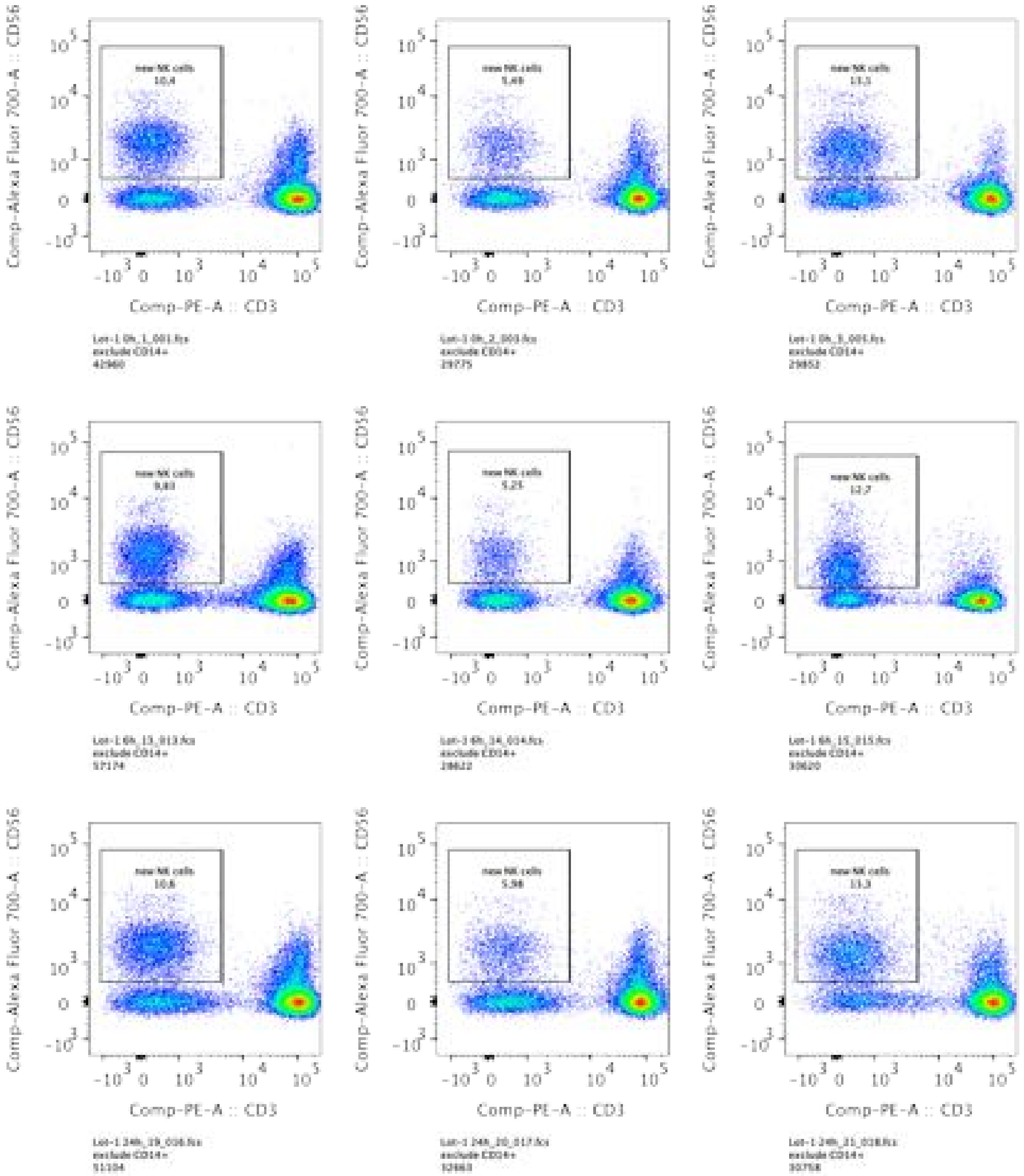

Sample stability over time

0h

6h

24h

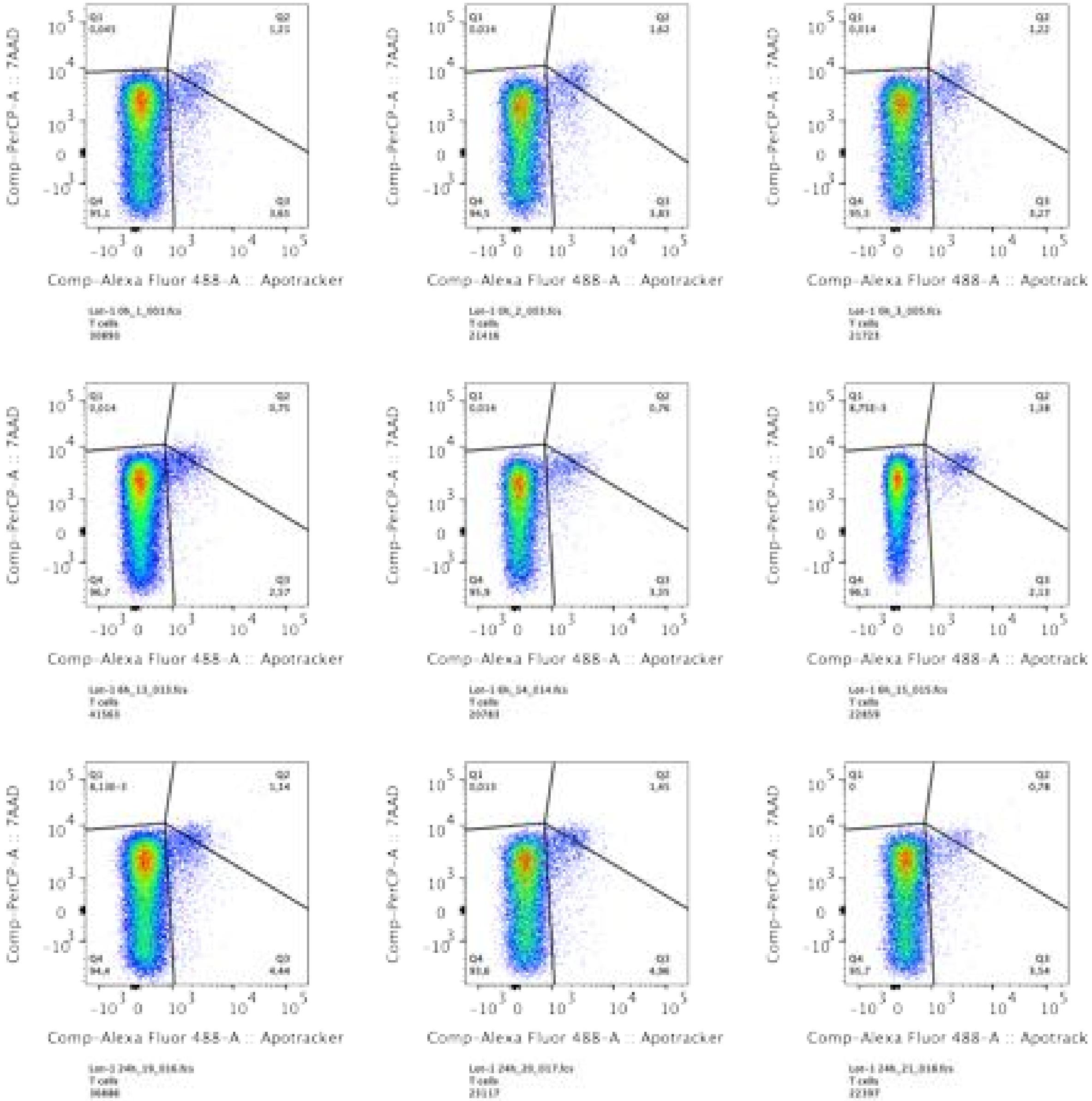

Sample stability over time

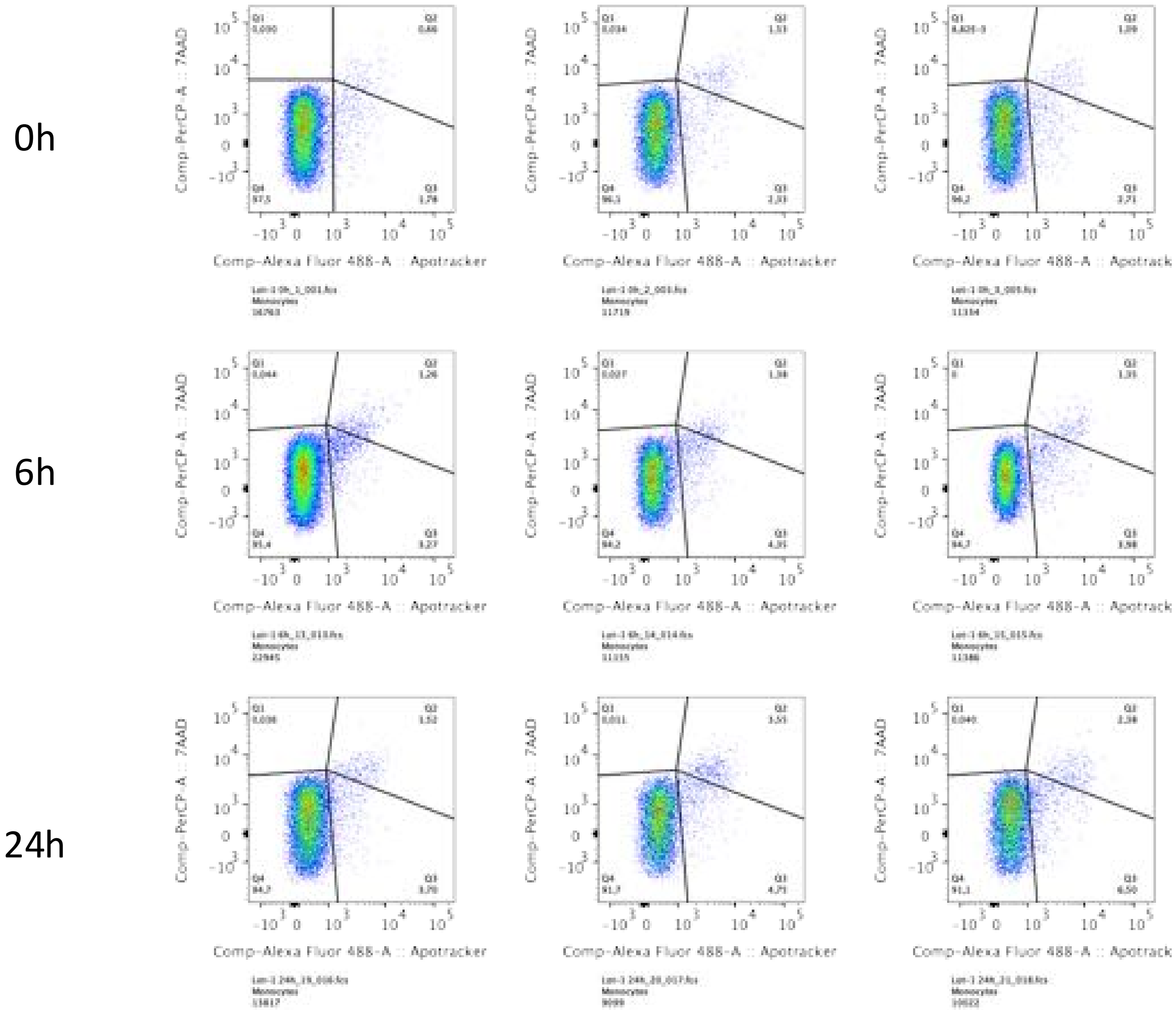

Sample stability over time

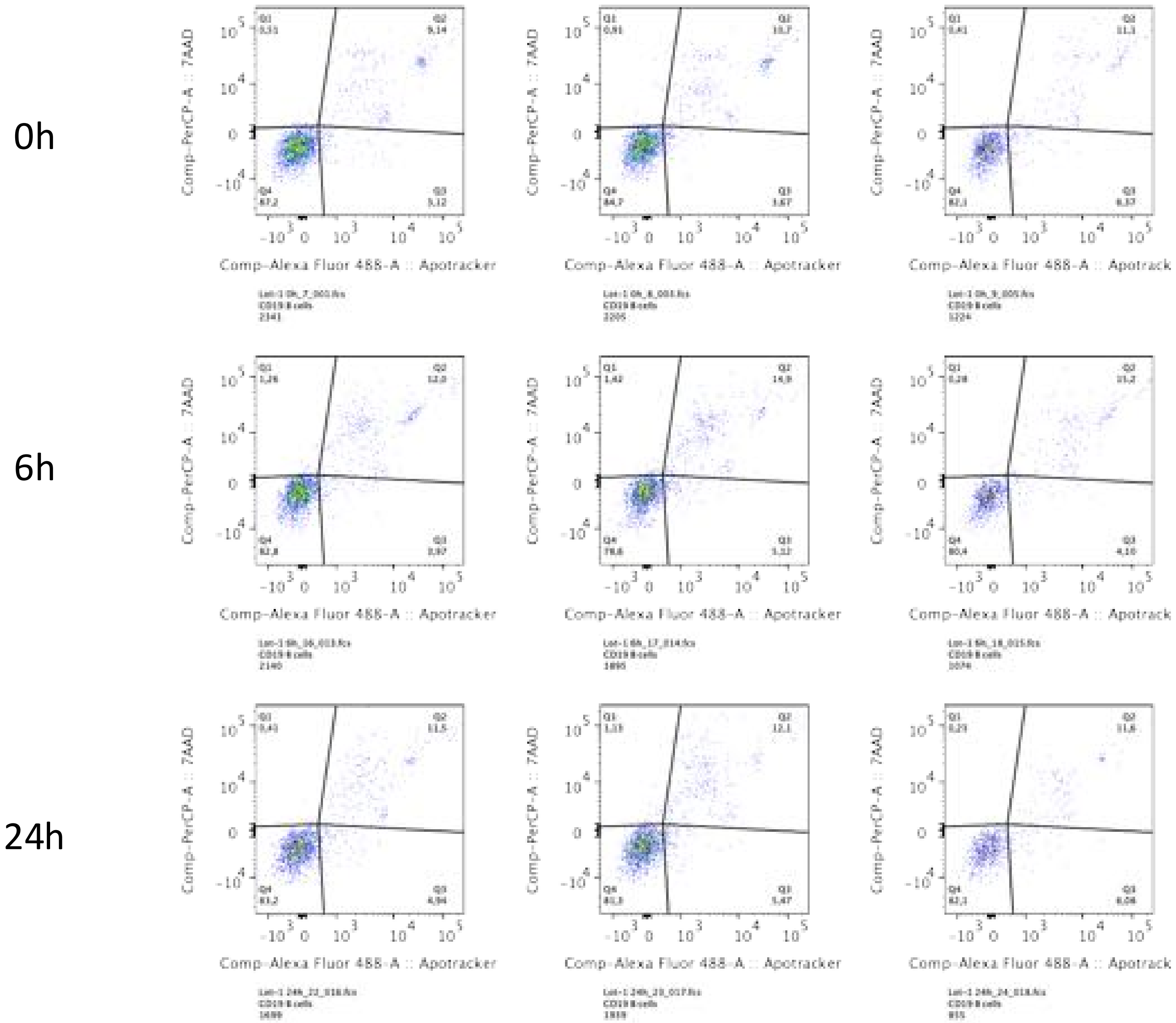

Sample stability over time

0h

6h

24h

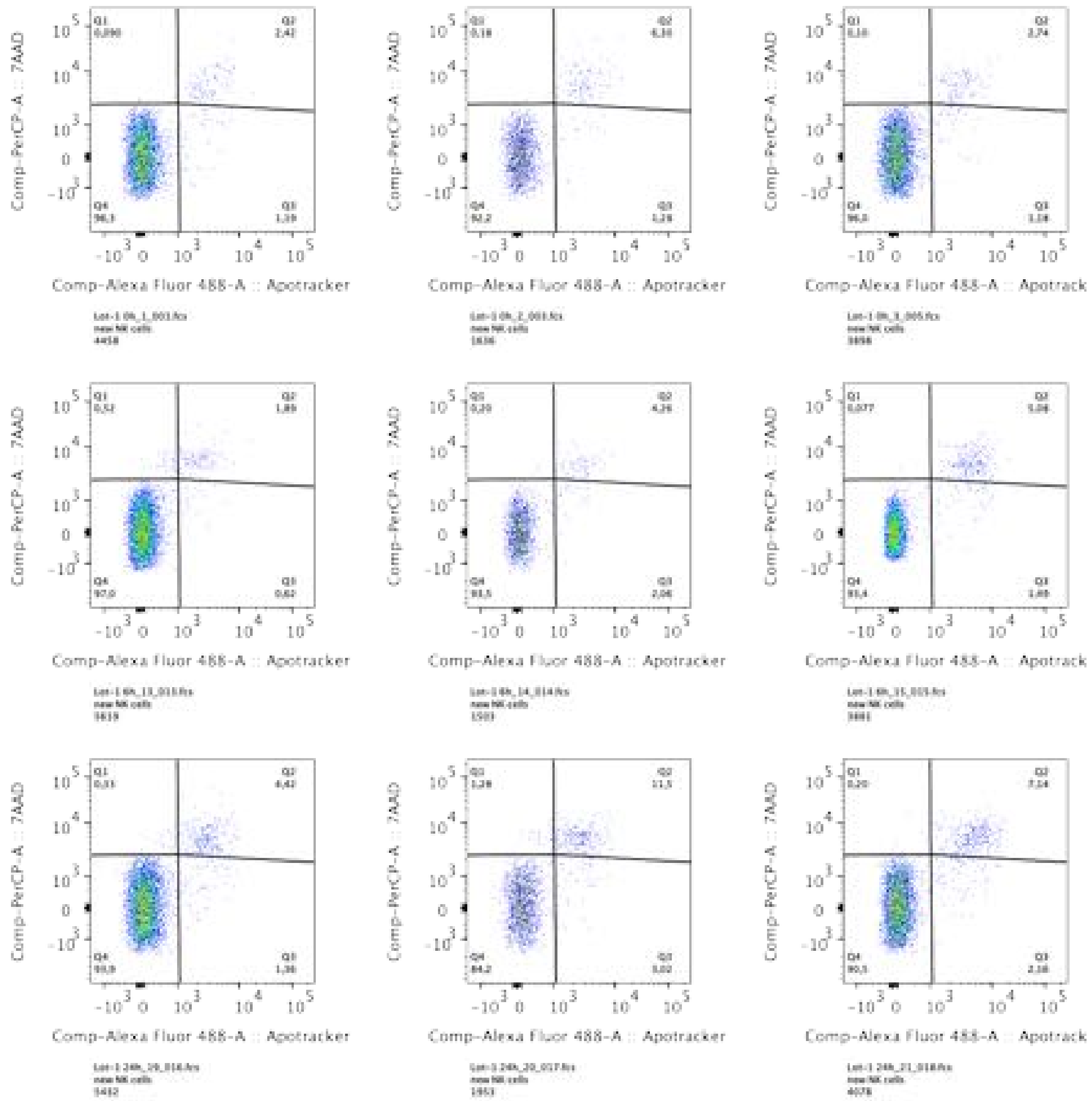

Sample stability over time

0h

6h

24h

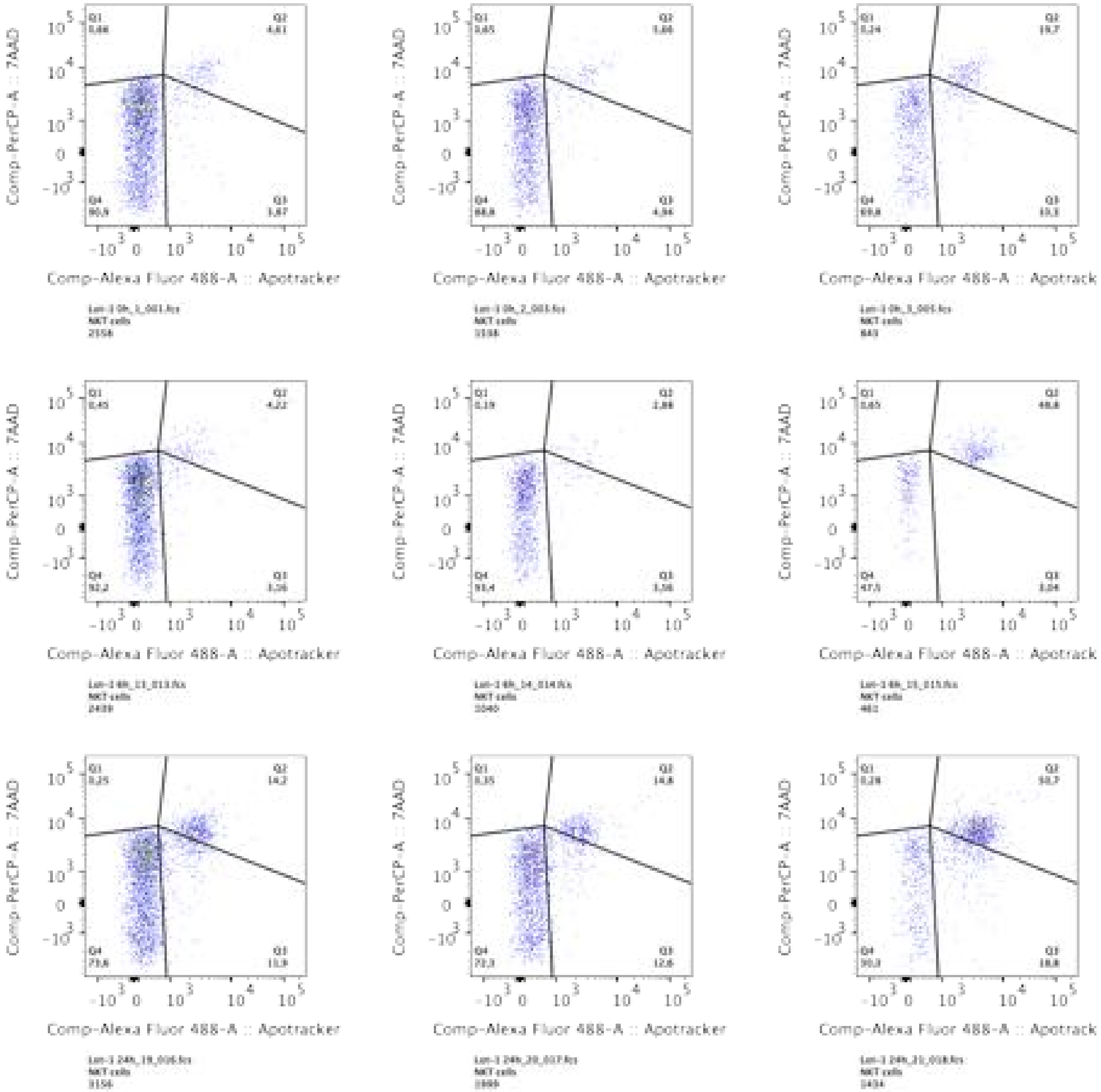

Sample stability over time

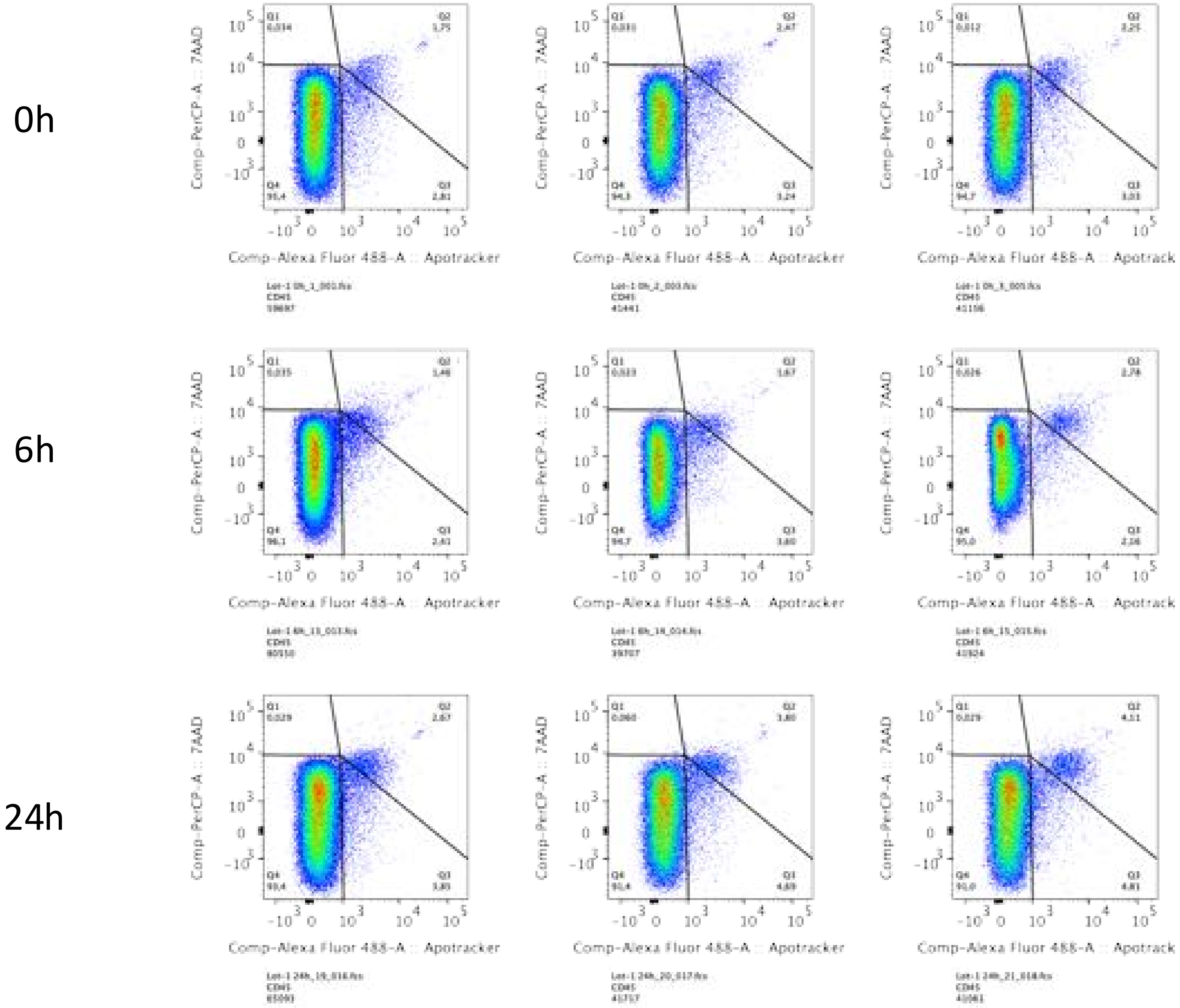

Cocktail stability day1

Cocktail stability day2

Sample1

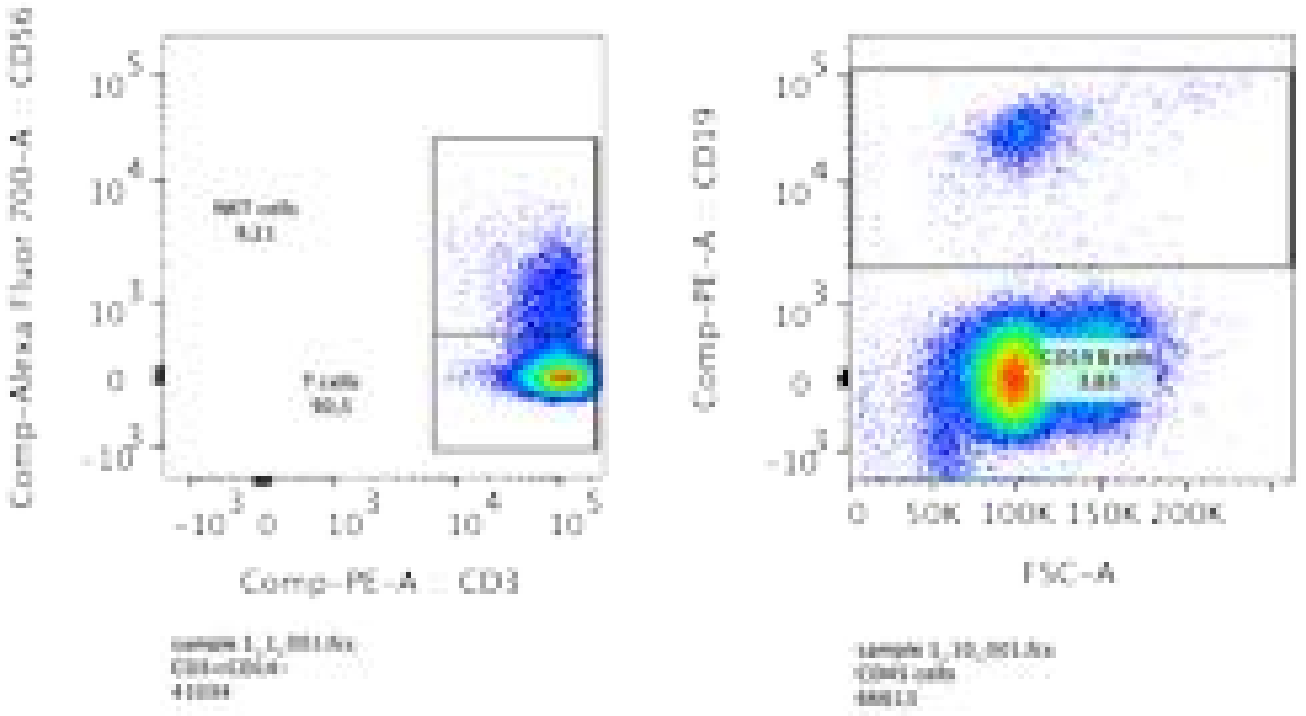

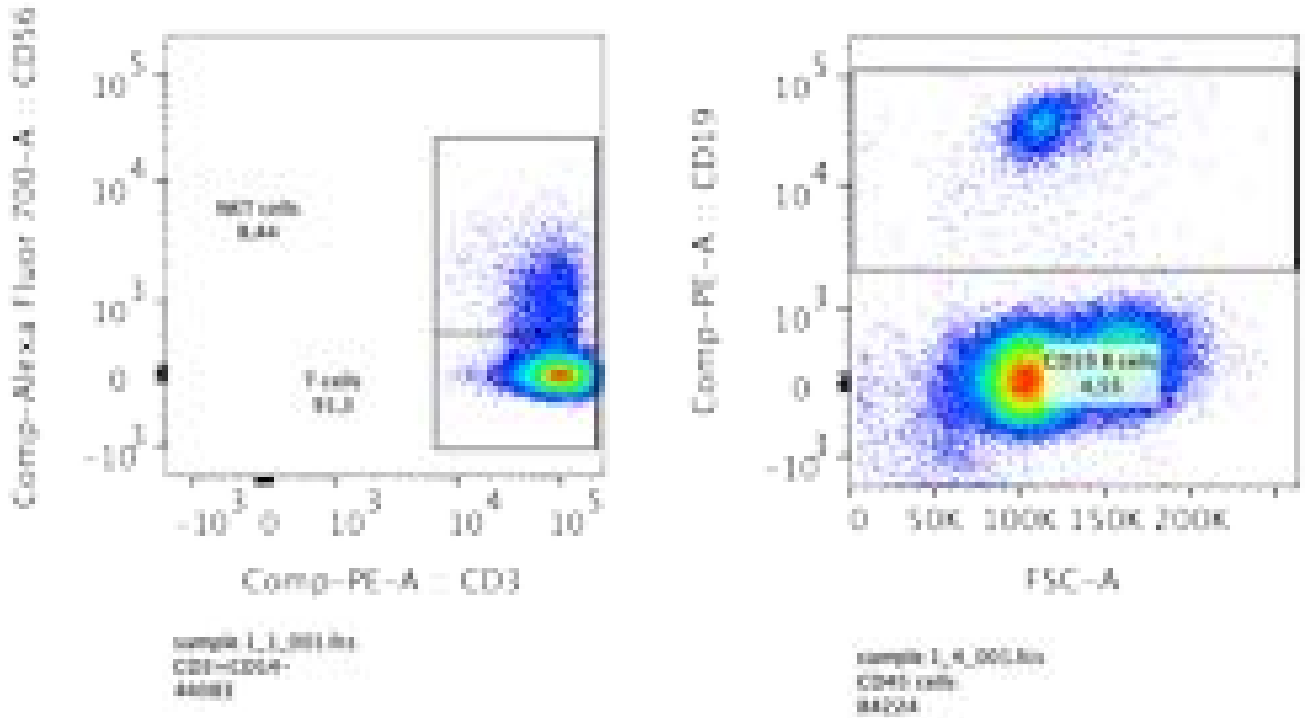

Sample2

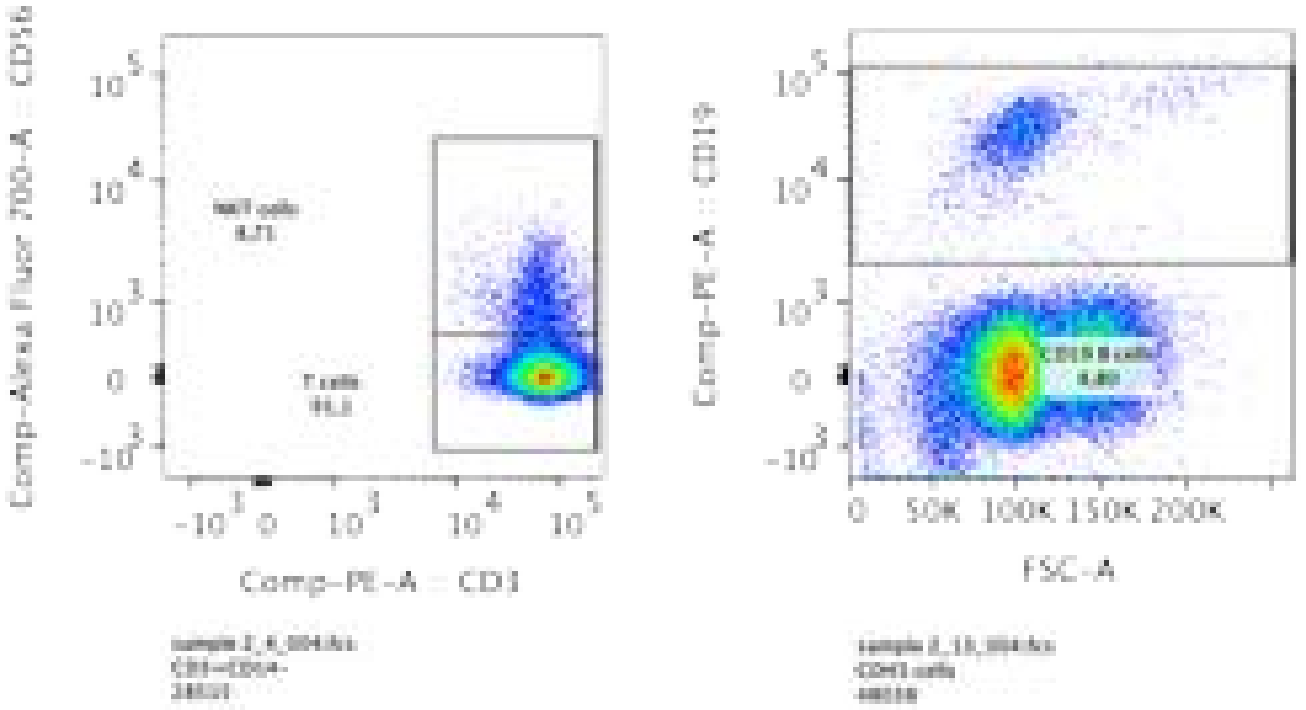

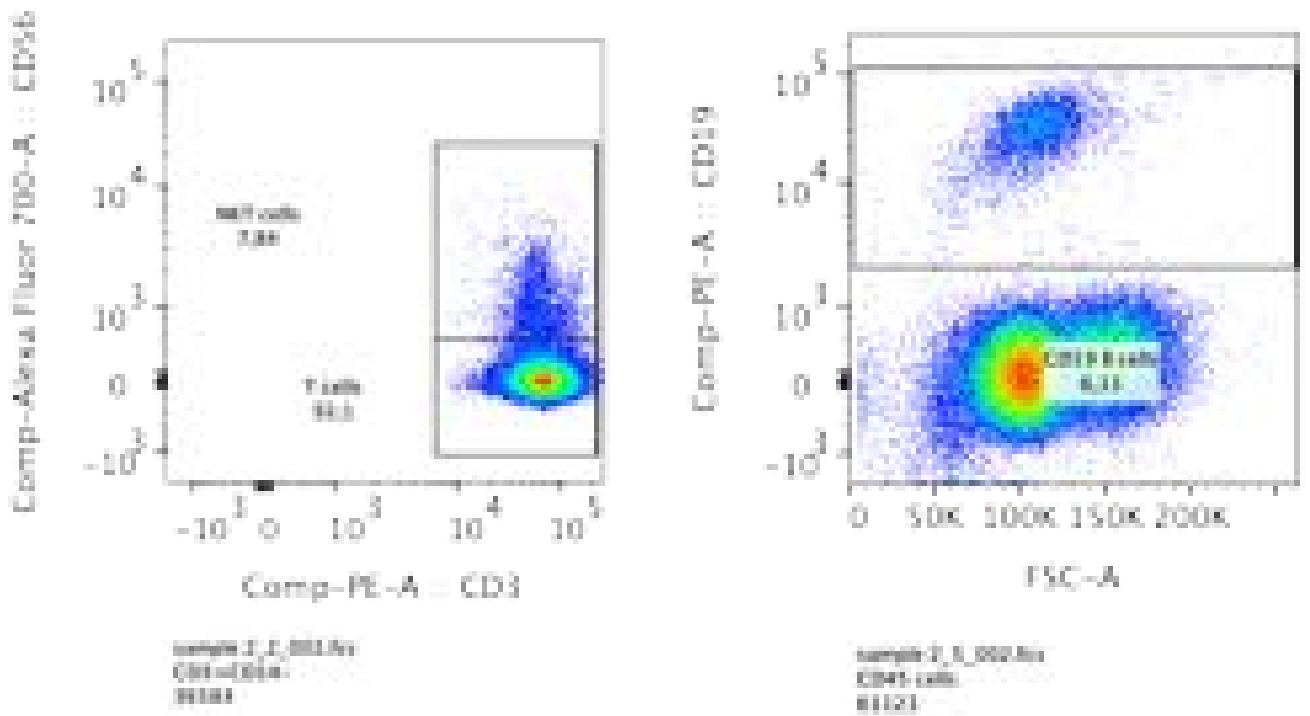

Sample3

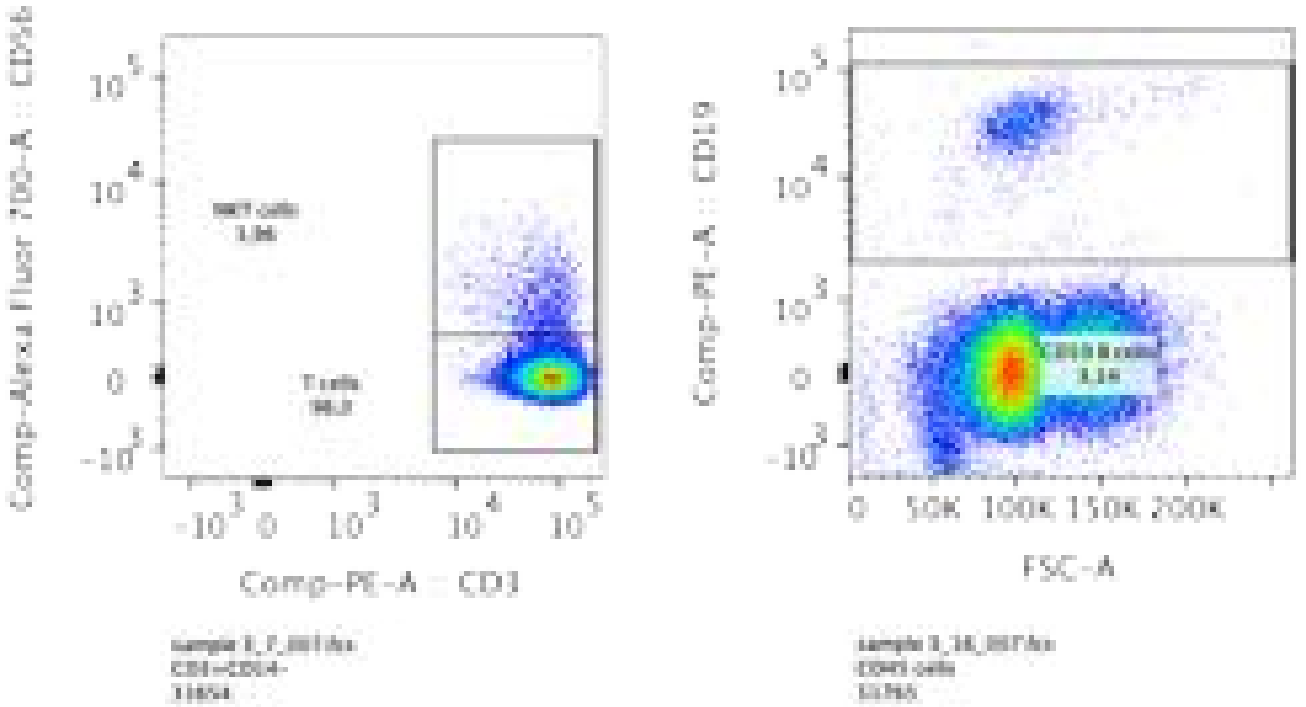

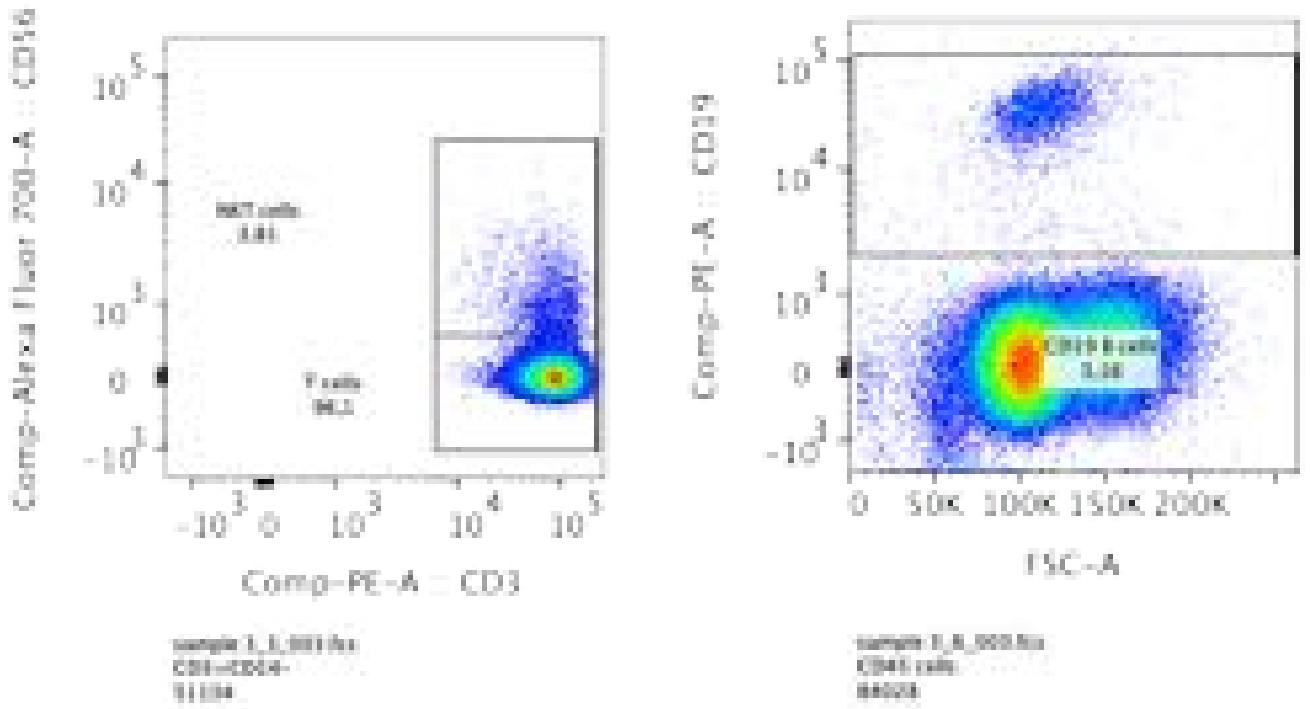

Cocktail stability day1

Cocktail stability day2

Sample1

Sample2

Sample3

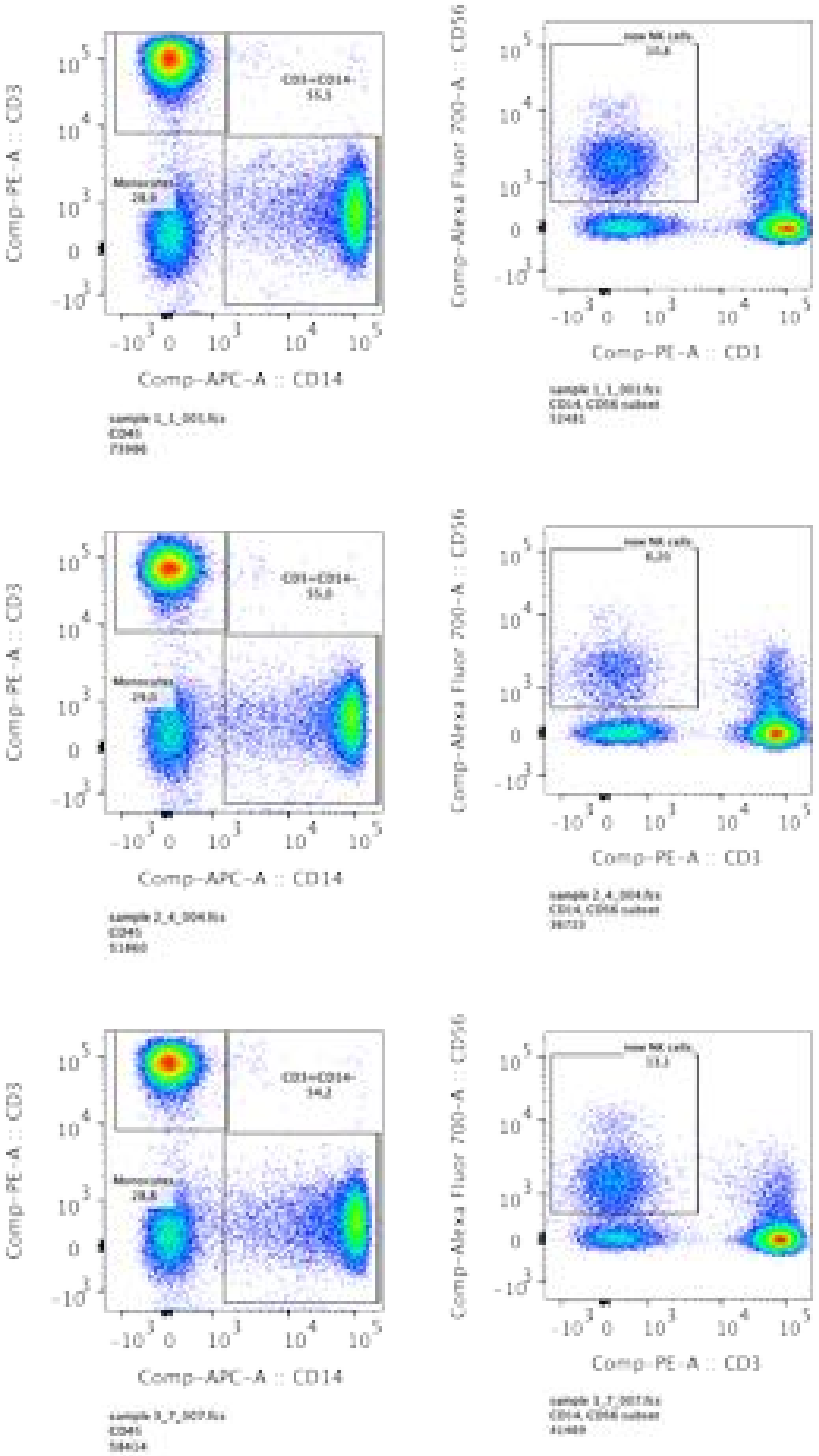

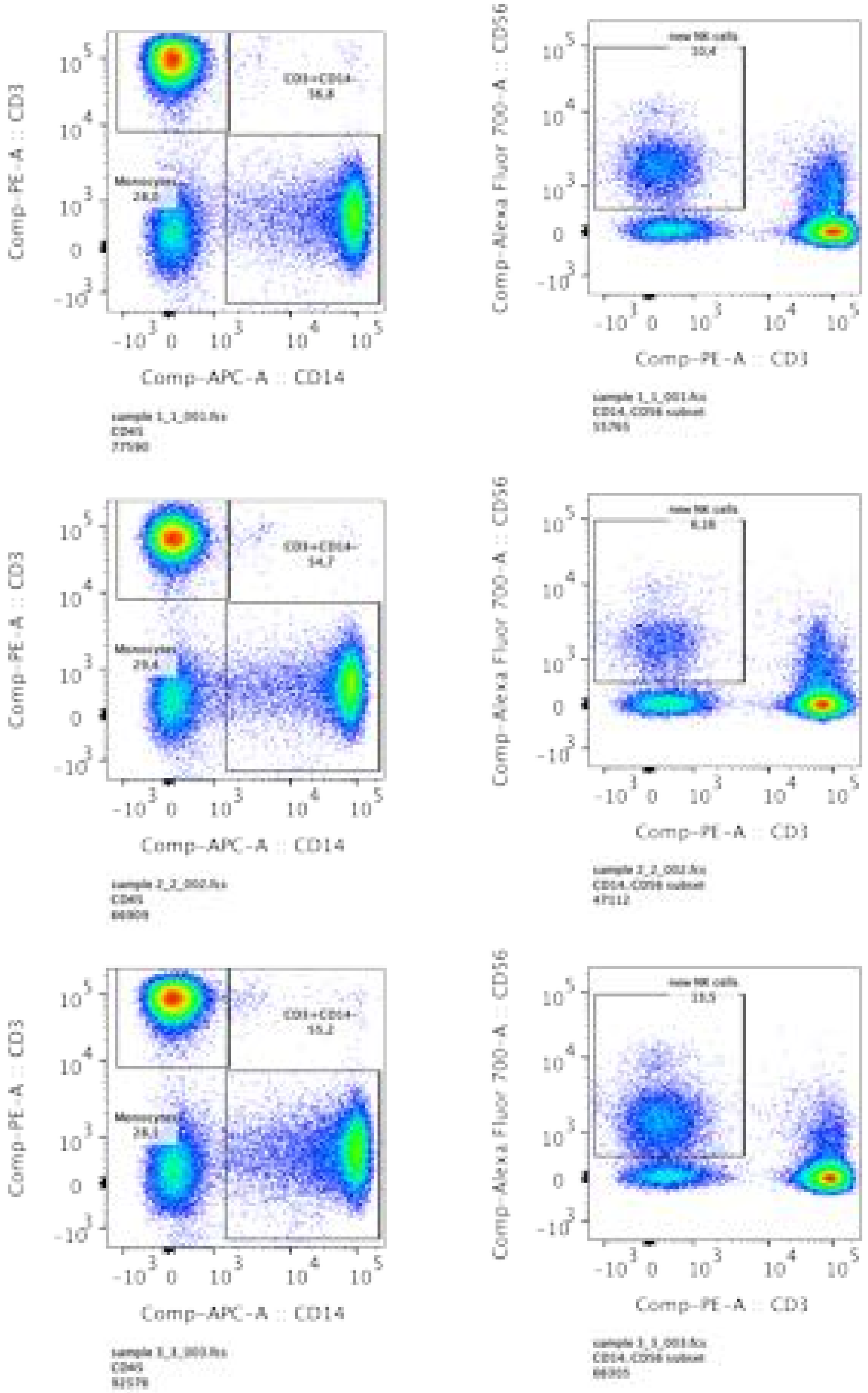

Cocktail stability day1

Cocktail stability day2

Sample1

Sample2

Sample3

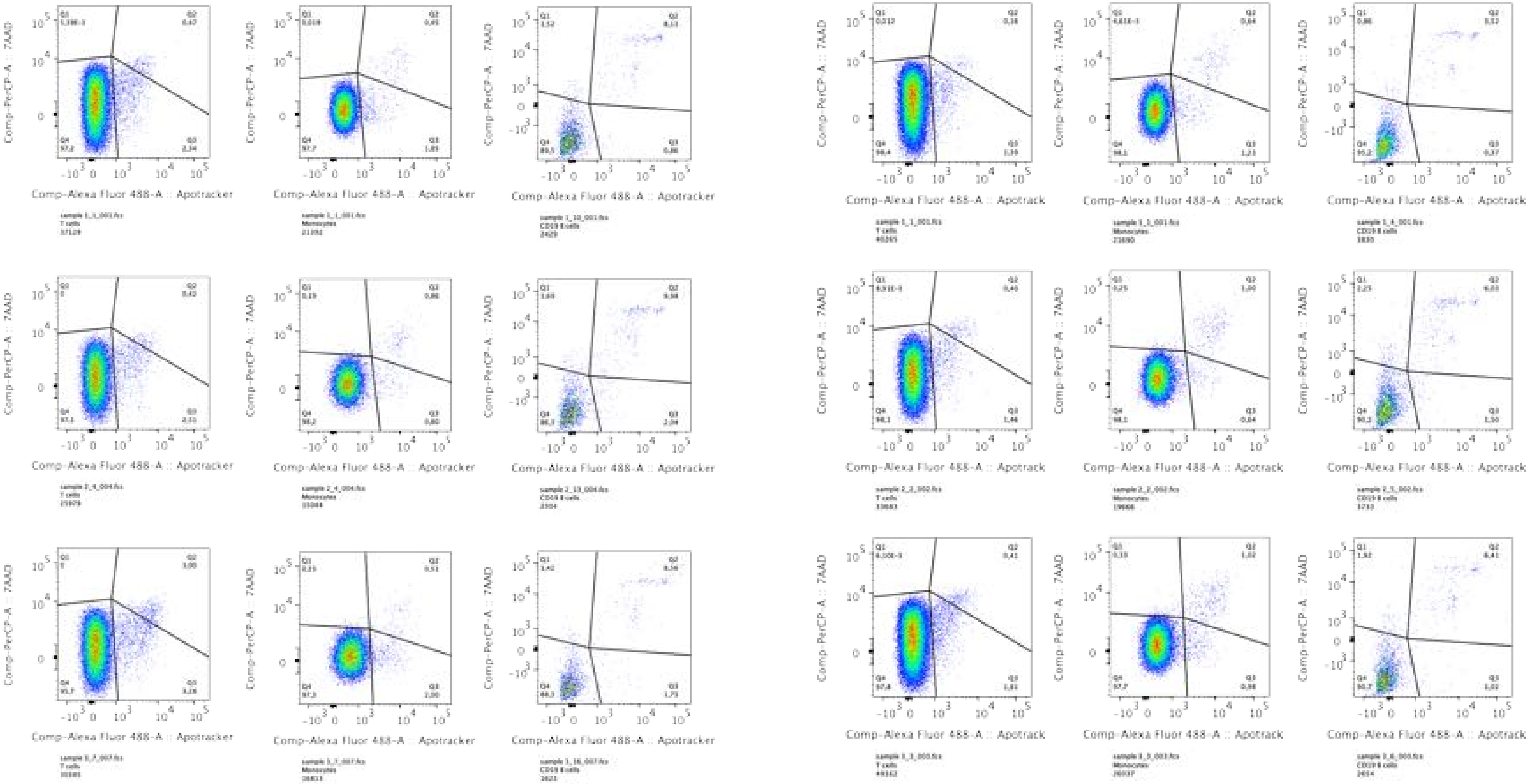

Cocktail stability day1

Cocktail stability day2

Sample1

Sample2

Sample3

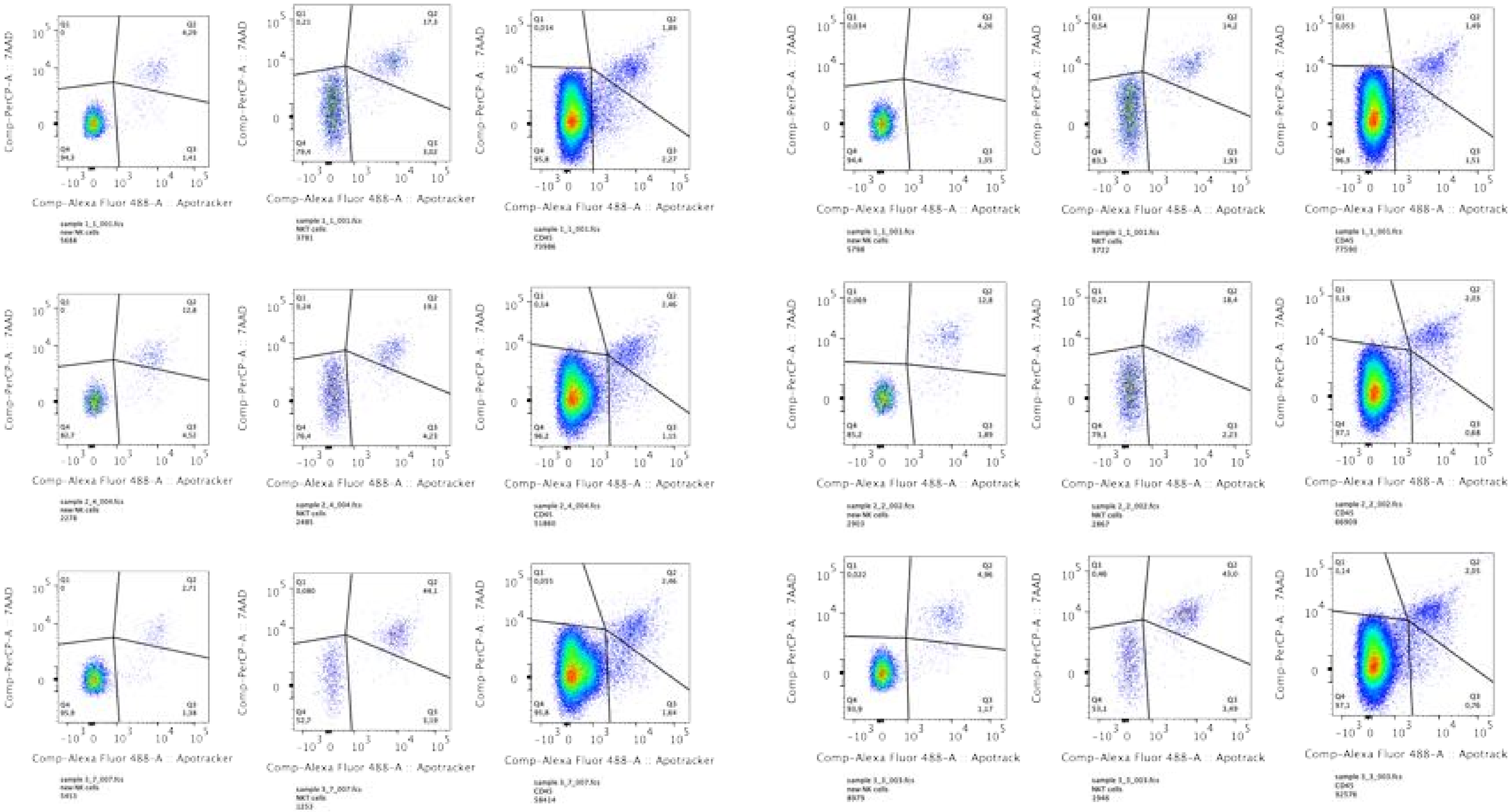

Carryover

Lot-to-lot variability

Lot 1

Lot 2

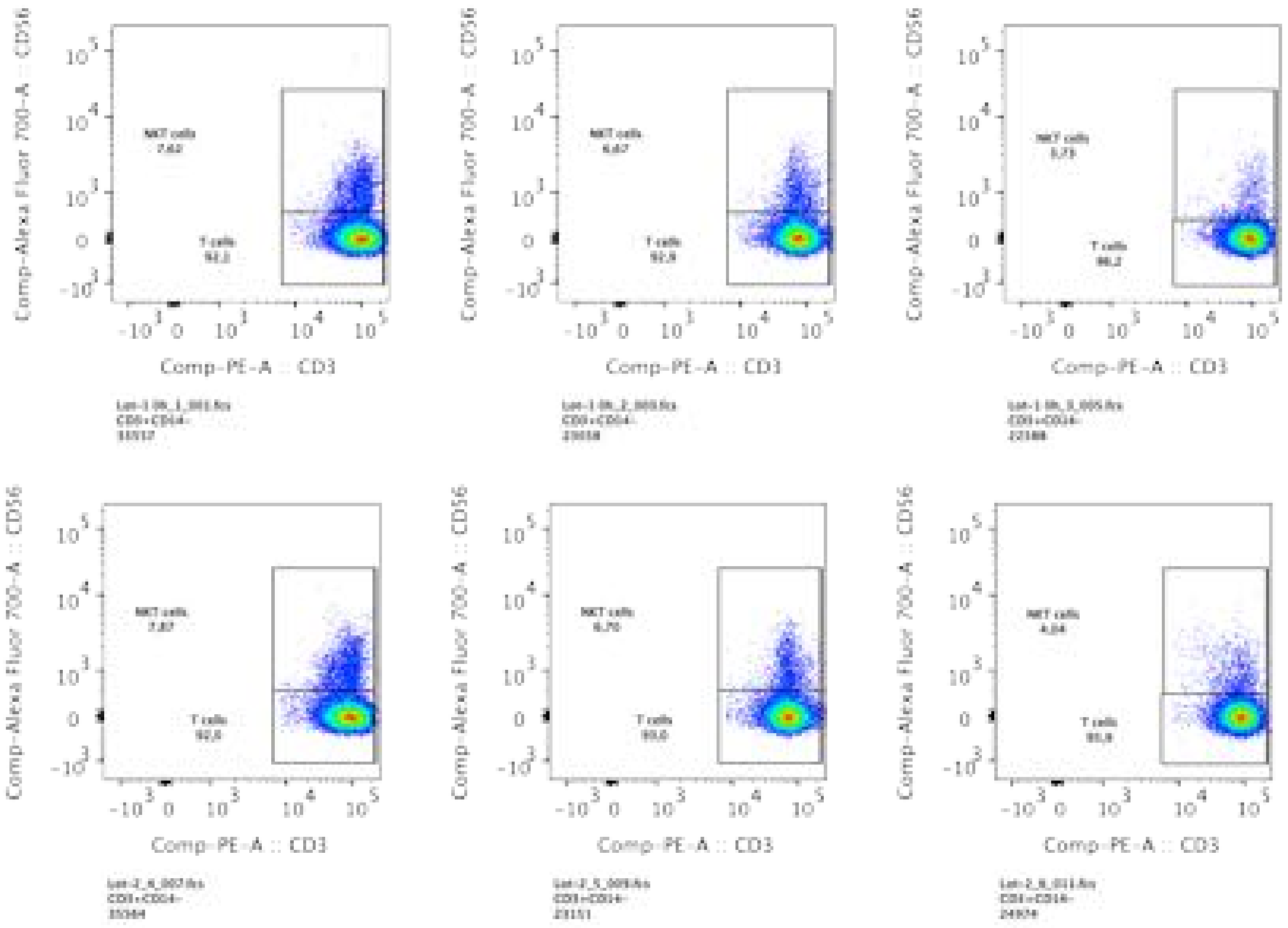

Carryover

Lot 1

Lot 2

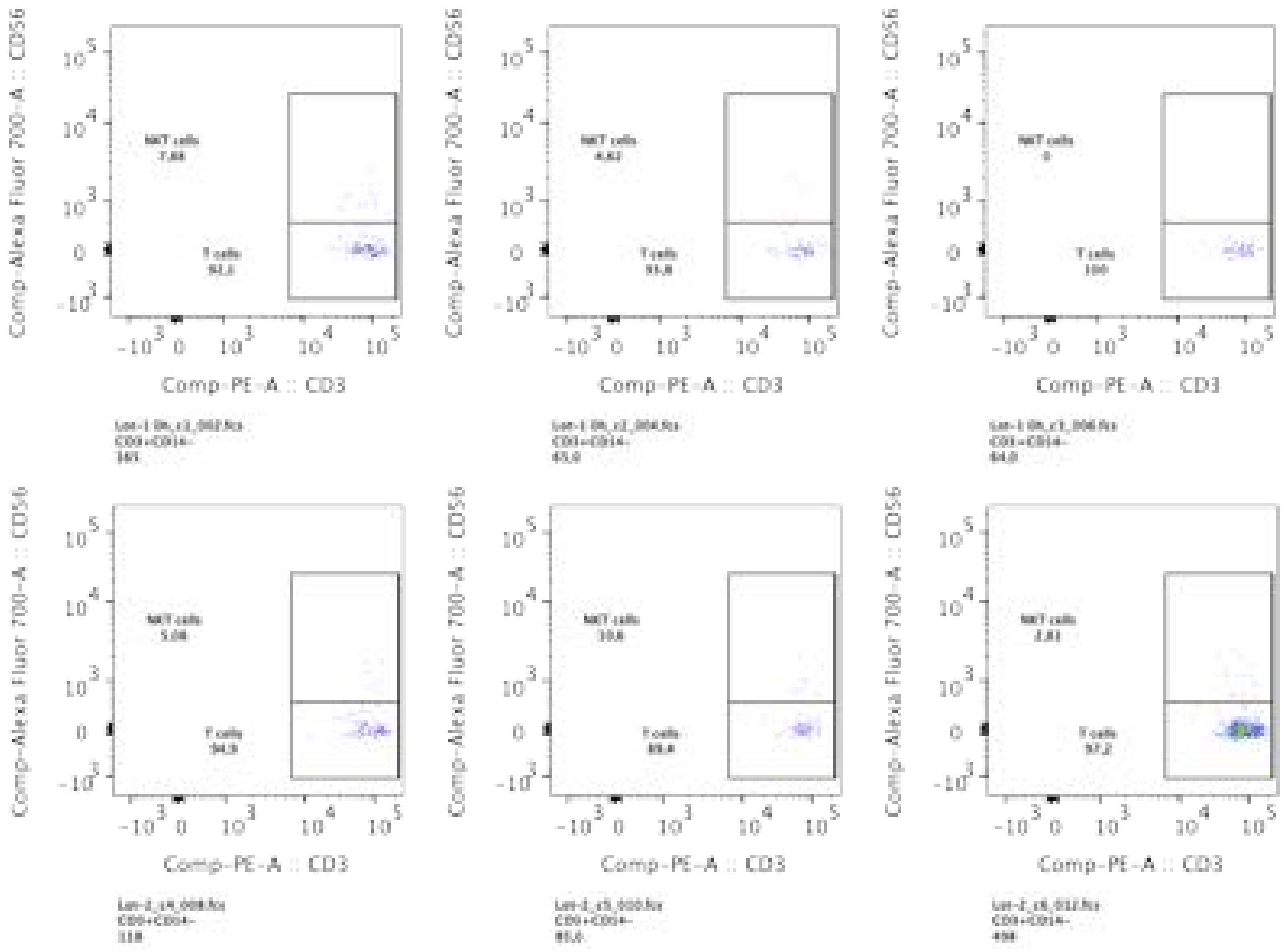

Carryover

Lot-to-lot variability

Lot 1

Lot 2

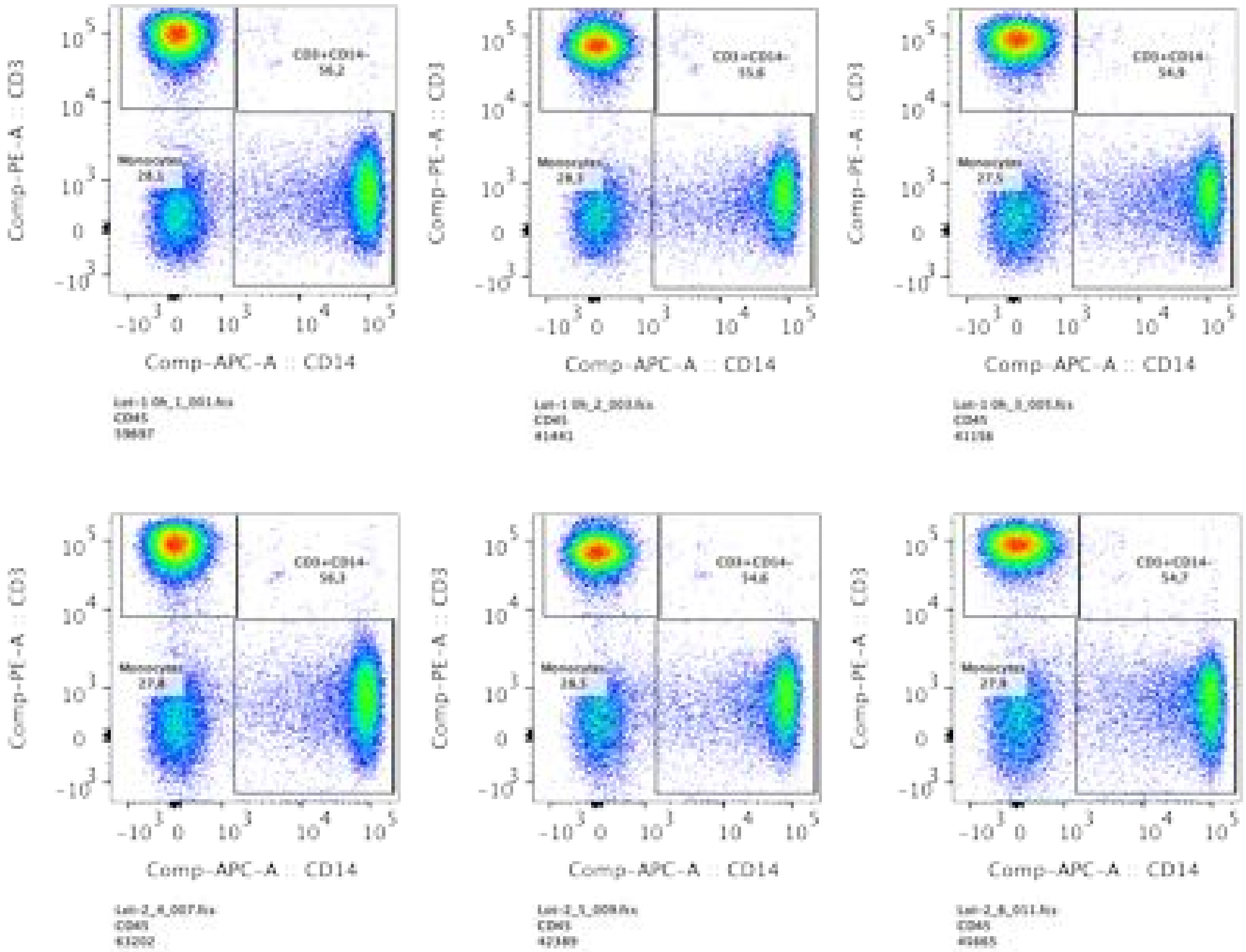

Carryover

Lot 1

Lot 2

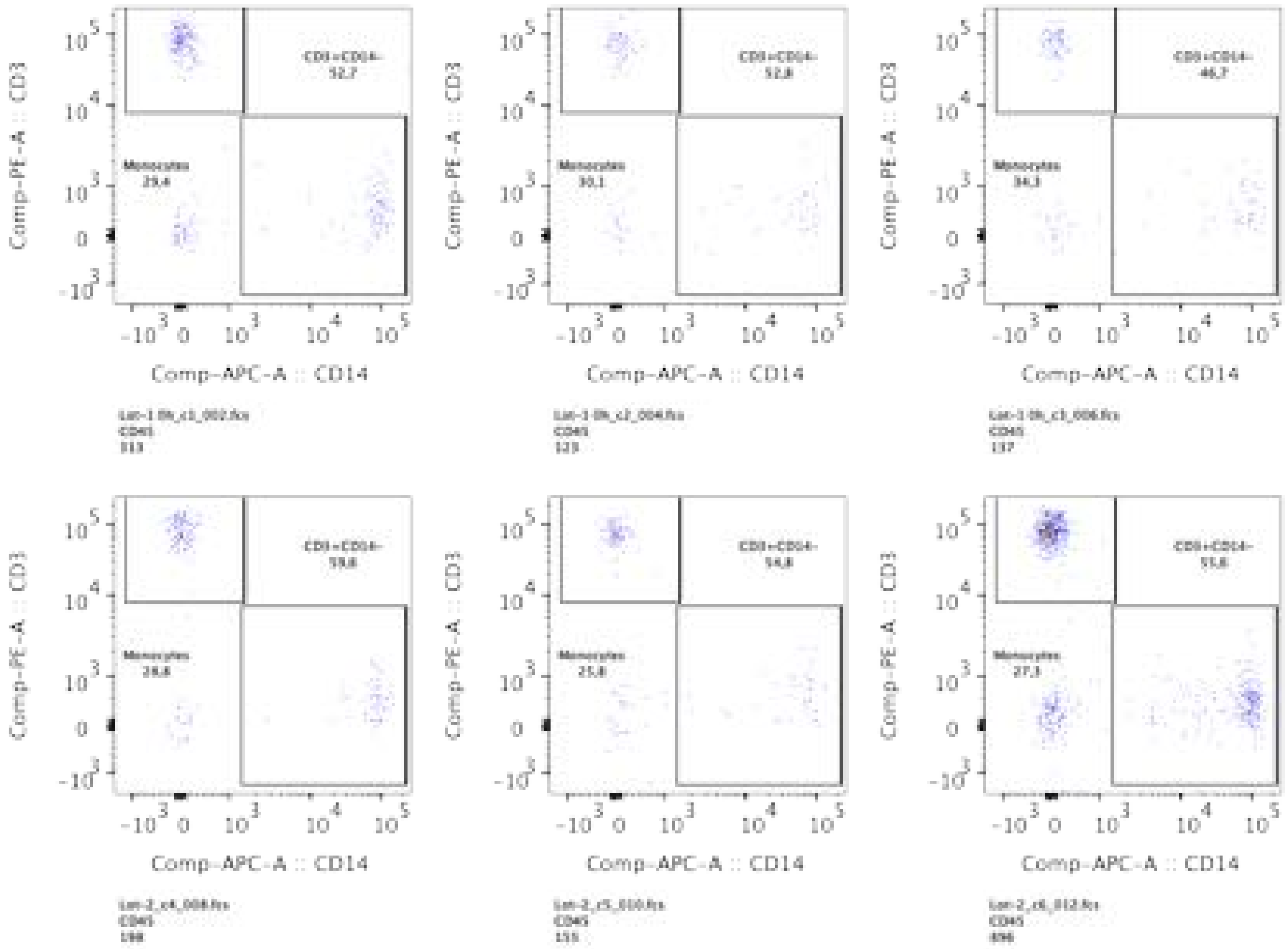

Carryover

Lot-to-lot variability

Lot 1

Lot 2

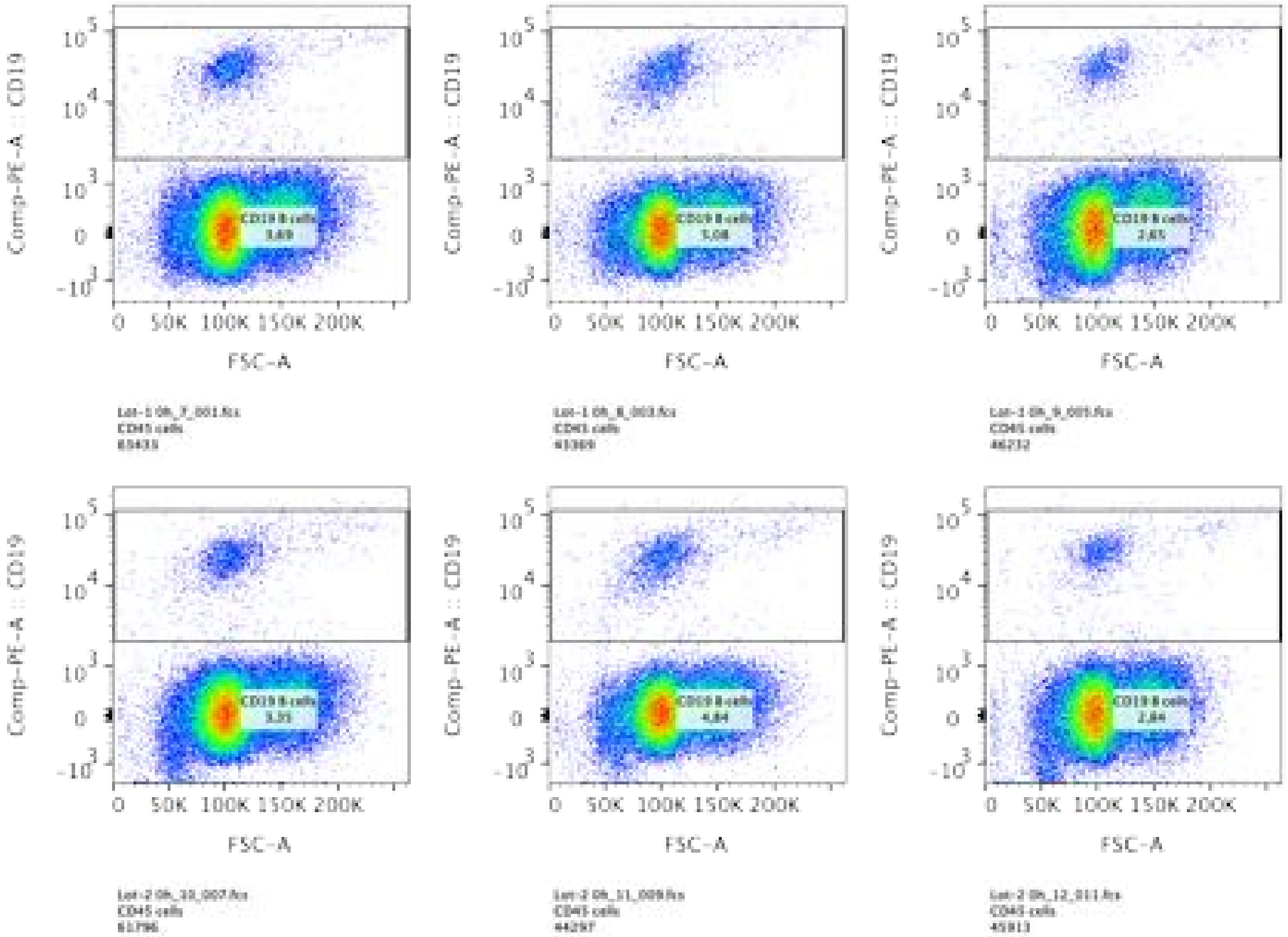

Carryover

Lot 1

Lot 2

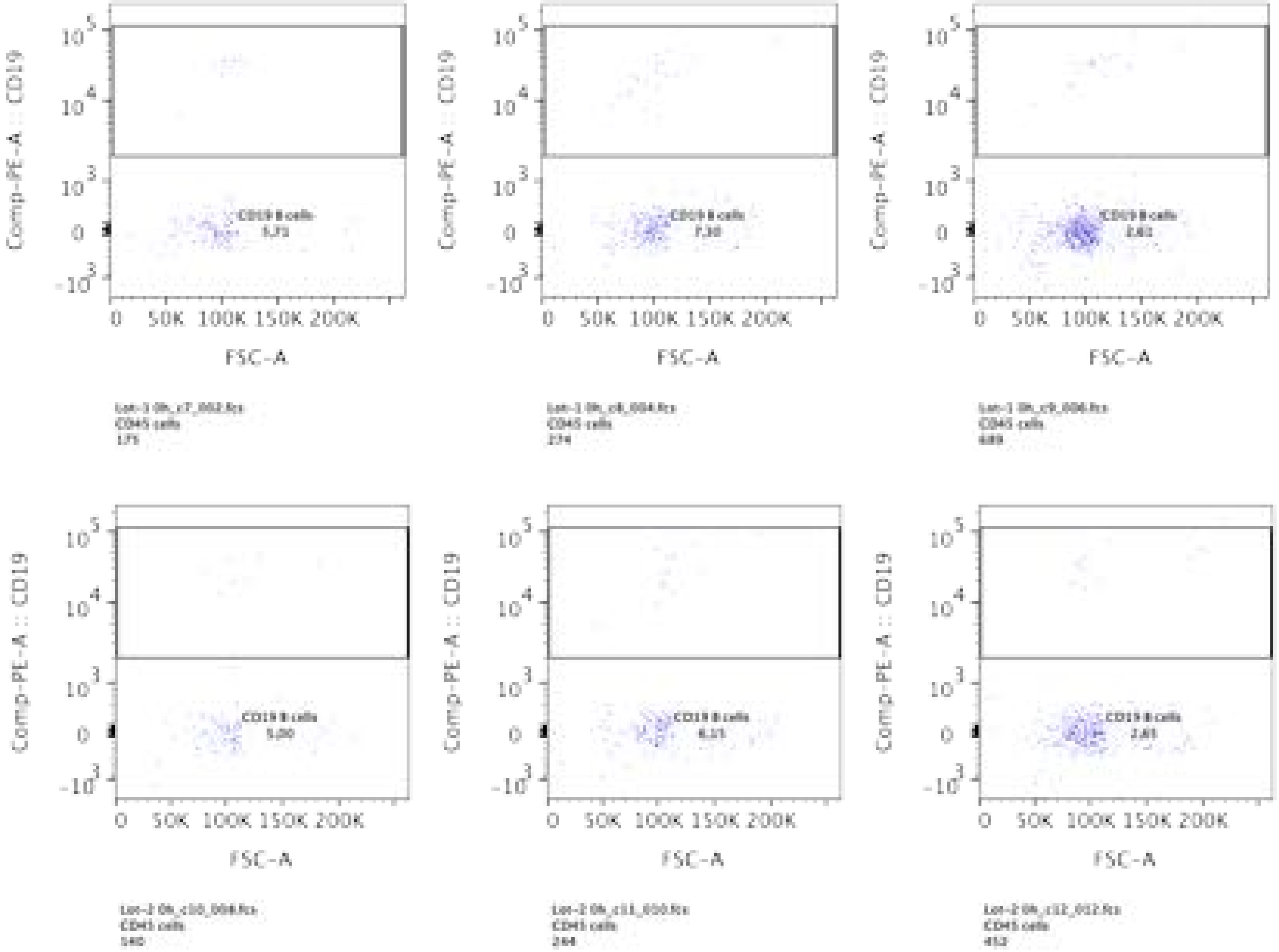

Carryover

Lot-to-lot variability

Lot 1

Lot 2

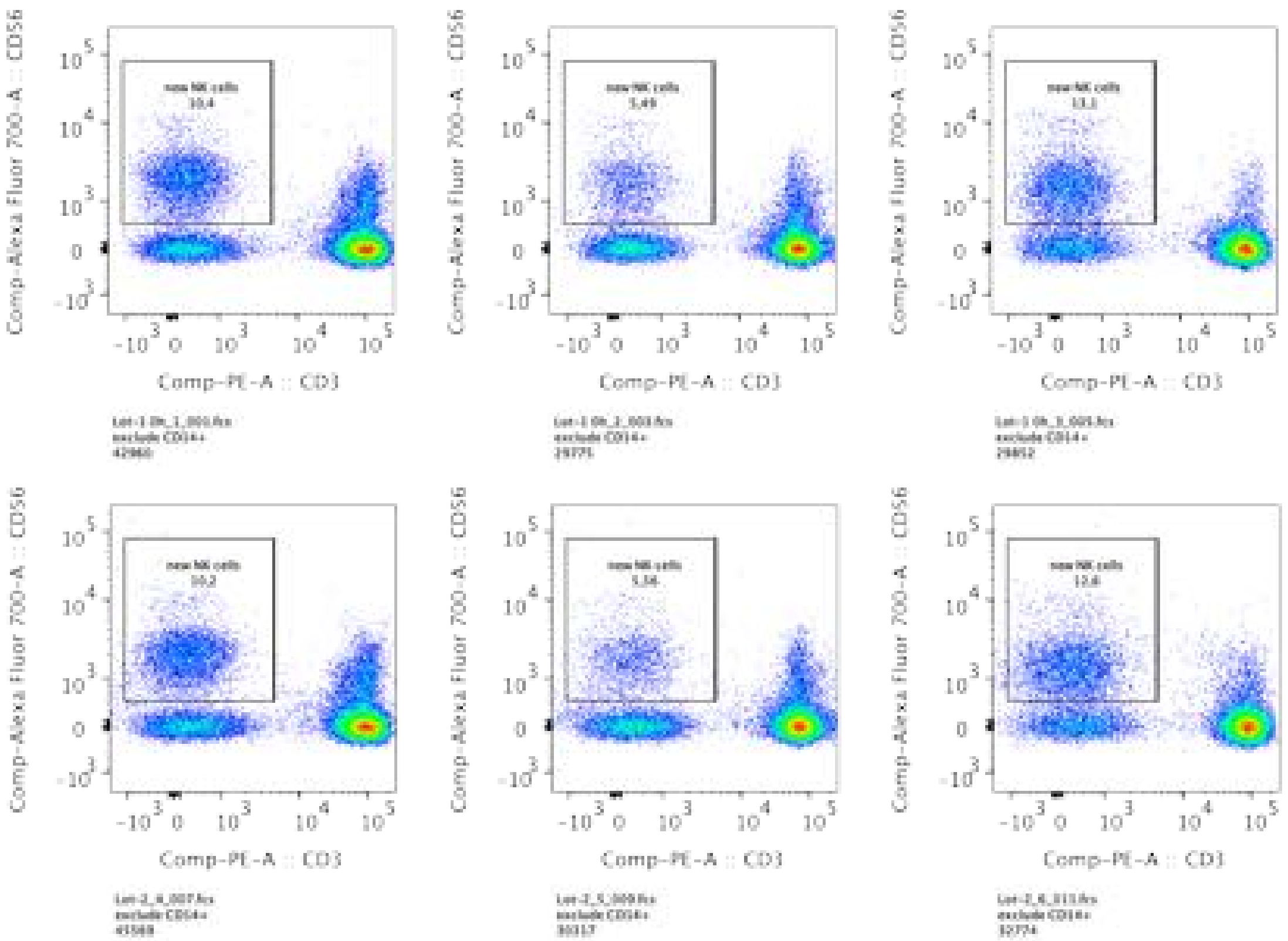

Carryover

Lot 1

Lot 2

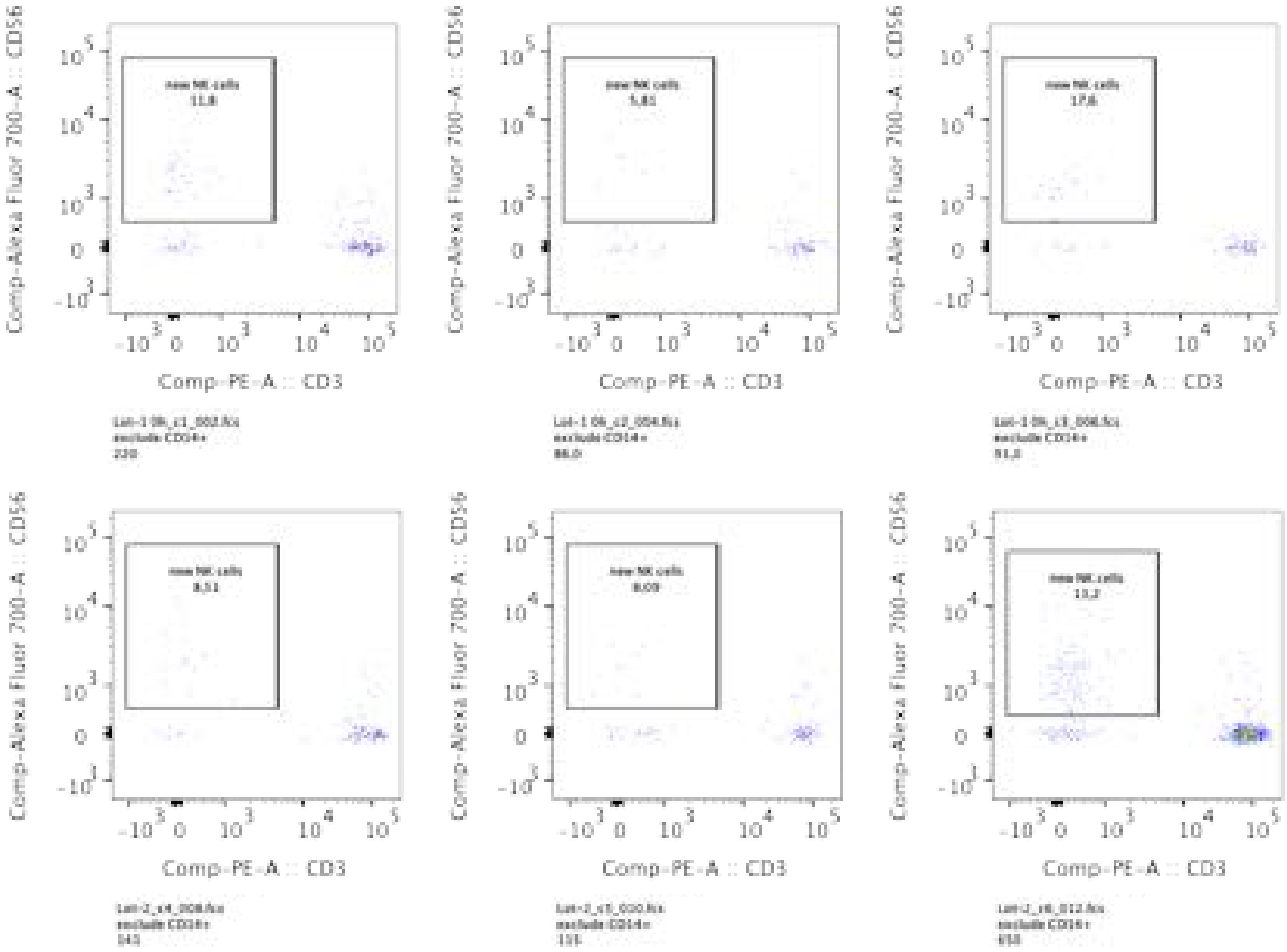

Carryover

Lot-to-lot variability

Lot 1

Lot 2

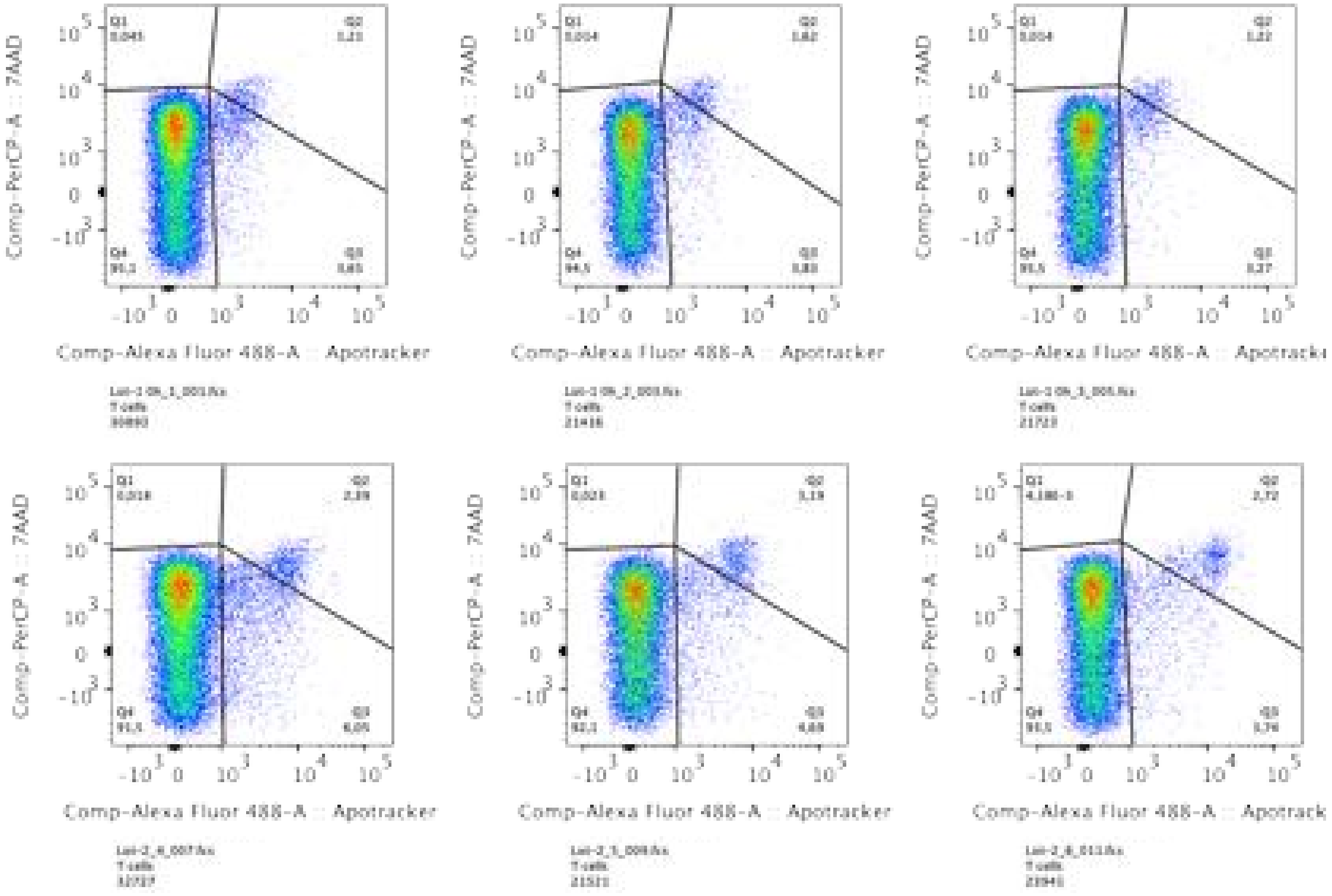

Carryover

Lot 1

Lot 2

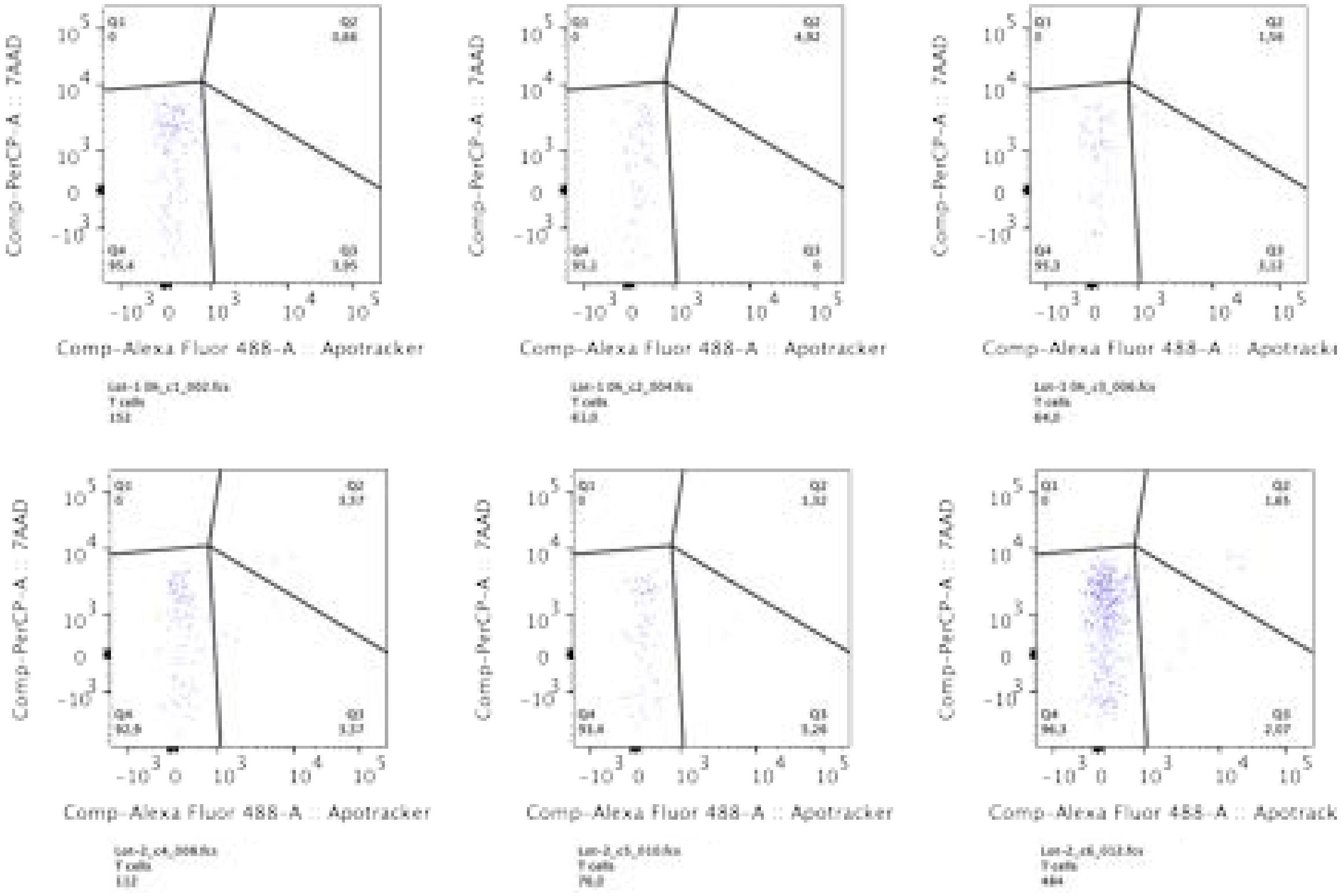

Carryover

Lot-to-lot variability

Lot 1

Lot 2

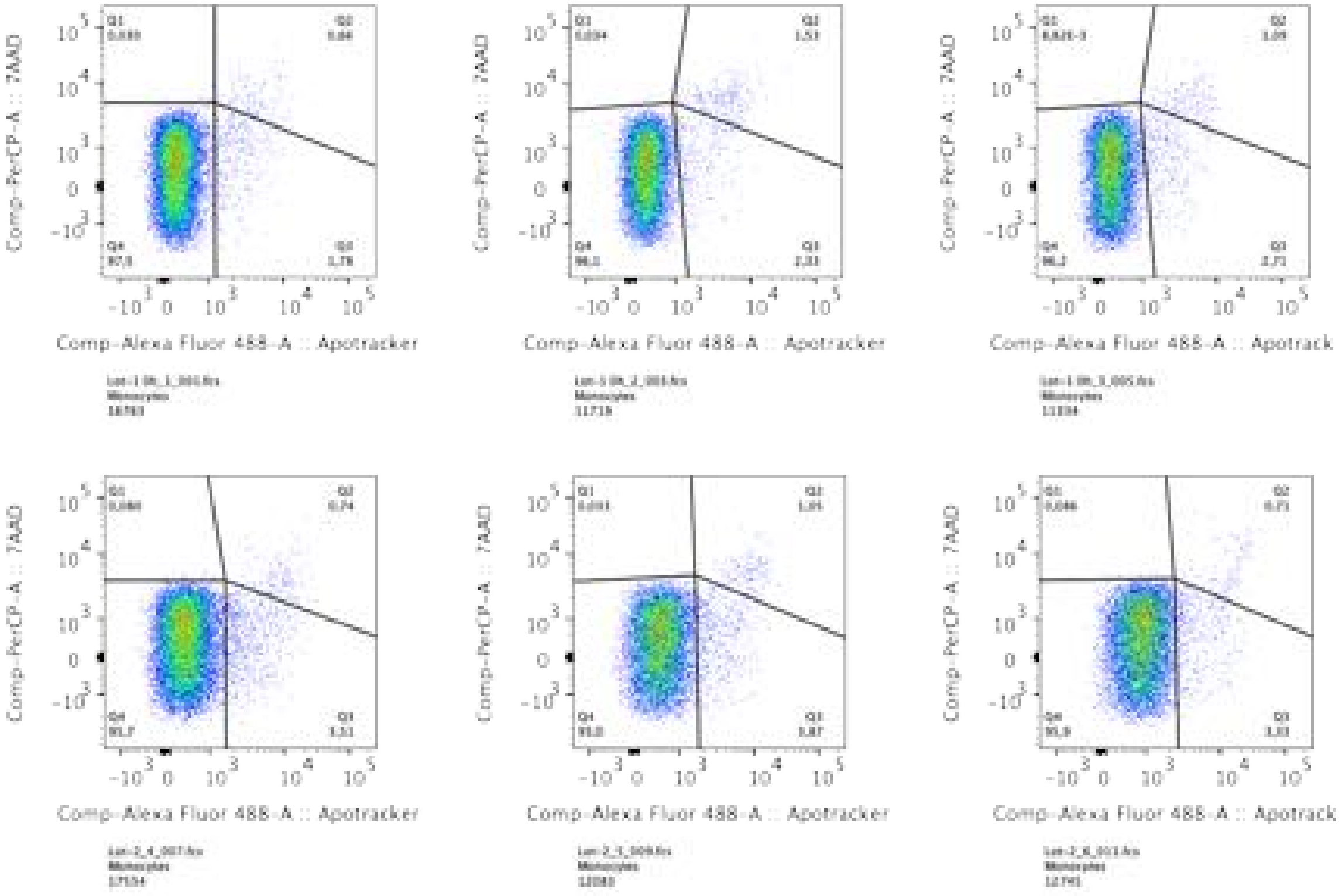

Carryover

Lot 1

Lot 2

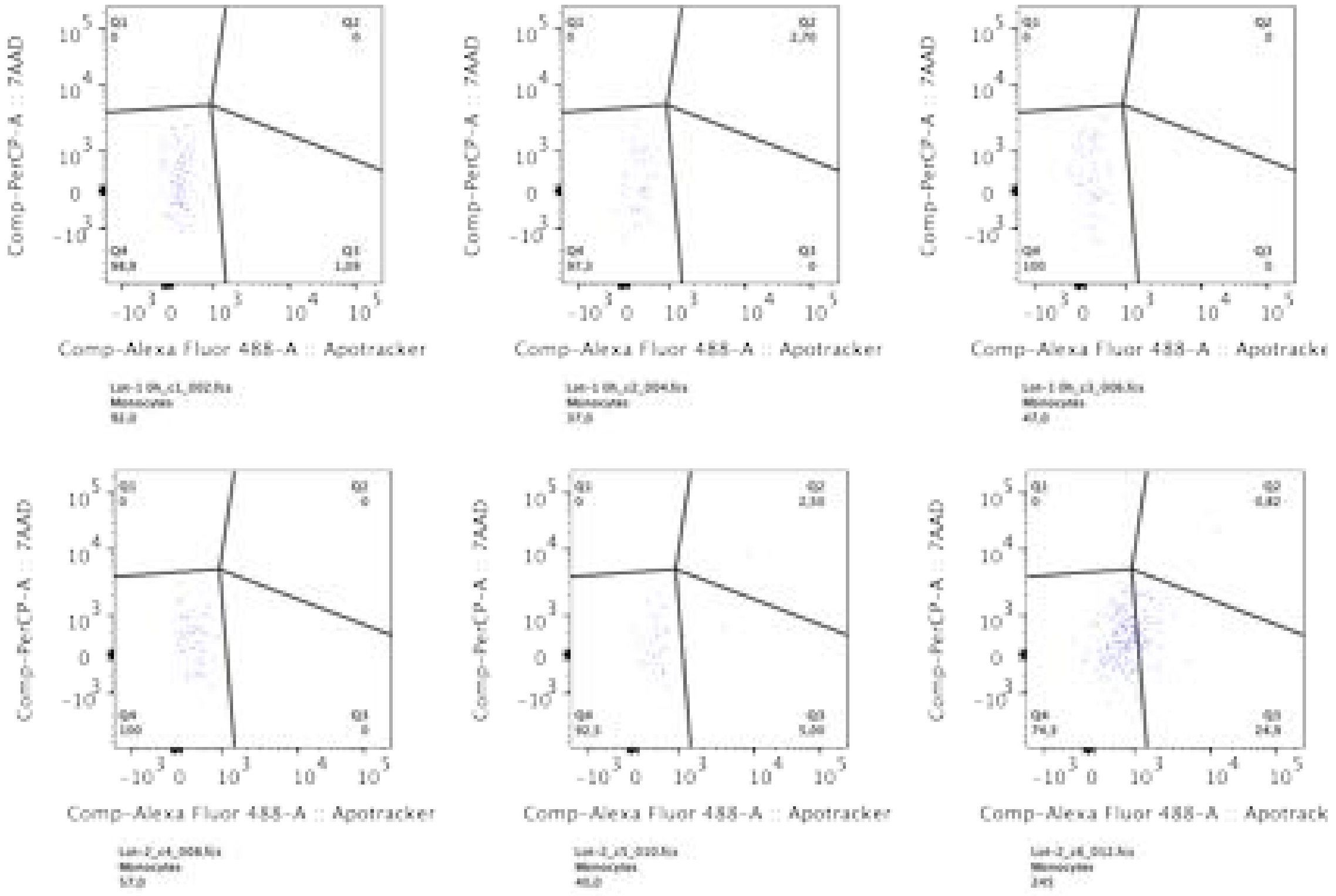

Carryover

Lot-to-lot variability

Carryover

Lot 1

Lot 2

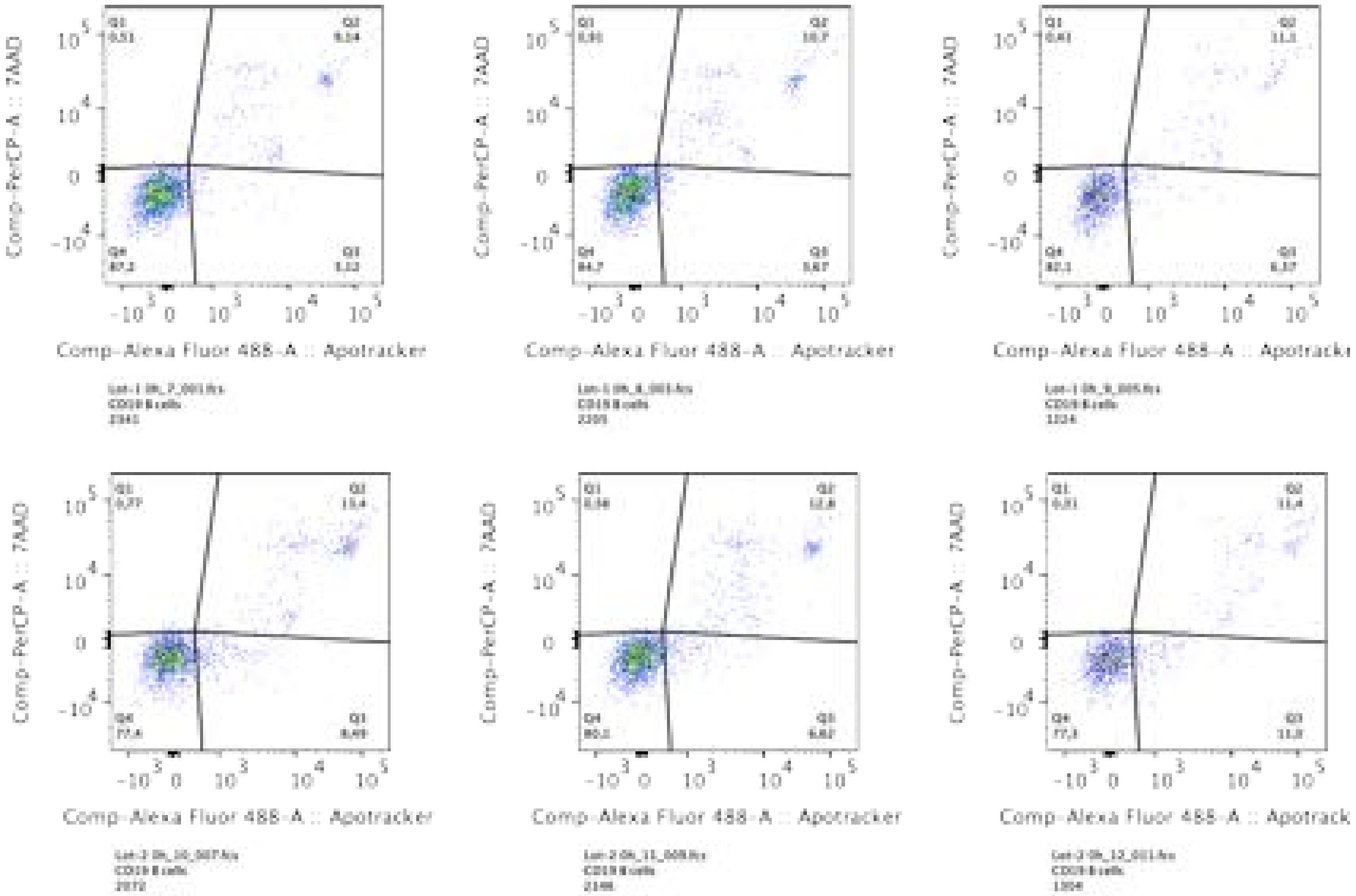

Lot 1

Lot 2

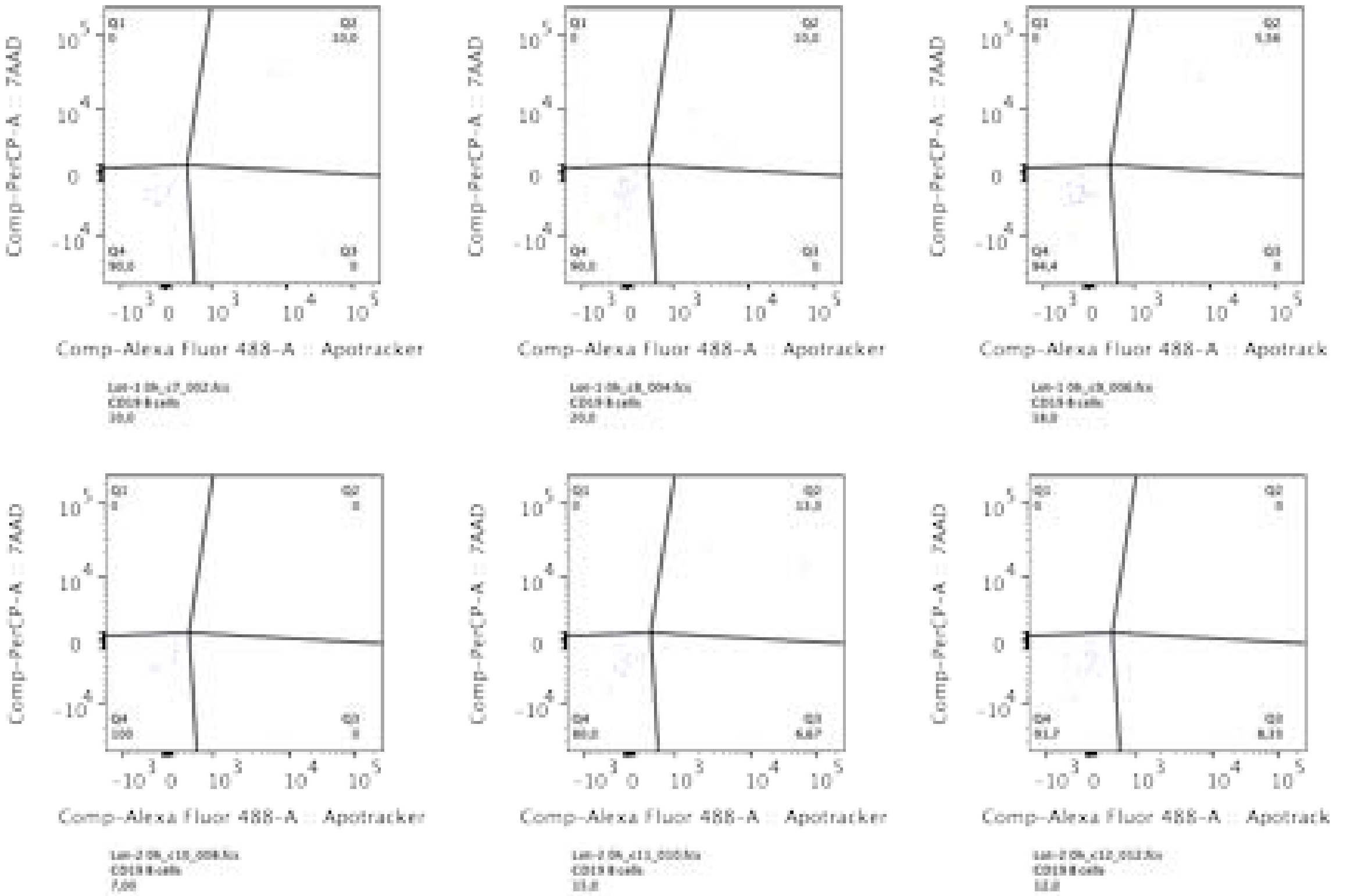

Carryover

Lot-to-lot variability

Lot 1

Lot 2

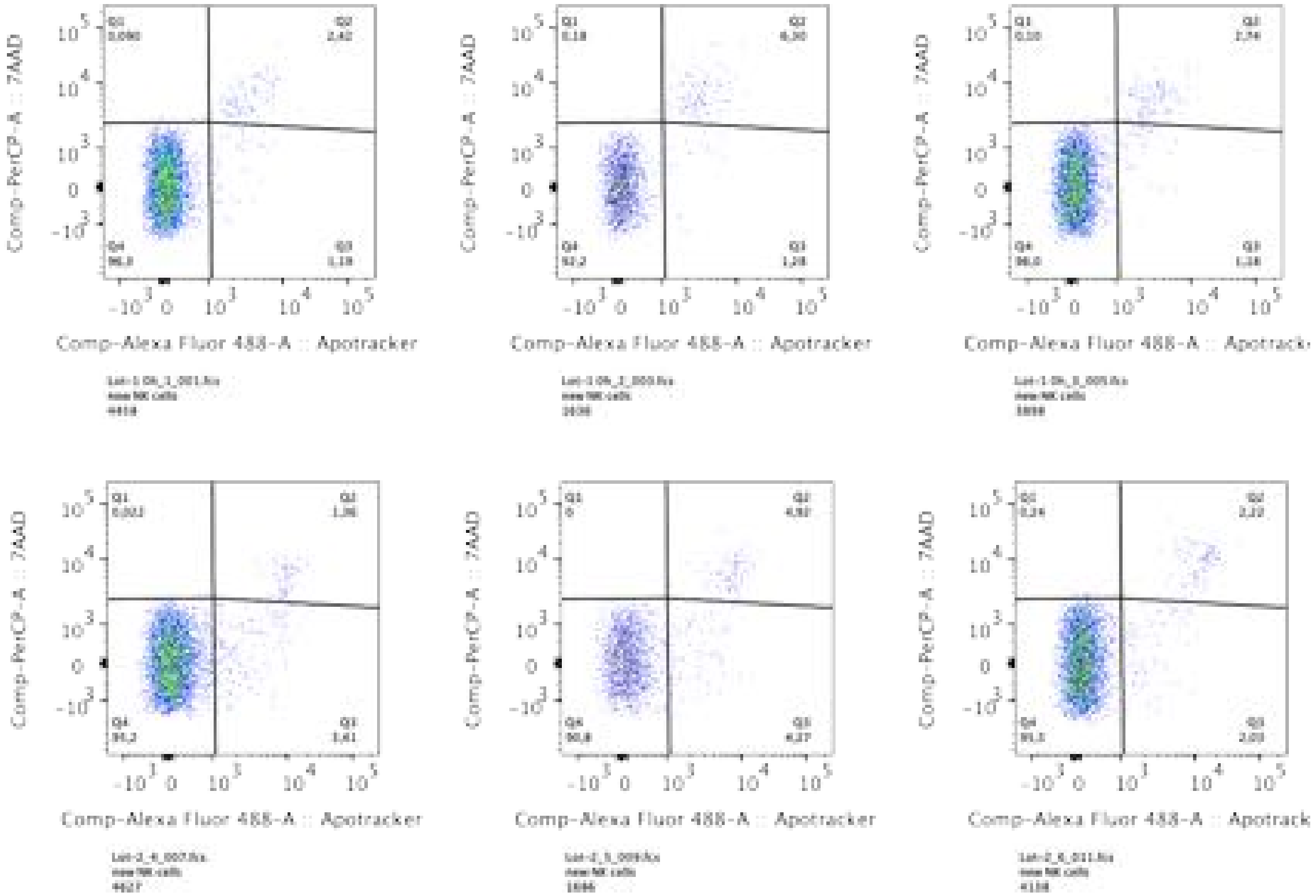

Carryover

Lot 1

Lot 2

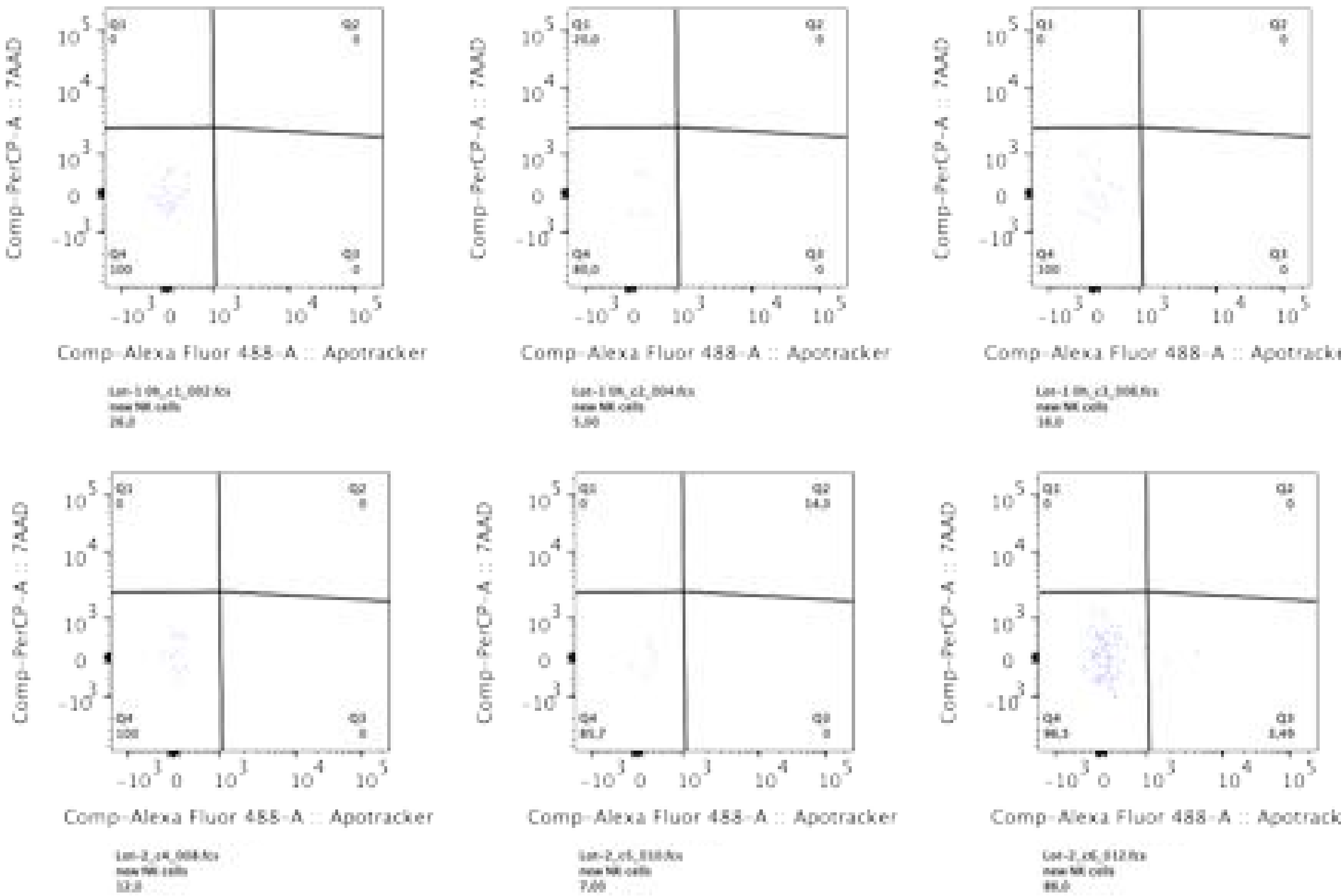

Carryover

Lot-to-lot variability

Lot 1

Lot 2

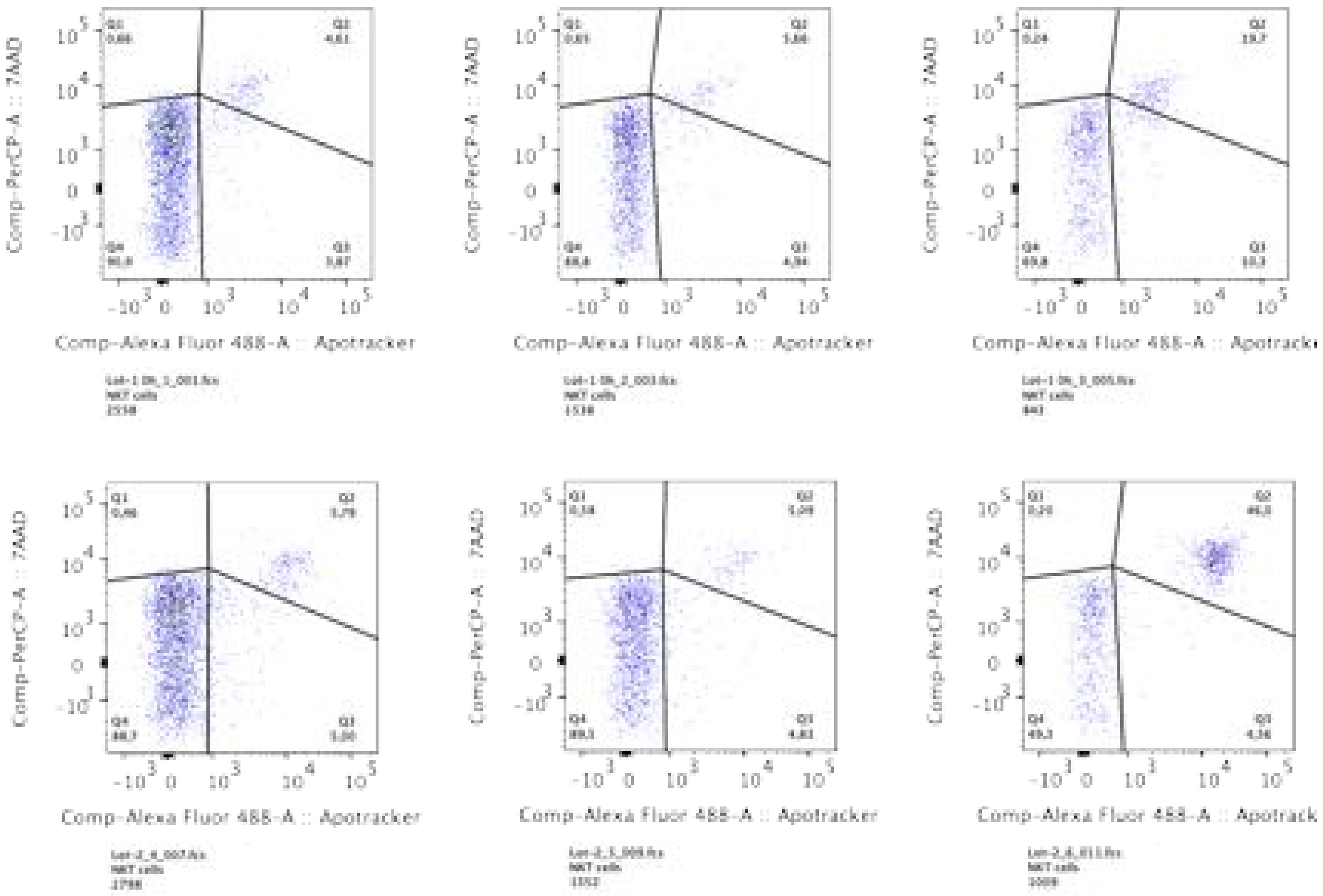

Carryover

Lot 1

Lot 2

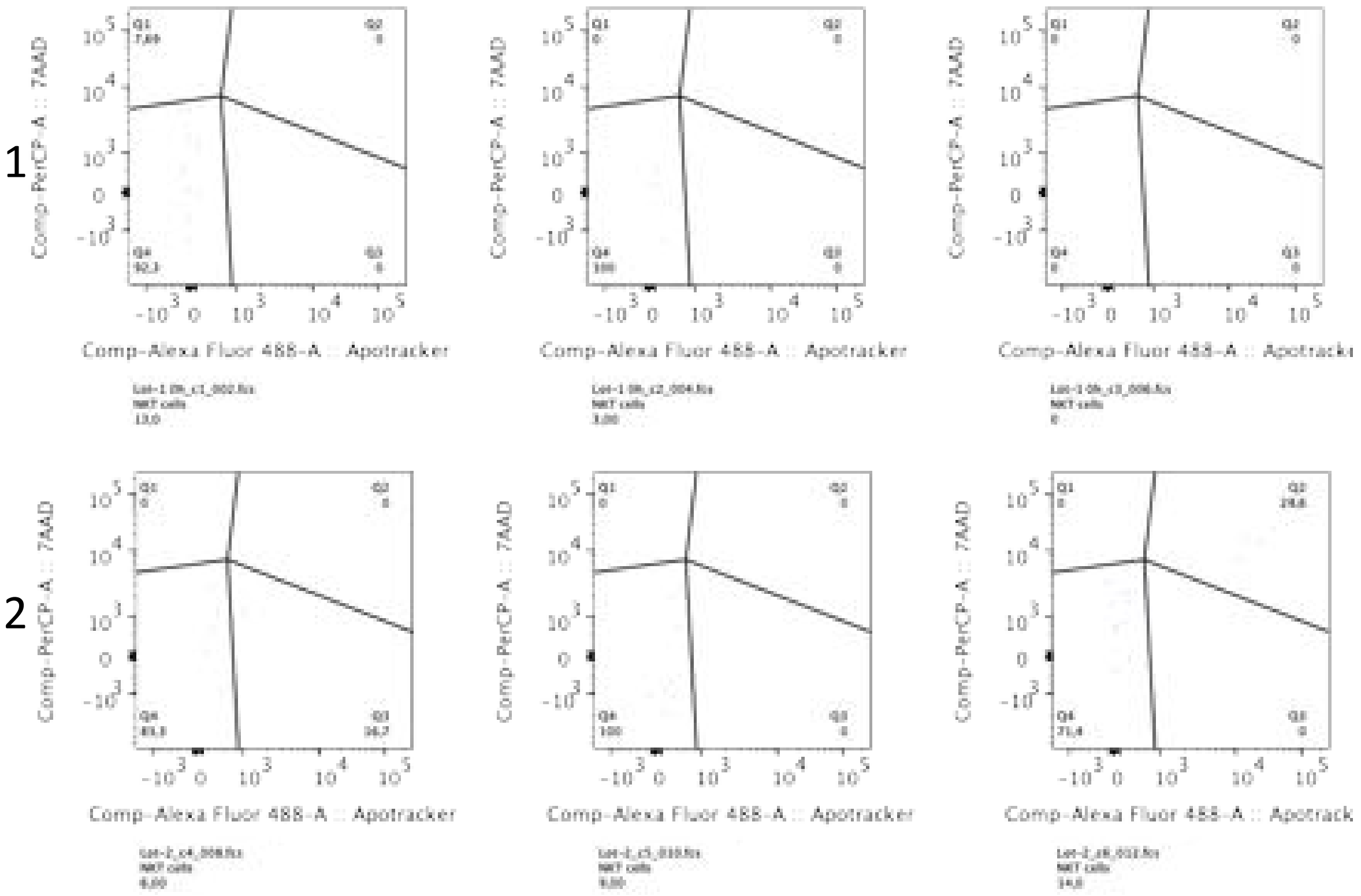

Carryover

Lot-to-lot variability

Lot 1

Lot 2

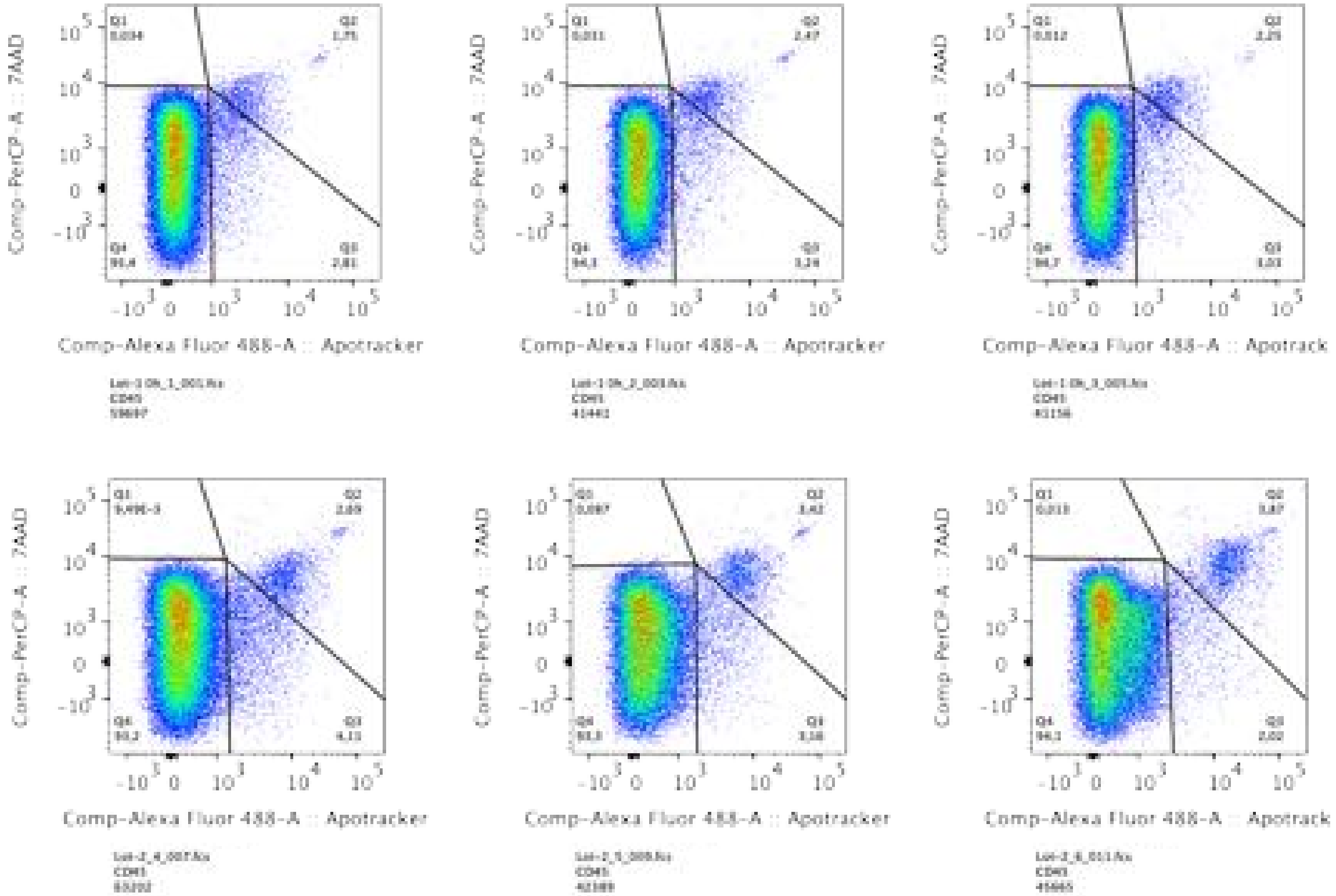

Carryover

Lot 1

Lot 2

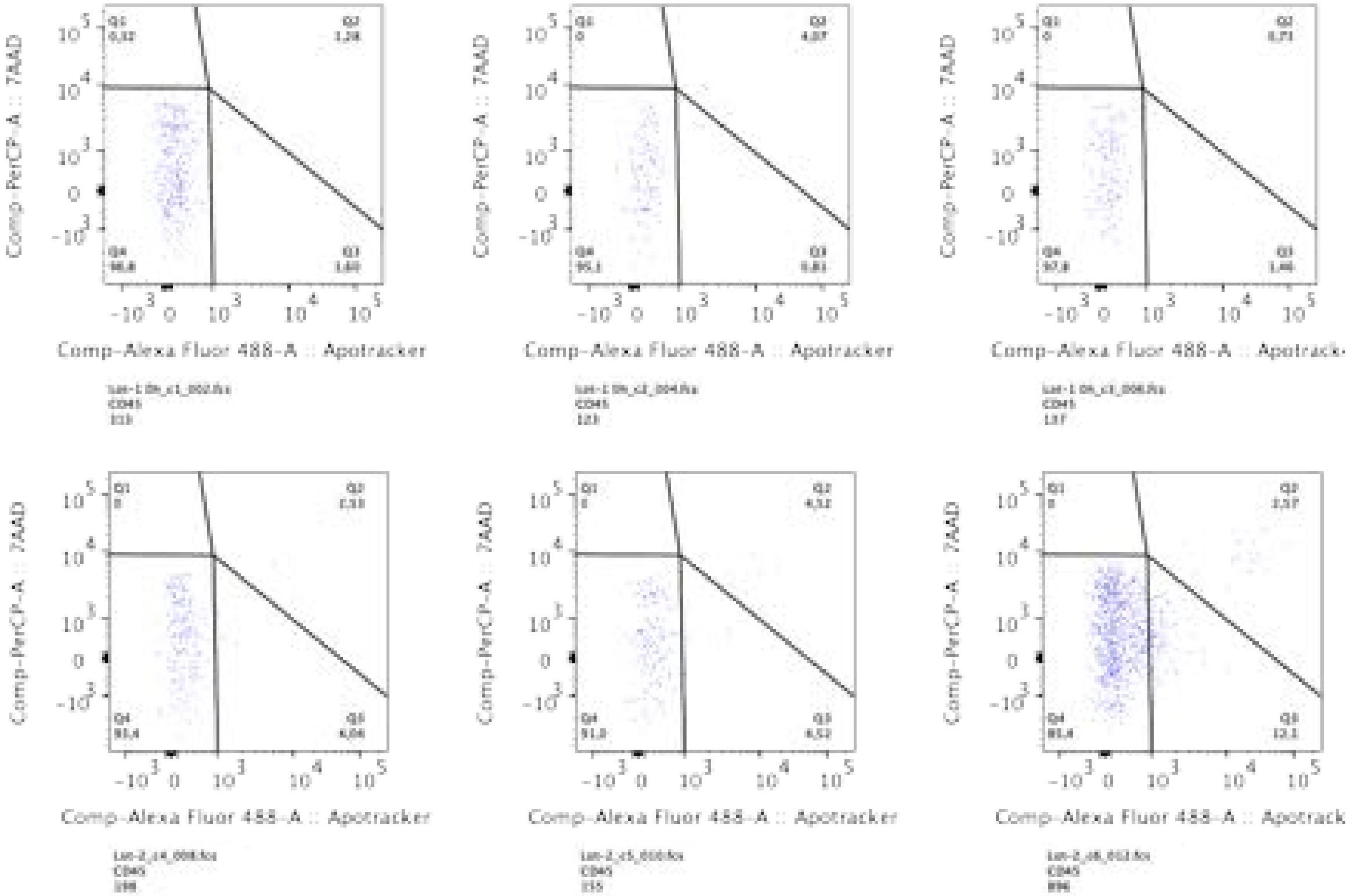

Figure S13 Representative original FACS dot-plots

Table S1. The comparison of MFI between single stained and fully stained samples

	**CD45⁺ (MFI)**	**CD14⁺ (MFI)**	**CD56⁺ (MFI)**	**CD3⁺ (MFI)**	**Apo⁺ (MFI)**	**7AAD⁺ (MFI)**
Full stained	12582	84936	1764	46985	1761	2025
CD45 single stained	13638	-	-	-	-	-
CD14 single stained	-	100337	-	-	-	-
CD56 single stained	-	-	2025	-	-	-
CD3 single stained	-	-	-	57476	-	-
Apo single stained	-	-	-	-	1725	-
7AAD single stained	-	-	-	-	-	6942
CV (%)	4.0	8.3	6.9	10.0	1.0	54.8

Table S2. The comparison of the percentage of positive cells between single stained and fully stained samples

	**$CD45^{+}$ (%)**	**$CD14^{+}$ (%)**	**$CD56^{+}$ (%)**	**$CD3^{+}$ (%)**	**Apo^{+} (%)**	**$7AAD^{+}$ (%)**
Full stained	46	14	4.04	23.4	2.3	0.97
CD45 single stained	45.8	-	-	-	-	-
CD14 single stained	-	16.3	-	-	-	-
CD56 single stained	-	-	3.64	-	-	-
CD3 single stained	-	-	-	26.7	-	-
Apo single stained	-	-	-	-	2.31	-
7AAD single stained	-	-	-	-	-	1.53
CV (%)	0.2	7.6	5.2	6.6	0.2	22.4

Table S3. System suitability assessment using BD Multi-Check Control

CD3+ T cells (%)		CD19+ B cells (%)		CD3-CD16+CD56+ NK cells (%)		CD45+CD14- cells (%)		CD45+CD14+ monocytes (%)		Operation date	Operator name	Lot. No.
**TV (R)**	**MV**	**TV (R)**	**MV**	**TV (R)**	**MV**	**TV (R)**	**MV**	**TV (R)**	**MV**			
72.8 (62.8 - 82.8)	72.1	13.3 (9.3 - 17.3)	13.2	13.1 (6.1 - 20.1)	10.8	95 (95 - 100)	97	85.1 (75.1 - 95.1)	84	2023-01-04	MH	BM0123N
72.8 (62.8 - 82.8)	71.7	13.3 (9.3 - 17.3)	12.6	13.1 (6.1 - 20.1)	10.6	95 (95 - 100)	97.1	85.1 (75.1 - 95.1)	79.9	2023-01-16	AW	BM0123N
72.8 (62.8 - 82.8)	71.7	13.3 (9.3 - 17.3)	12.9	13.1 (6.1 - 20.1)	11.1	95 (95 - 100)	97.1	85.1 (75.1 - 95.1)	86.1	2023-01-30	KM	BM0123N
73.3 (63.3 - 83.3)	71.9	13.3 (9.3 - 17.3)	13.6	11.8 (4.8 - 18.8)	10	95 (95 - 100)	96.3	89.1 (79.1 - 99.1)	91	2023-03-13	MH	BM0223N
73.3 (63.3 - 83.3)	72.4	13.3 (9.3 - 17.3)	13.3	11.8 (4.8 - 18.8)	10.1	95 (95 - 100)	95.9	89.1 (79.1 - 99.1)	86.8	2023-03-01	MH	BM0223N
75.8 (65.8 - 85.8)	73	12.4 (8.4 - 16.4)	13.6	16.4 (3.4 - 17.4)	8.6	95 (95 - 100)	98.1	88.6 (78.6 - 98.6)	81.5	2023-03-13	MH	BM0323N
75.8 (65.8 - 85.8)	70.5	12.4 (8.4 - 16.4)	13.5	16.4 (3.4 - 17.4)	8.9	95 (95 - 100)	97.6	88.6 (78.6 - 98.6)	88.9	2023-03-29	AKB	BM0323N
73.5 (63.5 - 83.5)	72.6	13.9 (9.9 - 17.9)	13.3	11.3 (4.3 - 18.3)	10.2	95 (95 - 100)	97.6	83.7 (73.7 - 93.7)	90.4	2023-04-13	MJ	BM0423N
73.5 (63.5 - 83.5)	72.9	13.9 (9.9 - 17.9)	12.9	11.3 (4.3 - 18.3)	9.8	95 (95 - 100)	98.1	83.7 (73.7 - 93.7)	87.9	2023-04-25	MJ/MH	BM0423N

Table S4. Summary of Analytical Method Validation Parameters

Validation parameter	Definition	Validation approach	Acceptance criterion	Observed result
Specificity	Ability to measure target analyte without interference from other substances.	Fully stained PBMCs versus marker-specific FMO controls; false-positive events assessed across two runs and three donors.	False-positive rate <10%.	• CD14, CD3, CD45 and CD56: <1%;<br>• apoptotic-cell populations: <10%.
Sensitivity	Ability of the assay to reliably detect and quantify low-abundance cell populations; LOB represents the background signal in blank samples, LOD the lowest level distinguishable from background, and LLOQ the lowest level that can be quantitatively measured with acceptable precision.	Serial dilution of stained cells into unstained cells. LOB and LOD were determined using blank and near-blank samples, respectively; LLOQ was established from precision across the dilution series.	LLOQ: lowest tested level with CV ≤ 30%. LOB/LOD: calculated using the predefined statistical formulas.	• T cells: LLOQ = 0.1%, LOB = 0 event, LOD = 3.4 events;<br>• Monocytes: LLOQ = 1%, LOB = 1.6 events, LOD = 3.5 events;<br>• B cells: LLOQ = 1%, LOB = 0 event, LOD = 0 event;<br>• NK cells: LLOQ = 0.1%, LOB = 0 event, LOD = 1.9 events;<br>• NKT cells: LLOQ = 10%, LOB = 0 event, LOD = 0.9 event.
Accuracy	Closeness of agreement between the measured value and an accepted reference value.	BD Multi-Check Control with manufacturer-assigned, lot-specific target values and acceptable ranges was analyzed across nine independent runs over four months, involving multiple operators and four control lots. Measured values for five leukocyte populations were compared with the corresponding manufacturer-assigned target values/ranges.	Measured values for all evaluated cell populations within the manufacturer-defined, lot-specific acceptable ranges.	Across all nine runs, measured values for all five leukocyte populations remained within their corresponding manufacturer-assigned target ranges, supporting the accuracy of the analytical method.
Linearity	Ability of the assay to generate results proportional to the expected cell number across the tested dilution range.	Serial dilution of samples in buffer. Measured cell events were normalized using counting beads and plotted against predicted cell events, followed by linear regression analysis.	$R^2 \geq 0.95$	• $R^2 > 0.95$ for all evaluated cell populations;<br>• T cells, monocytes, NK cells, and NKT cells remained linear to 1:100 dilution;<br>• B cells to 1:10;<br>• Apoptotic T cells, monocytes, B cells, and NK cells remained linear to 1:10;<br>• Apoptotic NKT cells to 1:100.
Precision (Repeatability)	Closeness of agreement among replicate measurements obtained under the same analytical conditions within a single run.	Three replicate measurements of each sample within a single analytical run; precision assessed using CV% for each reportable cell population.	CV ≤10% for major cell populations; CV ≤30% for rare/apoptotic cell populations.	• T cells, monocytes, B cells, NK cells, and NKT cells: CV <10%;<br>• apoptotic cell populations: CV <30%.

Validation parameter	Definition	Validation approach	Acceptance criterion	Observed result
Intermediate precision	Consistency of results under within-laboratory variations, including different instruments and operators.	Inter-instrument: stained PBMCs were analyzed using BD FACSCanto and BD LSR II, and variability in cell frequencies was assessed by CV%.	CV ≤10% for major cell populations.	• CV <10% for major cell populations;<br>• CV >10% for rare/apoptotic populations.
		Inter-operator: cell frequencies and MFI obtained by different operators were compared using mean difference, CV%, and relative change.	Mean difference ≤5%; CV and relative change ≤10%.	• Mean differences in cell frequency were <5%;<br>• CV and relative change were <10% for all cell subsets;<br>• MFI differences were statistically significant for some markers, although CV and relative change remained <10% in most cases.
Robustness	Ability of the analytical procedure to maintain its expected performance under deliberate variations representative of routine use.	Sample stability: Stained samples stored for 6 h at RT or 24 h at 4°C were compared with freshly analyzed samples (0 h).	Relative change within ±20% and CV ≤20%.	• CD3+ T, CD56+ NK: ≤ 20% (stable);<br>• CD19+ B, CD56+CD3+ NKT: decrease at 6 h;<br>• CD14+ mono: stable 6 h, decrease at 24 h;<br>• Apoptotic cells: increase over time (esp. > 24 h).
		Antibody cocktail stability: A pre-mixed 7-color antibody cocktail stored at 4°C for 24 h was compared with a freshly prepared cocktail.	CV <10%.	CV was <10% across evaluated cell populations, supporting cocktail stability for 24 h at 4°C.
		Lot-to-lot variability: Cell population measurements obtained using two antibody lots were compared.	CV < 10% between lots.	• T cells, monocytes, B cells, NK cells, NKT cells: within ±10%;<br>• apoptotic populations showed greater variability and exceeded the criterion.
		Carryover: Buffer tubes were inserted between sample tubes and residual events were quantified using the same gating strategy.	Carryover < 1%.	Carryover exceeded 1% with Lot 2 and exceeded 5% for some rare apoptotic populations.
		Lysing procedure: Single versus double RBC lysis of BD Multi-Check Control samples was compared based on cell frequencies and MFI.	CV and relative change ≤ 10%.	• Cell frequencies: CV < 10% (minor variation);<br>• MFI: stable except CD14 ($p < 0.001$).
System suitability	Confirmation that the analytical system, including instrument, reagents, procedure, and operator, performs adequately for its intended use.	BD Multi-Check Control with manufacturer-assigned, lot-specific target ranges was analyzed across nine independent runs over four months, involving multiple operators and four control lots. Five leukocyte populations were monitored.	Measured values for all monitored cell populations within the manufacturer-defined, lot-specific target ranges.	Across all nine runs, measured values for all five leukocyte populations remained within the corresponding lot-specific target ranges, demonstrating consistent longitudinal assay and system performance.
